# *AutoStepfinder*: A fast and automated step detection method for single-molecule analysis

**DOI:** 10.1016/j.patter.2021.100256

**Published:** 2021-04-30

**Authors:** Luuk Loeff, Jacob W.J. Kerssemakers, Chirlmin Joo, Cees Dekker

**Affiliations:** 1 Kavli Institute of Nanoscience and Department of Bionanoscience, Delft University of Technology, 2629 HZ Delft, The Netherlands

**Keywords:** single molecule, data analysis, *Stepfinder*, *AutoStepfinder*, fluorescence, nanopore, magnetic tweezer, optical tweezer, biophysics, step detection

## Abstract

Single-molecule techniques allow the visualization of the molecular dynamics of nucleic acids and proteins with high spatiotemporal resolution. Valuable kinetic information of biomolecules can be obtained when the discrete states within single-molecule time trajectories are determined. Here, we present a fast, automated, and bias-free step detection method, *AutoStepfinder*, that determines steps in large datasets without requiring prior knowledge on the noise contributions and location of steps. The analysis is based on a series of partition events that minimize the difference between the data and the fit. A dual-pass strategy determines the optimal fit and allows *AutoStepfinder* to detect steps of a wide variety of sizes. We demonstrate step detection for a broad variety of experimental traces. The user-friendly interface and the automated detection of *AutoStepfinder* provides a robust analysis procedure that enables anyone without programming knowledge to generate step fits and informative plots in less than an hour.

## Highlights

• Fast, automated, and bias-free detection of steps within single-molecule trajectories
• Robust step detection without any prior knowledge on the data
• A dual-pass strategy for the detection of steps over a wide variety of scales
• A user-friendly interface for a simplified step fitting procedure

## The bigger picture

Single-molecule techniques have made it possible to track individual protein complexes in real time with a nanometer spatial resolution and a millisecond timescale. Accurate determination of the dynamic states within single-molecule time traces provides valuable kinetic information that underlie the function of biological macromolecules. Here, we present a new automated step detection method called *AutoStepfinder*, a versatile, robust, and easy-to-use algorithm that allows researchers to determine the kinetic states within single-molecule time trajectories without any bias.

## Graphical abstract

**Figure:**
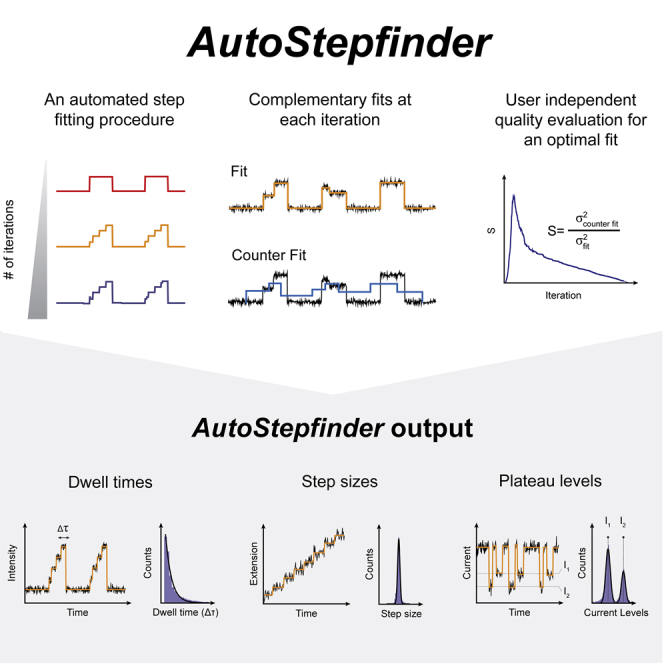

## Teaser

Loeff et al. report on an automated step detection method called *AutoStepfinder*. The algorithm allows researchers to determine the kinetic states within single-molecule time trajectories without any prior knowledge on the underlying noise contributions and step locations. A dual-pass strategy determines the optimal fit and allows *AutoStepfinder* to detect steps of a wide variety of sizes. The user-friendly interface and the automated detection of *AutoStepfinder* provides a robust analysis procedure that enables anyone without programming knowledge to generate step fits.

## Introduction

Over the last 25 years, single-molecule techniques have greatly enhanced our understanding of complex biological processes.^1^^,^^2^ These techniques have made it possible to track the molecular dynamics of individual proteins and protein complexes with a (sub)nanometer spatial resolution and a (sub)millisecond timescale.^3^^,^^4^ For example, molecular motor protein complexes were observed to move in a step-by-step fashion along cytoskeleton filaments.^5^, ^6^, ^7^ More generally, force spectroscopy (using, e.g., optical or magnetic tweezers) has been exploited as a versatile tool for probing the forces and motions that are associated with biological macromolecules.^8^^,^^9^ Single-molecule fluorescence techniques have been used to determine the stoichiometry, binding kinetics, and conformational dynamics of nucleic acids and proteins.^10^, ^11^, ^12^, ^13^ Nanopores have provided a powerful tool for the label-free detection of nucleic acids and proteins.^14^^,^^15^

Accurate determination of different states within a single-molecule time trace provides valuable information about the kinetic properties that underlie the function of biological macromolecules. For trajectories that display complex behavior, manual analysis is commonly practiced, in which a person with a trained eye picks out each state, a routine which, however, is prone to induce user bias. A common challenge in single-molecule data analysis is to distinguish these states in a reliable, reproducible, and unbiased manner. To facilitate reliable single-molecule trajectories, several automated step detection methods have been developed over the years. Initial step detection methods relied on thresholding^16^ or pairwise distribution analysis.^5^^,^^17^ While these methods are capable of detecting clear state-to-state transitions, they do not suffice when the state transitions are close to the noise level and when the data exhibit multiple steps of variable size. Alternatively, statistical modeling can be used to extract kinetic information from single-molecule trajectories (see reviews by Colomb and Sarkar^18^ and Tavakoli et al.^19^).

Early model-based step detection methods relied on the use of a generalized likelihood-ratio test to detect steps assuming Gaussian^20^ or Poissonian^21^ noise, without the need to make any kinetic model assumptions. Other statistical model-based step detection methods rely on the use of an information criterion (IC).^22^, ^23^, ^24^ The general concept of these algorithms is to generate a variety of candidate models with steps at different locations.^19^^,^^22^ Each of these models is scored on the goodness of the fit to the data, the number of used parameters in the model, and a term that penalizes each extra step that is added to the fit to prevent overfitting of the data.^22^ The optimal model for the data is subsequently selected by minimizing the IC score, resulting in a “hands-off” fitting procedure.^22^^,^^25^, ^26^, ^27^, ^28^, ^29^ For IC-based approaches, selecting the correct mathematical modeling of the noise contributions in the signal is crucial to obtain reliable fitting results, thereby requiring a full description of the noise in the data. Given that the sources of noise can vary substantially per experimental setup, it is difficult to make an IC-based algorithm that is applicable to a wide variety of trajectories.

One of the most commonly used approaches is based on hidden Markov modeling (HMM), which involves estimating the transition probabilities of a number of postulated states that are visited during the time course of an experiment. Various improvements were made to HMM, for example, by making use of a local and global HMM that allows to overcome the need for a state to successively occur within the same trajectory.^30^ While HMMs have proven to be a powerful algorithm for the analysis of single-molecule trajectories, they are limited when it comes to analyzing systems with unknown dynamics. HMMs are often used in a supervised manner where the user provides parameters, such as the number of visited states and the allowed transitions between each state.^31^, ^32^, ^33^, ^34^, ^35^, ^36^, ^37^ However, generally these parameters are unknown *a priori*^33^^,^^38^^,^^39^ and require the user to sample a parameter space to find a suitable model. In a more objective approach, HMM is combined with Bayesian nonparametrics,^40^ allowing one to use HMM without any knowledge on the number of visited states *a priori*. However, depending on the parameter space that is covered, this can dramatically increase the required computational time. In summary, HMMs are a powerful statistical tool, but it remains challenging to apply HMM models to systems with unknown system dynamics or when states are not frequently visited (e.g., bleaching data).

More recent algorithms have focused on combining model-based approaches with machine learning to allow unsupervised classification and idealization of single-molecule trajectories in a high-throughput manner.^41^^,^^42^ While machine learning algorithms provide a powerful tool for high-throughput and unsupervised processing of data across a wide range of single-molecule techniques, the underlying models need to be (re)trained to work reliably on complex single-molecule trajectories.^41^^,^^42^ Therefore, there is a need for explorative approaches, hereafter called first-order approaches, that are not tailored to a specific noise model and do not require information on the underlying states *a priori*. Such first-order approaches provide flexibility in interpreting and analyzing features in single-molecule trajectories and can provide important input for machine learning and model-based algorithms.

Previously, Kerssemakers et al. reported on a first-order approach called *Stepfinder*.^20^ Given its robustness, flexibility, and simplicity, *Stepfinder* received great interest in the field of biophysics and was applied to the experimental trajectories from a wide variety of biological systems.^43^, ^44^, ^45^, ^46^, ^47^, ^48^, ^49^, ^50^ Despite its popularity, the algorithm faced several caveats: (1) the algorithm was subject to user bias, requiring the user to determine the final number of steps, (2) it was computationally demanding when presented with large datasets, (3) it failed in step evaluation when presented with data that exhibited a broad spectrum of step sizes, which especially holds true for baseline-type trajectories, and (4) it lacked a user-friendly interface. To overcome these short-comings, we here present a significantly revised and superior algorithm (*AutoStepfinder*) that facilitates high-throughput and automated step detection.^51^^,^^52^

*AutoStepfinder* has been designed as a first-order analysis tool that requires minimal knowledge of the location of steps and the various signal contributions in the data. A central feature of both *Stepfinder* and *AutoStepfinder* is the application of two complementary fits with an equal number of steps. To do so, this algorithm iteratively fits steps at locations that yield the biggest reduction in the variance (σ^2^). Subsequently, the σ^2^ of the fit is compared with the σ^2^ of a worst-case-fit with the same number of steps, called a counter fit.^43^^,^^51^^,^^52^ To assess the quality of the fit, the algorithm generates a step spectrum or S-curve that displays a sharp peak when the signal harbors step-like features, whereas smooth changes (such as drift) result in a flat S-curve. In contrast to *Stepfinder*, *AutoStepfinder* assesses the quality of the fit over multiple rounds, allowing for step analysis at various scales. This multiscale step fit procedure allows the researcher to evaluate which steps are relevant for the analysis.

The *AutoStepfinder* algorithm is a first-order step detection approach that provides a survey of the step-landscape and is complementary to more refined statistical methods that require a full description of the experimental parameters. The user-friendly interface of *AutoStepfinder* simplifies step detection in single-molecule trajectories, making it accessible to a wide variety of users. We illustrate the effectiveness of *AutoStepfinder* with a variety of different experimental traces from diverse single-molecule techniques. Taken together, the *AutoStepfinder* algorithm can be regarded as a robust and versatile tool that can be used for the many experimental cases where a full description of the noise in the data is unavailable.

## Results

### Overview of the procedure

**Figure 1:**
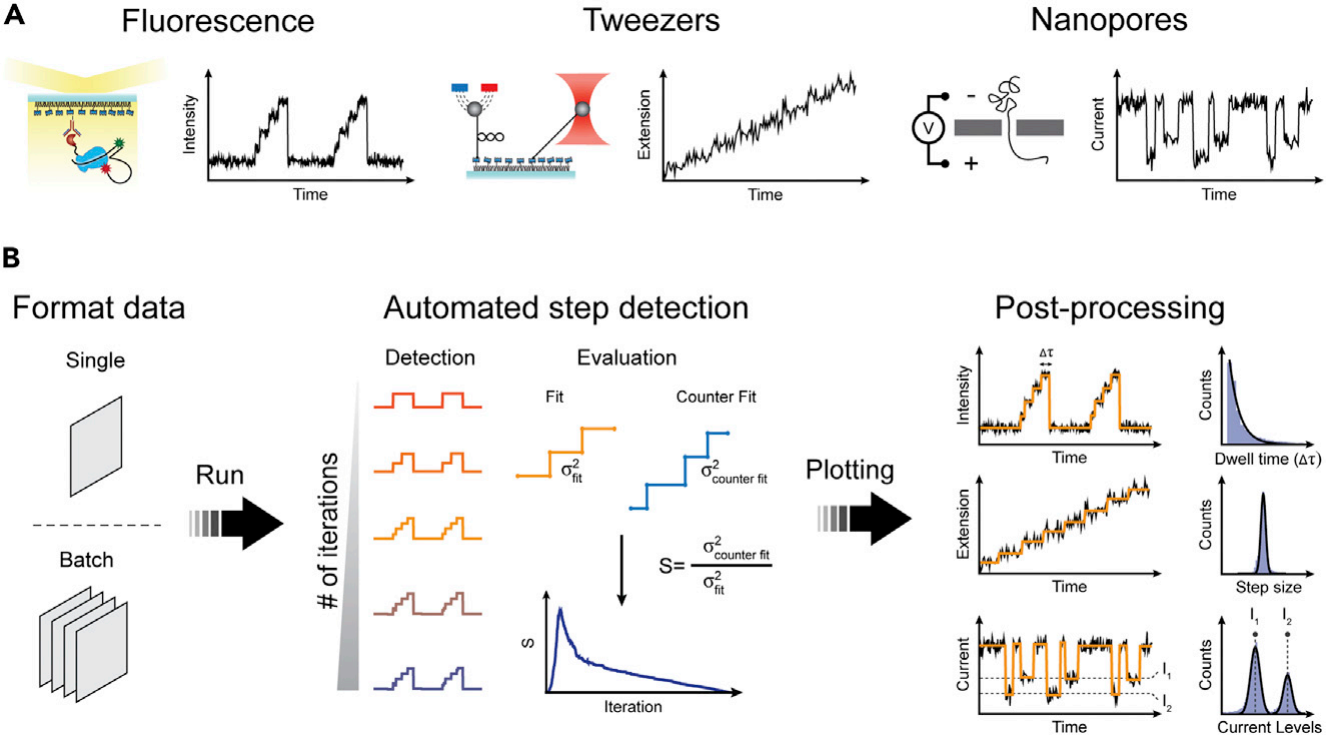
Workflow of *AutoStepfinder* (A) *AutoStepfinder* can be applied on a wide variety single-molecule of trajectories, including single-molecule fluorescence, magnetic and optical tweezers, and nanopore data. (B) The algorithm requires input in the form of one or multiple.txt files with one or two columns (signal value or time and signal value). After pressing run, the algorithm iteratively adds single steps to the data that minimizes the σ^2^ value. For each iteration, the quality is assessed by means of a secondary counter fit. Finally, the best fit is selected and the algorithm outputs the corresponding fit, dwell times, step sizes, and levels. Fitting large datasets (>10^6^ data points) can be done in less than 1 min with a desktop computer. Also see Figure S1.

The workflow of the *AutoStepfinder* algorithm is outlined in Figure 1. *AutoStepfinder* can analyze the trajectories of a wide variety of single-molecule techniques (Figure 1A). The step-finding algorithm functions in three main steps: input of data, automated step detection, and output of the result (Figures 1B and S1). The *AutoStepfinder* algorithm can run on a single data file or on multiple files using a batch mode. Once input is retrieved, *AutoStepfinder* runs a first round of step fitting, in which the algorithm minimizes the variance (σ^2^) between the data and the fit. During this procedure the data are iteratively split into multiple plateaus (Figure 1B). At each iteration, a new plateau is fitted at a location that yields the biggest reduction in σ^2^. After each partition event, *AutoStepfinder* determines the quality of the fit by performing an additional fit (called a counter fit)^43^^,^^51^^,^^52^ (Figures 1B and S1).

Once the most prominent steps are fitted during the first round of fitting and counter fitting, the *AutoStepfinder* algorithm subtracts the optimal fit from the data and executes the second round of iterative fitting and counter fitting on the residual data (Figure S1). This dual-pass strategy allows *AutoStepfinder* to facilitate step fitting of data with steps that vary widely in size. Once the optimal fit for the second round is determined, the algorithm generates a final fit by combining the step indices of the two rounds of fitting, and it outputs several files that allow post-processing of the results (Figures 1B and S1). This dual-pass step-fitting method provides a robust approach for automated step detection. The user-friendly interface and the automated detection of *AutoStepfinder* provides a hands-off fitting procedure that can be executed by anyone without programming knowledge.

### Principles of step detection

**Figure 2:**
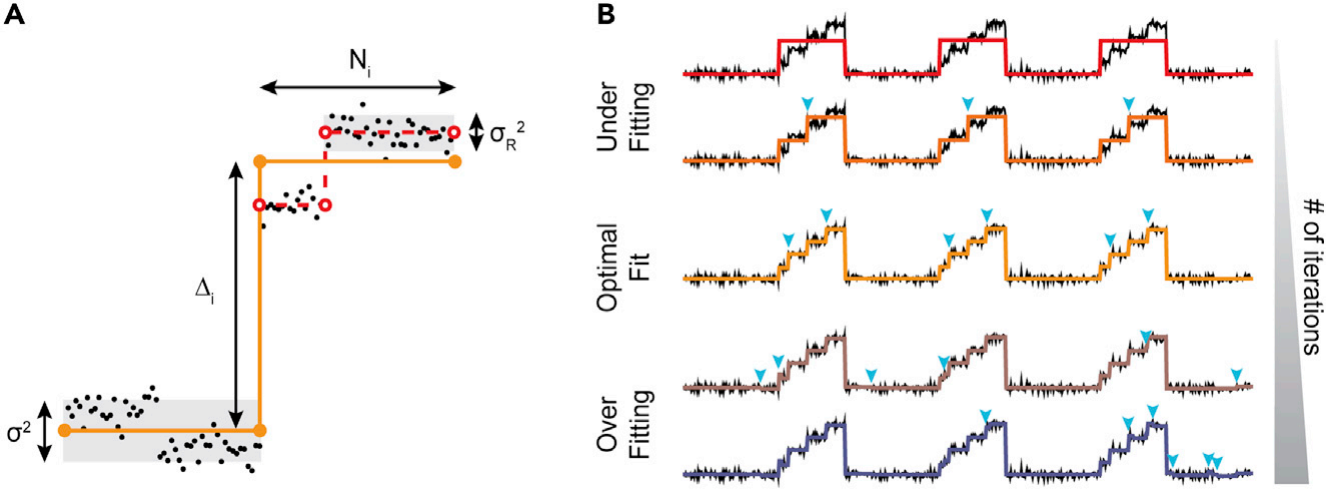
Global arrangement of the *AutoStepfinder* algorithm (A) An example of an iterative step fit (orange line) on a single-molecule trajectory (black dots). Single-molecule trajectories are fitted by the *AutoStepfinder* algorithm by iteratively minimizing the σ^2^ value. To perform a step fit the program assumes that the data contain steps (Δ_i_), bounded by a plateau (N_i_) that is subject to residual noise (σ_R_^2^, gray box). After the first step fit, *AutoStepfinder* selects the plateau with the largest value of σ^2^, for the next partition event (red dashed lines). This process continues until the maximum number of iterations is reached. (B) An example of the iterative process of step fitting by the *AutoStepfinder* algorithm. The algorithm successively adds a single step to the data (cyan triangles) and thereby minimizes the σ^2^ value. Step fitting below the optimal number of steps is considered underfitting, whereas step fitting beyond the optimal number of steps is considered overfitting. Also see Figure S1.

The *AutoStepfinder* algorithm fits data through a series of partition events that minimize σ^2^ (Figure 1). To fit data, the algorithm makes the sole assumption that the data contain instantaneous steps of interest with variable size (Δ_i_) and plateau length (N_i_). These plateaus are exposed to “noise,” which can be defined as the residual variance (σ_R_^2^) from random signals that arise from the experimental setup (true noise) or features at a different scale that are not of interest (Figure 2A). The algorithm initiates the fitting procedure by fitting one step to all data points at a location that gives the lowest value of σ^2^. This initial partition event generates a fit with two plateaus at a position that represents the average of the data points within the plateau (Figure 2A).^43^ After the first fit, the plateau that exhibits a step yielding the largest reduction σ^2^ is selected for the next partition event, resulting in a fit with three plateaus (Figure 2A, dashed red line). The algorithm continues this process of adding a single step to one of the plateaus for each iteration (Figure 2B, cyan arrow heads), until *AutoStepfinder* has performed the user-defined maximum iteration number (Figure S1).

*AutoStepfinder* successively selects one of previously fitted plateaus for the next partition event based on the biggest reduction in σ^2^ (Figure 2A). This makes *AutoStepfinder* a so-called greedy algorithm that makes a locally optimal choice without considering its effect on the next step fits (see comparison of AutoStepfinder with other methods). Because *AutoStepfinder* iteratively prioritizes the next fit that gives the biggest reduction in σ^2^, the most prominent features of the data are fitted first, followed by fits for the more refined features. The iteration continues until the user-defined number of steps is reached. Typically, this number is large enough so that the step fits is likely to go beyond any “optimal fit” (Figure 2B, middle). This results in “overfitting,” where new steps are fitted to the noise of the data (Figure 2B, bottom).

### Probing a step fit spectrum

**Figure 3:**
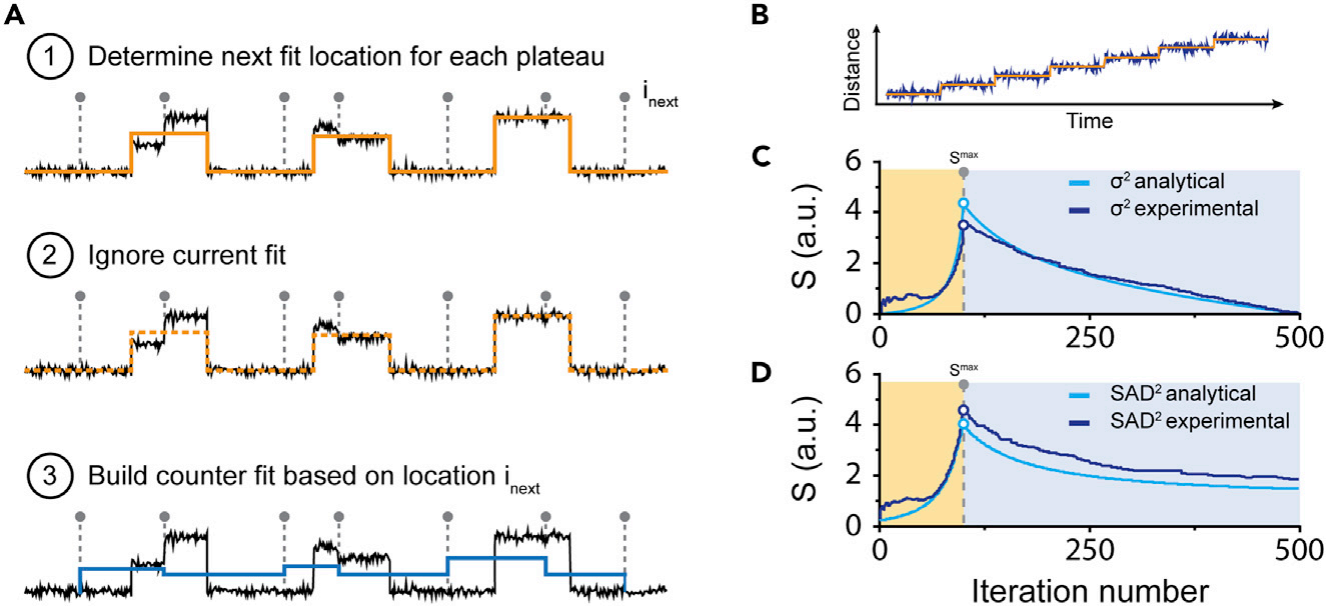
Determining the quality of a step fit (A) For every step fit the algorithm performs, the quality of the fit (orange line) is evaluated by means of an additional fit (blue line, called a counter fit). The counter fit is built by determining the next partition point (i_next_), after which the current is rejected. Subsequently, the algorithm places the counter fit (blue) plateaus at locations within the existing fit (orange). (B) Simulated trajectory representing a motor stepping behavior. (C) Representative example of experimental and analytical S-curves obtained by fitting the trajectory in (B) through minimization of σ^2^. Shaded areas indicate the underfitting (yellow) and overfitting (light blue) regime. (D) Representative example of experimental and analytical S-curves obtained by fitting the trajectory in (B) through minimization of the sum of absolute differences (SAD^2^). Shaded areas indicate the underfitting (yellow) and overfitting (light blue) regime. Also see Figure S2.

Next, to determine the optimal fit for a given dataset, it is important to evaluate the quality of the fit for every step that is added to the analysis (Figure 1). The quality of the existing fit is evaluated by performing an additional fit for each iteration, hereafter called a counter fit.^43^ *AutoStepfinder* generates such a counter fit by means of three steps: (1) *AutoStepfinder* determines the next partition location (i_next_) within each plateau (Figure 3A); (2) the algorithm ignores the existing step locations; and (3) *AutoStepfinder* builds a new fit based on the i_next_ locations, generating new plateaus with a position that represents the average of the data points within each plateau (Figure 3A). These three steps result in a counter fit with steps that are all located in between the existing best-fit locations (Figure 3A). If the analyzed data do not display step-like behavior, both the existing fit and counter fit will have similar values of σ^2^ (Figure S2).^43^^,^^51^^,^^52^ However, when the data do display step-like behavior, counter fitting results in a fit that is much worse than the existing fit (Figure 3A) and thereby yields a larger value of σ.^2^^43^^,^^51^^,^^52^ To evaluate the quality of a fit, the *AutoStepfinder* algorithm takes advantage of the sharply changing σ^2^ landscape upon counter fitting.

The quality of a fit (S-score), can be quantified by taking the ratio of the σ^2^ value from the existing fit and the counter fit, which is defined as:

$$S=\frac{{\text{σ}}_{counter\;fit}^{2}}{{\text{σ}}_{existing\;fit}^{2}}\text{.}$$

If the existing fit is at the optimal number of iterations, the variance of the existing fit approximates the residual variance in the data (σ^2^). In contrast, the counter fit misses all real steps and places step location fits at random plateau positions. Thus, the fit values differ on average ½Δ from the data plateaus, yielding a σ^2^ of the counter fit that reaches its maximum value (∼Δ^2^/4σ_R_^2^).^43^ Thereby, the maximum S-value (S^max^) can be described by S^max^ = 1 + P, where P equals the maximum value of the counter fit (Δ^2^/4σ_R_^2^).^43^ The strong difference of σ^2^ between the fit and the counter fit when an optimal number of iterations is reached results in an S-value that is much larger than 1 (Figure 3). In contrast, when the data are under-fitted, the σ^2^ value of the counter fit and existing fit approximate each other, resulting in an S-value that is close to 1 (Figure 3B). Similarly, overfitting a dataset, in which steps follow the noise, only results in a marginal change in the σ^2^ value of the counter fit and thus the S-value also becomes close to 1 (Figures 2B, 3B, and 3C). Therefore, the S-curve is an effective indicator for stepped behavior in the analysis (Figure 3B). In effect, the S-curve provides an assessment of the step fit spectrum, displaying the scales at which steps occur in the signal as prominent peaks.

The use of two orthogonal fits allows *AutoStepfinder* to highlight features that evoke a strong discrepancy in the fit and counter fit S-values, which correspond to the most step-like features in single-molecule trajectories. The discrepancy between the two fits allows *AutoStepfinder* to use σ^2^ minimization for very different kinds of noise types without a significant decay of its performance. To display this behavior, we generated an additional version of *AutoStepfinder* that uses a different error signal: the sum of absolute differences (SAD), and compared its behavior with *AutoStepfinder*, which uses minimization of the variance (σ^2^). To compare the two algorithms, we generated trajectories that mimic stepped behavior of a kinesin-like motor protein with n_s_ steps of 8 nm that are bounded by plateaus with N_w_ points (Figure 3B). Comparison of the two versions of *AutoStepfinder* yielded S-curves with a sharp peak that was located at an identical global maximum (Figures 3B–3D). Moreover, the simple nature of this signal allowed us to provide analytical approximations of the S-curves. The analytical solution of the S-curve using the variance for over and under fitting can be approximated by:

$$S_{f<1}\left(f\right)=\frac{P\left[\frac{2+f}{3}\right]+1}{2P\left(1-f\right)+1}\;;\;S_{f>1}\left(f\right)=1+\frac{P}{f}+\frac{\left(1-1/f\right)}{4N_{f}}\text{,}$$

where N_f_ is the average location of the plateau, P = Δ^2^/4σ_R_^2^, and the relative fit fraction (f) can be described by f = n_i_/n_s_, where n_i_ is the actual number of fitted steps. In this equation the correct number of steps corresponds to f = 1. The analytical solution of the S-curve using the SAD can be approximated by:

$$S_{f<1}\left(f\right)=\frac{P_{\omega }\left(\frac{2+f}{3}\right)}{2P_{\omega }\left(1-f\right)+\left(2f-1\right)};\;S_{f>1}\left(f\right)=1+\frac{P_{\omega }}{f}+\frac{1-1/f}{2N_{f}}-\frac{1}{f}\text{,}$$

where P_ω_ = Δ/2ω. These analytical solutions result in S-curves that reflect the observed trends for the two experimental S-curves (Figures 3C and 3D). Taken together, this comparison suggests that the exact nature of the residual noise does not need to be known for effective step fitting with *AutoStepfinder*. Thereby, *AutoStepfinder* constitutes an inherently robust first-order step analysis tool.

### Step detection over a wide spectrum of sizes by a dual-pass strategy

**Figure 4:**
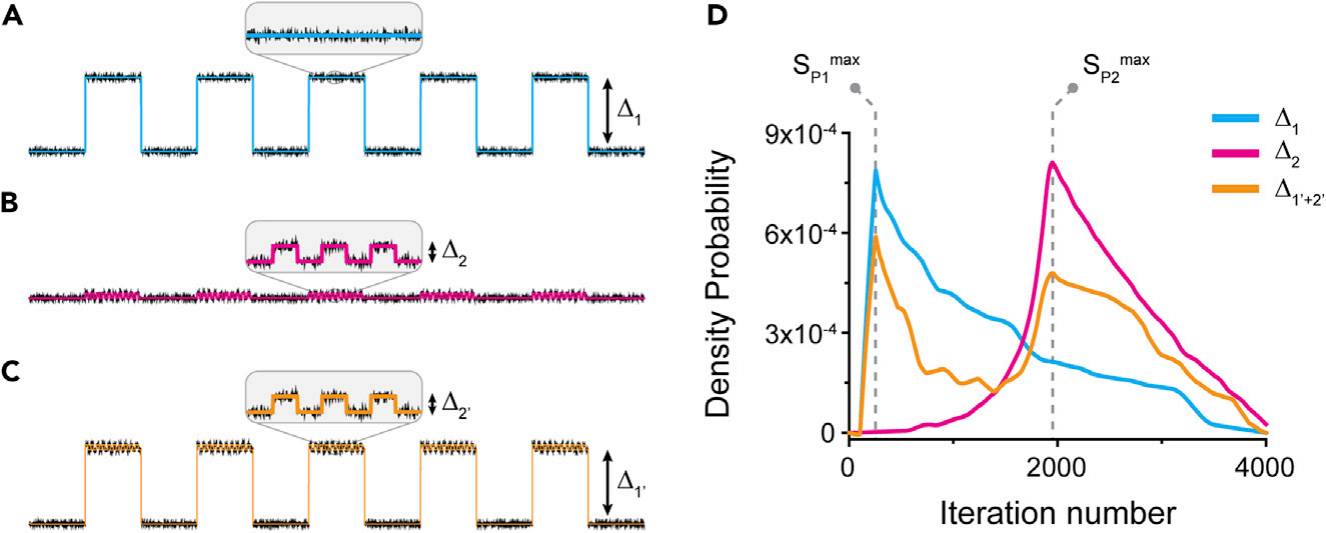
Dual-pass step detection to detect a wide range of step sizes (A) A simulated single-molecule trajectory displaying uniform steps with a size of Δ1. (B) An example trace displaying uniform steps with a size of Δ_2_. (C) A simulated single-molecule trajectory displaying non-uniform steps with a size of Δ_1’_ and Δ_2’_. (D) S-curves for the three example traces displayed in (A–C). The global maximums of peak 1 (S_P1_^max^) and peak 2 (S_P2_^max^) are indicated with dashed gray lines. The S-curve for the dataset with both large (Δ_1_) and small (Δ_2_) steps exhibits two peaks.

Both *Stepfinder* and *AutoStepfinder* use the S-curve as a robust measure to determine the quality of a fit, showing a distinct peak when the optimal number of iterations is reached. When the steps in the data are within the same order of magnitude (e.g., Δ_1_ or Δ_2_ in Figures 4A and 4B), the *Stepfinder* algorithm would plot the S-curve and require the user to select the optimal fit by providing the number of iterations that corresponds to the global maximum of the S-curve (S^max^) (Figure 4D). However, the optimal number of iterations cannot be determined through S^max^ when the data exhibit steps that vary widely in size. Especially when large and small steps are combined in a single-trajectory (e.g., Δ_1’_ and Δ_2’,_ Figure 4C), the S-curve may exhibit multiple peaks or shoulders (S_P2_) that have a lower S_P2_^max^ than the first peak (S_P1_^max^) (Figure 4D). Notably, the position of these peaks is identical to the peaks observed for a dataset with either Δ_1_ or Δ_2_ (Figure 4D). In this case, the previous version of the algorithm (*Stepfinder*) is thereby not capable of suggesting an optimal fit for the data.

To facilitate step detection across a wide variety of scales, we developed a dual-pass strategy that determines the optimal fit for the data over two rounds (Figure S1). The *AutoStepfinder* algorithm first performs a step fit based on the global maximum of the S-curve (S_P1_^max^) that corresponds to the most prominent features in the data. This step fit is then subtracted from the data and a secondary step fit is performed on the "residual data." Only if the global maximum of the secondary step fit is above the user-defined threshold, coined “acceptance threshold,” will the fit be accepted (Figure S1). The dual-pass approach combined with the acceptance threshold on the second round of fitting provides a robust method for automated step detection. Note that one might ponder yet deeper levels of refinement, with a third round of step fitting or beyond. We have explored this, but so far never encountered a case where more than two rounds were required. Note that under these circumstances, the existence of multiple peaks in the S-curve displays multiple scales of step sizes. Thereby, the S-curve functions as a step fit spectrum with peaks indicating different step size scales or marked step behavior. Depending on the experimental context, some of these scales may be of more or less of interest to the user.

### Computationally efficient step detection with *AutoStepfinder*

*AutoStepfinder* is a so-called greedy algorithm^53^ that iteratively selects an existing plateau (N_w_) and splits it into a left (N_L_) and a right (N_R_) plateau (Figure S3A) at a location that results in the biggest reduction in σ^2^. As a result, *AutoStepfinder* makes a locally optimal choice without considering its effect on the next step fits, significantly reducing the amount of computing power that is required to determine the fit. The position of these newly acquired plateaus is strongly dependent on the location of the partition point within N_w_ (Figure S3A). Therefore, *AutoStepfinder* calculates the average position (A) of a plateau (e.g., N_L_) for any given location (i), which can be described by:

$$A_{L}=\frac{1}{N_{L}}\sum_{i=1}^{N_{L}}x\left(i\right)\text{.}$$

These positions can be used to generate a σ^2^ landscape that shows cusps at the optimal fitting positions (Figure S3B). While misplacement of a step fit affects the slope of the remaining cusps, the minima positions remain identical, implying that the location of a step fit is not affected by prior and subsequent step fitting. The robustness of the cusp location justifies the use of a greedy procedure for step fitting by *AutoStepfinder*.

Despite the greedy nature of the algorithm, the iterative process of determining the partition point requires a substantial amount of computing power and becomes problematic when analyzing large datasets (e.g., >1 × 10^6^ data points) (Figure S3C). Previously, *Stepfinder* determined the next partition point of N_w_ by calculating the σ^2^ value for all possible locations (i), selecting the step fit that would yield the largest reduction in the σ^2^ value. However, this meant that for a dataset with N_0_ data points, the algorithm performed N_0_^2^ single x(i) operations to determine a single partition point. Next, the algorithm would repeat the same cycle to generate the next left (N_L_) and right (N_R_) plateau. With this scheme, this required ½N_0_^2^ single x(i) operations to locate the next two partition points. This cycle of partitioning continued to deduce plateaus until the algorithm reached the maximum number of iterations. In total, this yields a factor of (1+½+¼+ …) ·N_0_^2^ or roughly 2·N_0_^2^ operations per dataset. Thereby, the required computing time increased significantly with an increase in the number of data points in a dataset (Figure S3C).

To reduce the operations that are required to fit a dataset, we comprehensively re-organized the code and streamlined the iteration process. A strong reduction in the number of required operations (i) can be made by re-using the information that is obtained during the localization of the first partition point. After the algorithm has determined the average (A_w_) value of a plateau (N_w_), *AutoStepfinder* determines the location of both N_L_ and N_R_ for x(i), using a single operation. The procedure starts with x(1) that is located at the left side of N_w_ (Figure S3A). The location (A_L_) of N_L_ can be deduced by A_L_(i) = x(i), whereas the level of N_R_ is defined by:

$$A_{r}\left(\text{i}\right)=\frac{\left(N_{w}\cdot A_{w}-x\left(i\right)\right)}{\left(N_{w}-1\right)}\text{ .}$$

This procedure is repeated for the next location (i + 1) until each location of N_w_ is calculated, requiring only N_0_ operations per plateau. For a whole dataset, this scales with 2·N_0_, which is a gain of a factor of N_0_ compared with the previous algorithm. Depending on the size of the analyzed dataset, this improvement yields a significant speed gain of several orders of magnitude (Figure S3B).

### Quantifying the detection limits of *AutoStepfinder*

One of the major limiting factors in the detection of step-like behavior in single-molecule trajectories is noise, which can have various origins, such as thermal fluctuations of the biological system and the electronics of the measurement system (shot noise, thermal noise, 1/f noise).^54^, ^55^, ^56^ Therefore, both the nature and the amount of noise in the single-molecule trajectories is highly dependent on the technique used to acquire the data. In its simplest form, the noise in single-molecule trajectories can be approximated as random white Gaussian noise, which can be characterized by the standard deviation of the noise.^26^ As the noise intensity (SD) (Figure 2A) increases relative to the step size, step detection becomes significantly more challenging (Figure S4). Notably, to estimate the performance of *AutoStepfinder*, we compare the signal to noise ratio (SNR), which can be defined as: SNR = ½Δ/SD, where Δ represents the step size.

**Figure 5:**
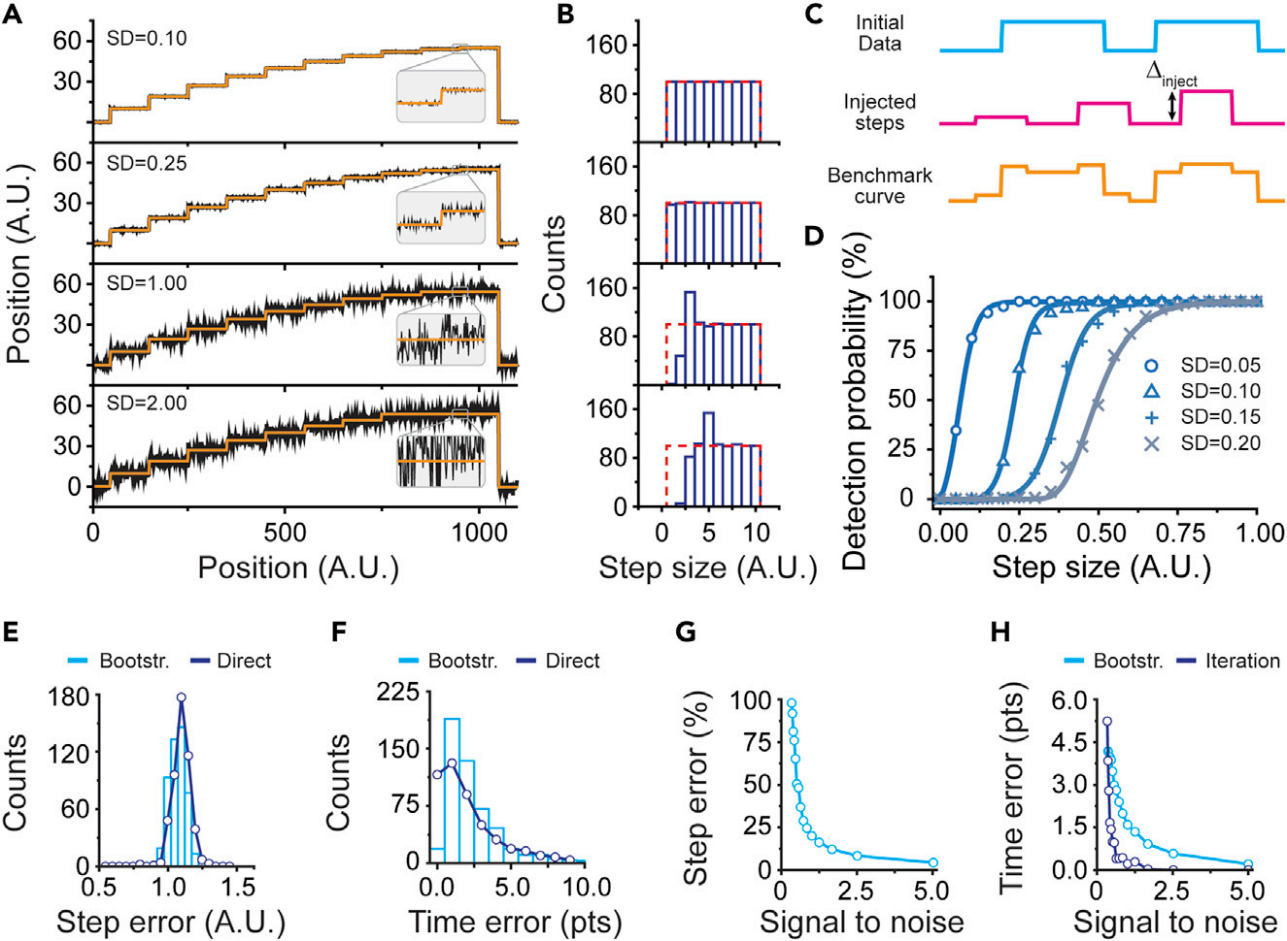
Testing the detection limits of *AutoStepfinder* (A) Simulated time trajectories that were exposed to various noise levels to benchmark the *AutoStepfinder* algorithm. The data start with a step of 10 arbitrary units (a.u.), the subsequent steps decrease by 1 a.u. until the smallest step size of 1 a.u. is reached. This idealized trajectory was repeated 100 times, resulting in a dataset in which each step size occurred 100 times. (B) Distribution of step sizes of the simulated trajectories, obtained through the *AutoStepfinder* algorithm. The red dashed lines indicate the position of each bin when 100% of the steps are correctly identified. C) Schematic of the step injection test. To quantify the probability that *AutoStepfinder* would detect steps with a certain size (Δ_inject_), steps were injected (pink curve, middle) into an existing trajectory (blue curve, top) to generate a benchmark curve (orange curve, bottom). (D) Histogram of the detection probability of step sizes at various noise levels (SD). Solid lines represent sigmoidal fits to the data. (E) Histogram showing the distribution of the 95% confidence intervals of the step sizes (cyan bars) obtained by bootstrap analysis. The line (purple) indicates the deviation of the fit from the ground truth. (F) Histogram showing the distribution of the 95% confidence intervals of the plateaus lengths (cyan bars) obtained by bootstrap analysis. The line (purple) indicates the deviation of the fit from the ground truth. (G) Relation between the SNR and the error in the determined steps. (H) Relation between the SNR and the error in the determined plateaus (cyan line). The purple line indicates the deviation between the final fit and a local refit at various noise levels (iteration error). Also see Figure S4.

To probe the detection limitations of *AutoStepfinder*, we simulated data that was composed of a signal that featured a systematic decrease in step size. The data start with a step of 10 arbitrary units (a.u.), the subsequent steps decrease by 1 a.u. until the smallest step size of 1 a.u. is reached (Figure 5A). This idealized trajectory was repeated for 100 times, resulting in a dataset in which each step size occurred 100 times. Next, this dataset was exposed to various levels white of Gaussian noise (SD) and fitted with the *AutoStepfinder* algorithm (Figure 5A). When *AutoStepfinder* detects all states within the idealized trajectory, a histogram of the distribution of step sizes should be equally populated in each bin (Figure 5B, red dashed line).

For the idealized trajectories that were subject to noise with an SD of 0.1 and 0.25, *AutoStepfinder* correctly identified >98% all the steps in the trajectory (Figure 5B). However, when the SD of the noise was equal to the smallest step size in the trajectory (SD = 1.0, SNR = 0.5), *AutoStepfinder* detected only 2% of the smallest step size of 1 a.u. and ∼50% of the steps that were 2 a.u. in size (SNR = 1.0). As a consequence of the missed steps in this regime, *AutoStepfinder* overestimated (∼150%) steps that were 3 a.u. in size. This trend continued when the SD of the noise was twice the size of the smallest step in the trajectory (SD = 2.0, SNR = 0.25) (Figure 5B).

To further quantify the response of *AutoStepfinder* to noise, we generated several benchmark traces (Figure 5C, bottom) at different noise levels by injecting steps with various sizes (Δ_inject,_ Figure 5C, middle) at known locations into an existing trajectory (Figure 5C, top). To generate statistically relevant data, this process was repeated 100 times for each noise level, randomizing Δ_inject_ between the values 0 and 1. We subsequently quantified the probability that *AutoStepfinder* would detect the injected steps (Figure 5D). *AutoStepfinder* shows a sharp cutoff in its detection probability (Figure 5D), which shifts toward larger step sizes when noise is increased and smaller steps are drowned in the noise. We note that our conservative choice of short-lived plateaus (<50 data points) increases the associated error within the steps. Under these circumstances, *AutoStepfinder* is effective (i.e., deduces steps with a >50% detection probability) in detecting steps that are twice the size of the SD of the noise (Δ_detected_ = ½SD, SNR = 1). We note that, to estimate the reliability of the obtained results, the step injection test can also be applied to experimental data, as we previously reported in a case study (see Eeftens et al.^51^).

To determine the uncertainty in the placed steps, we implemented a bootstrap analysis function^57^ in *AutoStepfinder* that closely follows the error estimation by Li et al.^58^ In brief, once *AutoStepfinder* has determined the fit, each plateau is resampled (typically 1,000 times) by bootstrap analysis and the relative positions between neighboring plateaus is re-assessed, allowing the algorithm to determine the 95% confidence interval of the step sizes. In addition, this procedure provides a confidence interval of the variance, which allows for a direct estimation of the 95% confidence interval of the step time (Figure S3B, shaded areas).^58^ As a result, prominent steps that are associated with a sharp cusp in the variance have smaller 95% confidence intervals, whereas steps with a broad minimum are associated with larger errors. To validate this approach, we simulated trajectories that mimic the stepping behavior of a motor protein (Figure 3B) at an SNR of 1 and compared the bootstrapped confidence intervals with the deviation of the *AutoStepfinder* output to the absolute solution (hereafter called direct error). This validation shows that the bootstrapped confidence intervals provide an accurate estimation of the errors associated with the step sizes (Figure 5E) and step times (Figure 5F).

We further benchmarked the *AutoStepfinder* algorithm and probed how the error landscape develops when the amount of noise increases. As expected, the 95% confidence intervals associated with the step size (Figure 5G) and step time (Figure 5H) increase when the SNR becomes smaller. To assess if the greedy nature of *AutoStepfinder* causes deviation from the true location of the fitted steps, we redetermined the optimal fit locally and compared its location with the locations in the final fit by *AutoStepfinder* (hereafter called iteration error). These results show that the greedy step search provides an accurate description of the stepped behavior in the trajectories. Based on the results of these benchmarks, we conclude that *AutoStepfinder* provides an accurate fit down to an SNR of 0.75.

While noise is the major determinant for step detection, the frequency of occurrence of steps may also influence capability of *AutoStepfinder* to detect steps. As described in the section “Principles of step detection.” *AutoStepfinder* selects the next step fit (R_next_) based on a ranking system, choosing the step that yields the largest reduction in σ^2^. This ranking system is based on the expected squared relative accuracy, which can be defined as:

Rnext=Δ21NL+1NR,

where Δ corresponds to the step size and N_L_ and N_R_ correspond to the number of data points in the left and right plateau, respectively. Consequently, fits with a large step size (Δ) or a large window size (N_i_) are prioritized. This has important implications when fitting trajectories that have a long baseline. Since *AutoStepfinder* considers the baseline as a plateau, it prioritizes long baselines for step fitting when the data exhibit a sparse density of short events. Thereby, *AutoStepfinder* may not detect these sparse events. As a rule of thumb, it is advised to truncate the dwell time of the base line when it is >10 times longer than the dwell time of the events. For optimal fitting results, we advise to use a baseline with a duration that is in the same order as the dwell time of the events.

### Comparison of *AutoStepfinder* with other methods

**Figure 6:**
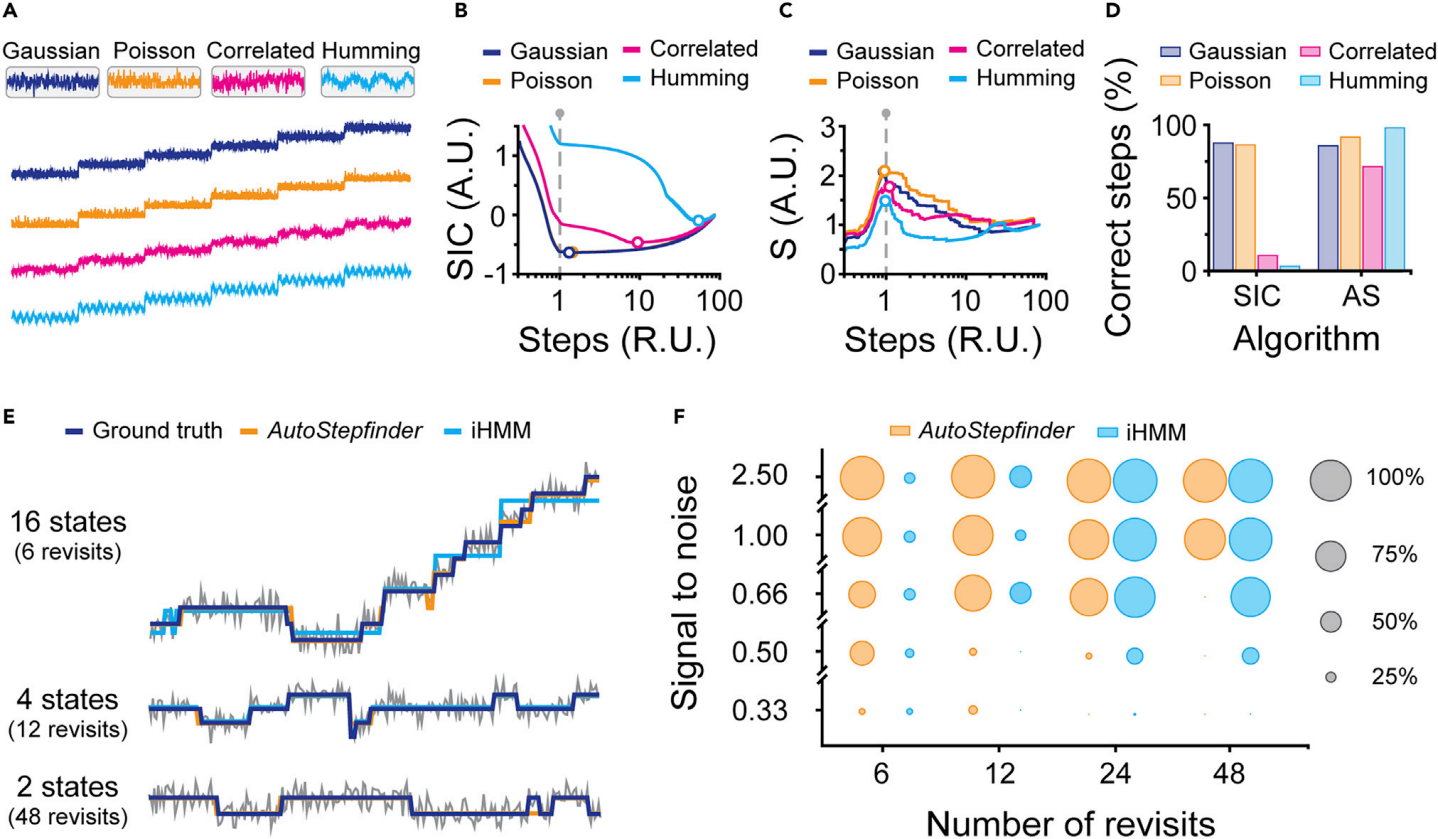
Comparison of *AutoStepfinder* with other methods (A) Examples of simulated single-molecule trajectories that were exposed to distinct noise types, each with an SD of 2.0. The noise types are Gaussian noise (purple), Poissonian noise (orange), correlated noise (pink), and humming noise (light blue). (B) Step detection by a Schwarz information criterion (SIC)-based algorithm.^22^ For each step fit, the quality of the fit is evaluated by calculating an SIC score. The SIC curve displays a minimum when the optimal fit is reached (circle). The dashed gray line indicates the number of steps in the data. Notably, the SIC curve of Gaussian noise (purple) overlaps the SIC curve of the Poissonian noise (orange). (C) Step detection by the *AutoStepfinder* algorithm. For each step fit, the quality of the fit is evaluated by performing an additional fit, called counter fit, and calculating an S-score. The S-curve displays a maximum when the optimal fit is reached (circle). The dashed gray line indicates the number of steps in the data. (D) Performance of the *AutoStepfinder* algorithm and SIC-based algorithm on simulated single-molecule trajectories that were exposed to distinct noise types with SD = 2.0. A more extensive overview on the robustness of *AutoStepfinder*- and the SIC-based algorithms^22^ is provided in Figure S4. (E) Examples of simulated single-molecule trajectories each with a distinct number of states (gray). The purple, orange, and cyan lines indicate the ground truth, states found by *AutoStepfinder*, and the states found by iHMM,^33^ respectively. The displayed trajectories were exposed to Gaussian noise with an SNR of 1.0. (F) Comparison of *AutoStepfinder* (orange) and iHMM^33^ (cyan). The size of the circles indicates the percentage states that were within a distance of 25% of a step size of the ground truth. The circles in gray indicate the percentage scale. Also see Figure S5.

The *AutoStepfinder* algorithm was designed as a robust fitting tool that provides a first-order fitting approach of experimental trajectories where a full mathematical description of the noise in the data is unattainable. To benchmark *AutoStepfinder* against a wide variety of noise types, we simulated data featuring a signal that systematically varied over time in a stepwise manner (Figure 6A). This signal was exposed to four different types of noise with the same SD: Gaussian noise, Poisson noise, and two other noise artifacts that are commonly found in single-molecule trajectories (Figure 6A): correlated noise that results in irregular correlated features, and humming noise, e.g., often associated with a line frequency (Figure 6A). Apart from *AutoStepfinder*, we used a Schwarz IC (SIC)-based algorithm^22^ tailored to Gaussian noise in our benchmark.

For the simulated data that were exposed to Gaussian noise, both *AutoStepfinder* and the Gaussian-based SIC algorithm^22^ fitted a similar number of steps across the range of noise (SD) tested (Figures 6 and S5A–S5C). Both algorithms correctly identified 98% of the steps at low noise levels (SD = 0.2), which decreased to approximately 50% at when the noise level was increased (SD = 2.0) (Figures 6D, S5D, and S5H). Thus, under ideal conditions, where a full mathematical description of the noise is available (i.e., for conditions optimal for SIC), *AutoStepfinder* performs equally well as compared with SIC-based algorithms. Next, the benchmark was repeated on trajectories with Poissonian, correlated, and humming noise. When subjected to these trajectories, *AutoStepfinder* was still capable of correctly identifying the states of interest in the data with any of these types of noise (Figures 6C, 6D, and S5). Notably, when we repeated the benchmark with the Gaussian-based SIC algorithm,^22^ the analysis yielded strongly diverging results as expected (Figures S4 and S5). These results show that *AutoStepfinder* performs robustly over a broad spectrum of noise types.

Next, we compared the performance of *AutoStepfinder* against infinite HMM (iHMM),^33^ a hands-off HMM-based algorithm that is developed to determine a limited number of states in a trajectory without making parameter assumptions *a priori*.^33^ To benchmark both algorithms, we generated trajectories with a number of states (N) that have a mean step size of 10 a.u. between them. At each time point within the state there is a 4% chance of making a transition to a higher state and a 1% chance of a transition to a lower state. This model allows one to generate a diverse set of trajectories by only changing the number of states that are present within the data (Figure 6E). For example, a low number of states implies that the chance that a state is revisited is high, generating trajectories that resemble the output of single-molecule fluorescence measurements. In contrast, for a large number of states the chance of revisiting a state becomes low, resulting in trajectories that resemble the motor stepping behavior in magnetic and optical tweezer measurements.

To compare *AutoStepfinder* and iHMM over a wide variety of signals, we generated trajectories that were limited in time with various amounts of revisits per state (6, 12, 24, and 48 times). In addition, we subjected the trajectories to different amounts of Gaussian noise, ranging from an SNR of 0.25 up to 1.5. To obtain output from *AutoStepfinder* that mimics HMM, we performed k-means clustering on the output of *AutoStepfinder*, which clusters the fitted levels into the same number of initial states as were used for iHMM. Next, we compared the output of both algorithms on each trajectory against the ground truth. All states that deviated more than 25% of a step size of the ground truth were rejected, whereas the states within 25% of a step size of the ground truth were counted as detected.

This benchmark shows that *AutoStepfinder* performs robustly and independently from the number of revisits of each state, detecting <95% of the states in the low-noise regime. For trajectories that contained only a limited number of revisits per state (Figure 6F, 6 and 12 revisits) *AutoStepfinder* outperformed the iHMM algorithm. In contrast, the performance of the iHMM algorithm increased with an increased number of revisits per state. For trajectories where a state was frequently revisited (Figure 6F, 48 revisits), iHMM outperformed *AutoStepfinder* by correctly detecting more states at a higher noise level. In conclusion, we show that *AutoStepfinder* and iHMM are complementary algorithms, where *AutoStepfinder* is favored for trajectories with limited state-to-state transitions, and where HMM is favored for trajectories with many state-to-state transitions.

### Step fitting of experimental data

**Figure 7:**
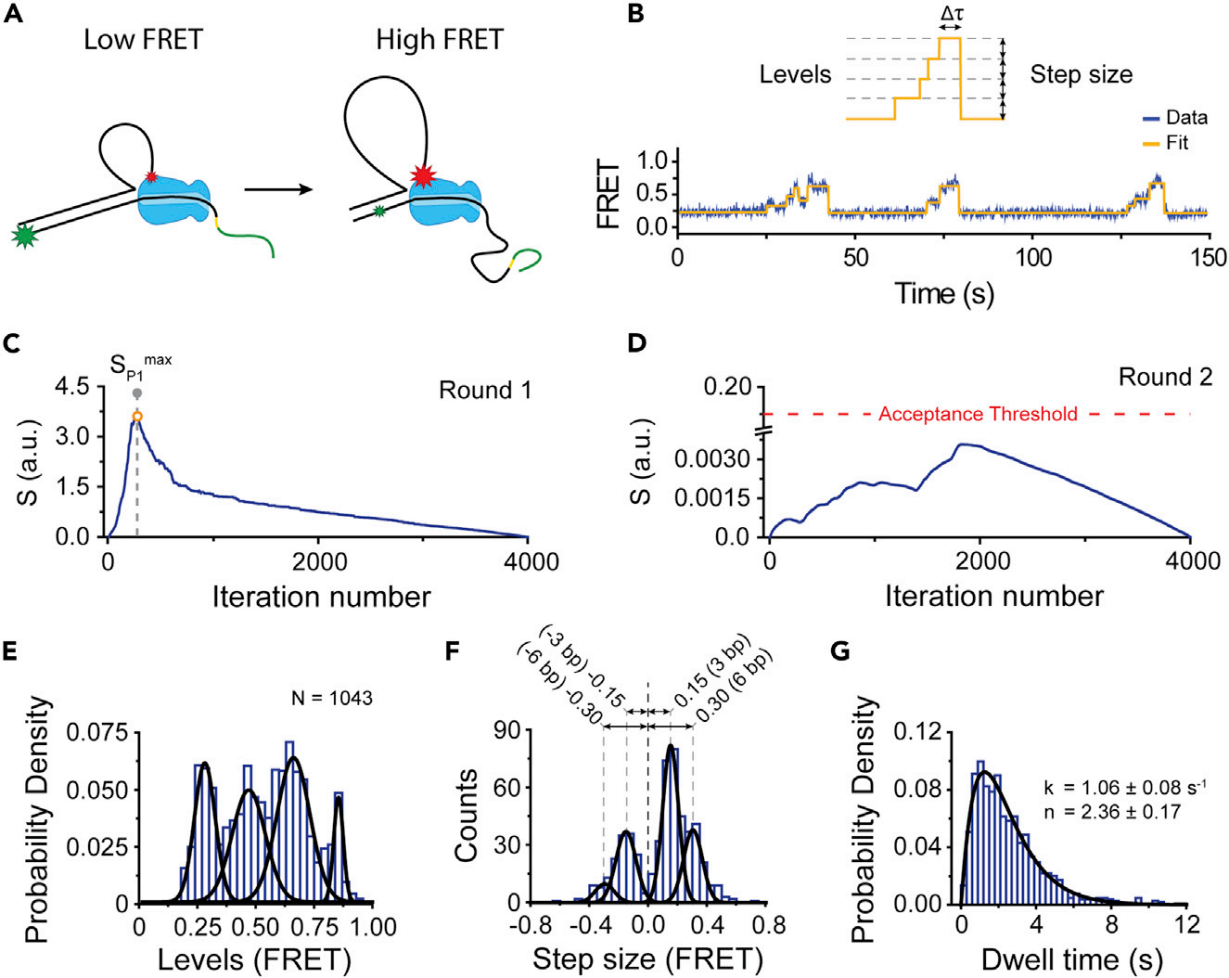
Application of *AutoStepfinder* on experimental FRET data (A) Schematic of loop formation by the CRISPR-associated Cas3 helicase/nuclease protein (blue). The appearance of FRET during loop formation is indicated by the size of the star: low FRET, large green star, or high FRET, large red star. (B) A representative FRET trace (dark blue) fitted with the *AutoStepfinder* algorithm (orange). (C) S-curve for the first round of fitting by *AutoStepfinder*. The dashed gray line indicates the S_P1_^max^ of the S-curve. (D) S-curve for the second round of fitting by *AutoStepfinder*. The global maximum of the S-curve for the second round was below the set acceptance threshold and therefore the second round of fitting was not executed. (E) Distribution of FRET levels obtained through the *AutoStepfinder* algorithm. Black lines represent a Gaussian fit. (F) Distribution of step sizes obtained through the *AutoStepfinder* algorithm. Data were fitted with a gamma distribution (solid line) to obtain the number of hidden steps (n) and rate (k). Error represents the 95% confidence interval obtained through bootstrap analysis. (G) Dwell time distribution obtained through the *AutoStepfinder* algorithm. Black lines represent a gamma distribution. Also see Figure S6.

Execution of the user manual (see supplemental information) described in this paper yields a robust step-detection analysis of single-molecule trajectories (Figure 7). To demonstrate the power of *AutoStepfinder* for one example in more detail, we applied the algorithm on single-molecule fluorescence resonance energy transfer (FRET) trajectories of the CRISPR-associated helicase Cas3. A detailed description on the experimental procedures is described in Loeff et al.^52^ In brief, DNA-bound Cas3 molecules were presented with ATP to unwind DNA. The fluorophores on the DNA substrate reported on DNA unwinding through an increase in FRET (Figure 7A). Before ATP was added, the labeling positions on the DNA yielded a FRET value that was indistinguishable from the background signals. Upon addition of ATP, a stepwise increase in FRET was observed and was analyzed using *AutoStepfinder* (Figure 7B).

The first round of step fitting by *AutoStepfinder* yielded a sharp peak in the S-curve (Figure 7C). In contrast, the second round of step fitting yielded a global maximum below the acceptance threshold and was therefore not executed (Figure 7D). This indicates that *AutoStepfinder* detected steps which had a step size distribution within the same order of magnitude. Next, we used the step *Properties* file (Table S1) to generate histograms of the FRET levels (Figure 7E), step size (Figure 7F), and dwell time (Figure 7G). These histograms show that the helicase moves along the DNA in well-defined steps, resulting in four equally spaced peaks in the FRET level histogram and a dominant peak at 0.15 in the step size histogram. Given that *AutoStepfinder* runs on any signal that exhibits step-like behavior, the algorithm is widely applicable on the trajectories of single-molecule techniques, including force spectroscopy^51^ (Figure S6A–S6C) and nanopore data (Figure S6D–S6F).^59^ Notably, in contrast to the single-molecule FRET data, both the examples of force spectroscopy and nanopore data required dual-pass data fitting (Figures S6B and S6E). Taken together, these analyses show that the *AutoStepfinder* algorithm can detect steps in a wide variety of single-molecule trajectories without any prior knowledge on their size and position.

## Discussion

*AutoStepfinder* is a robust and sensitive first-order step analysis tool that allows step fitting of single-molecule trajectories without any prior knowledge on the step size, step location, and noise contributions within the data. By probing the quality of the fit for every step that is added to the analysis, *AutoStepfinder* provides an assessment of the step fit spectrum (S-curve) within the data. This allows the user to perform a component analysis on the data, where steps at different scales within the data are separated from each other during the analysis. Our benchmark shows that the S-curve provides a robust quality assessment of the steps within the data, displaying a sharp peak when the data are fitted with correct number of steps at each scale.

While the S-curve provides a strong indication of the best solution, users may want to fine-tune the fit by focusing on steps of a particular scale in the data. For example, one may be interested in the large steps in the data, rather than the small steps within each plateau. The user-friendly interface of *AutoStepfinder* provides an environment that allows the user to make an educated decision on which features to fit based on the local maxima within the S-curve. Alternatively, based on the outcome of *AutoStepfinder*, one may design a model-based approach (AIS, SIC) to further fine-tune the fit or use the output of *AutoStepfinder* for machine-learning-based algorithms for high-throughput unsupervised classification and fitting of complex single-molecule trajectories.

Taken together, *AutoStepfinder* facilitates high-throughput step detection with minimal user input within a user-friendly environment that is both robust and sensitive, allowing users to fit experimental single-molecule trajectories without any prior knowledge on the noise and steps within the data. Moreover, given that *AutoStepfinder* is a versatile approach that can be applied on any signal with step-like behavior, we envision that the *AutoStepfinder* algorithm can be used beyond the field of single-molecule biophysics.

## Experimental procedures

### Resource availability

#### Lead contact

Further information and requests for resources should be directed to and will be fulfilled by the lead contact, Cees Dekker (c.dekker@tudelft.nl).

#### Materials availability

There are no physical materials associated with this study.

#### Data and code availability

The AutoStepfinder algorithm can be accessed at Zenodo: https://doi.org/10.5281/zenodo.4657659.

## Supplemental information

Document S1. Figures S1–S12, and Tables S1 and S2, and supplemental experimental procedures

Document S2. Article plus supplemental information

## Footnotes

Supplemental information can be found online at https://doi.org/10.1016/j.patter.2021.100256.
