# Supplementary material for: AutoStepfinder: A fast and automated step detection method for single-molecule analysis
Source: Patterns (N Y). 2021 Apr 30;2(5):100256. doi: 10.1016/j.patter.2021.100256 (PMC8134948; doi:10.1016/j.patter.2021.100256)
Supplement: Document S1. Figures S1–S12, and Tables S1 and S2, and supplemental experimental procedures [file mmc1.pdf]

Patterns, Volume *2*

Supplemental information

AutoStepfinder: A fast and automated step detection method for single-molecule analysis

Luuk Loeff, Jacob W.J. Kerssemakers, Chirlmin Joo, and Cees Dekker

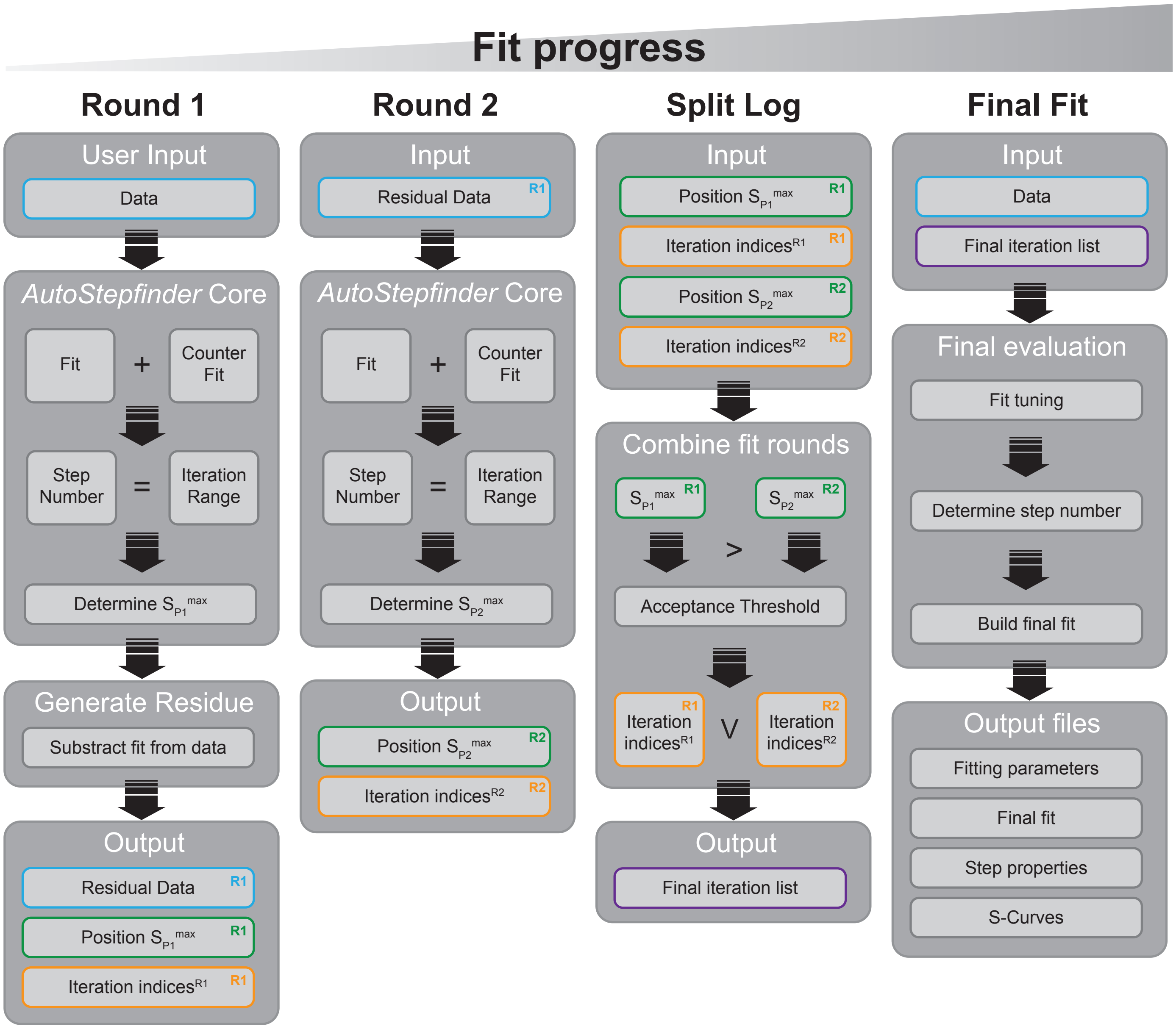

Figure S1: Flow diagram of *AutoStepfinder*

Overall layout of *AutoStepfinder* algorithm. After loading data, the *AutoStepfinder* core executes a series of partition events in which the variance between the fit and the data is minimized. This iterative process of partitioning existing plateaus continues until *AutoStepfinder* executes the maximum number of iterations. After this first round of fitting, *AutoStepfinder* determines the optimal for the data by determining the global maximum of the S-curve (S_{P1}^{max}) and saves the indices of all plateaus in the optimal fit. Subsequently, *AutoStepfinder* subtracts the fit from the data and repeats this step-fitting procedure on the residual data (Round 2). After “Round two”, *AutoStepfinder* enters the “Split Log” stage of the fitting process. In the split log stage *AutoStepfinder* determines if the S_{max} of the first and second round of fitting are above the acceptance threshold and generates a final iteration list. This final iteration list is then used to build the final fit, resulting in multiple output files.

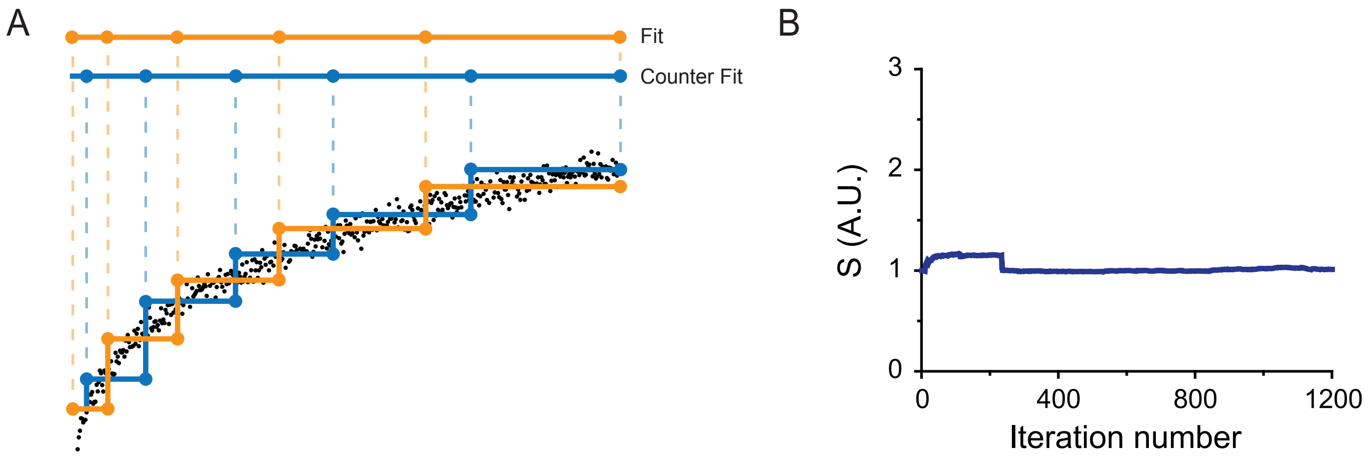

Figure S2: Step-detection in trajectories without steps

(A) Example of a step-fit on a trajectory that does not display step-like behavior. The fit is highlighted in orange line, whereas the additional counter fit is highlighted in blue. Because the data does not display step-like behavior, both the existing fit and counter fit have similar variance. **(B)** A representative example of an S-curve for data that does not exhibit steps. The S value can be calculated by taking the variance of the fit and dividing it by the variance of the counter fit. When the data does not display a step-like behavior, both the existing fit and counter fit have similar variance values, resulting in an S-value close to 1.

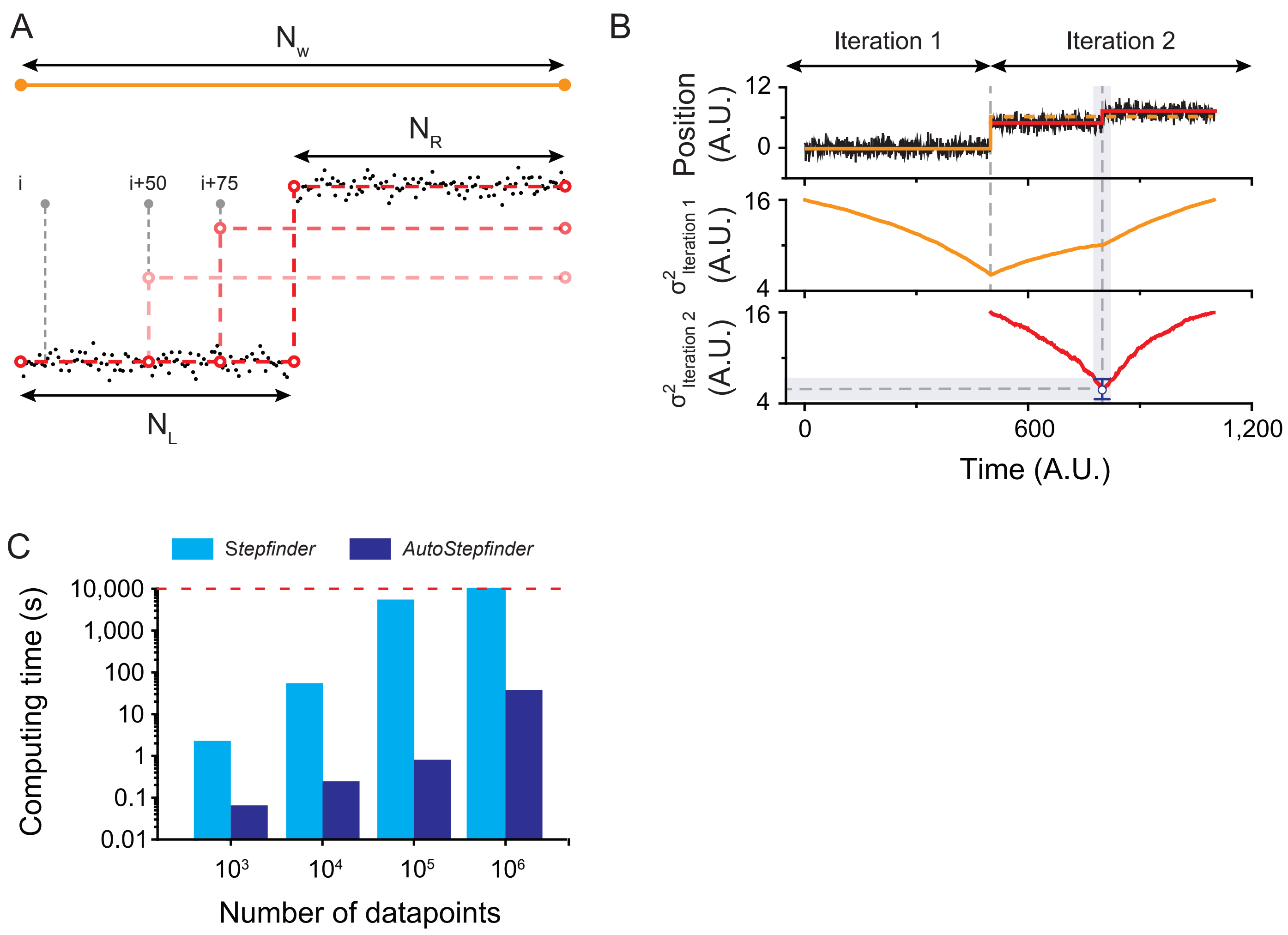

Figure S3: *AutoStepfinder* for step detection in large datasets

(A) An example of the iterative nature of the step fit procedure. The existing plateau (N_W, orange line) is partitioned into two new plateaus (N_L and N_R, dark red dashed line) at a point that yields the largest reduction in the variance. To determine this partition point, the algorithm recalculates the variance for each data point, starting at i until all data points of N_W have been calculated (e.g. i+50, faded red dashed lines). **(B)** Variance landscapes for iterative step-fitting by *AutoStepfinder*. Horizontal and vertical grey shaded areas indicate the bootstrapped step error and time error, respectively. **(C)** Comparison between *Stepfinder*[43] and the *AutoStepfinder* algorithm. The algorithms were tested by measuring the computing time of various datasets on a desktop computer, with default settings of the algorithms. The red dashed line indicates the limit (10,000 sec) that was set for the computing time.

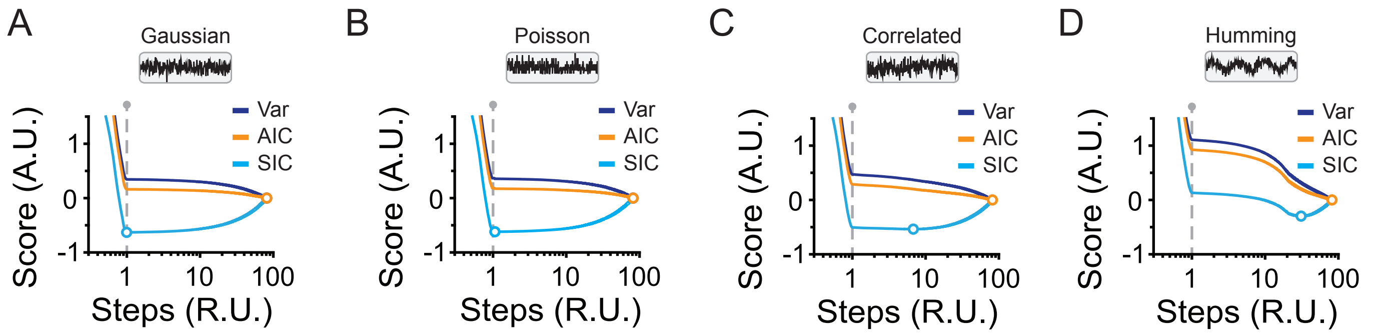

Figure S4: Step-detection with information criteria-based algorithms

(A-D) Step detection on idealized trajectories (see Figure 6a) using the variance (Var), Akaike information criterion (AIC) and Schwarz information criterion (SIC). The idealized trajectories were exposed to distinct noise types: Gaussian noise [a], Poissonian noise [b], correlated noise [c] and humming noise [d]. Each of these trajectories were exposed to noise with a SD=2.0. The dashed grey line indicates the optimal number of steps in the data, normalized at 1.0. The circles indicate the minimum of the respective information criterion.

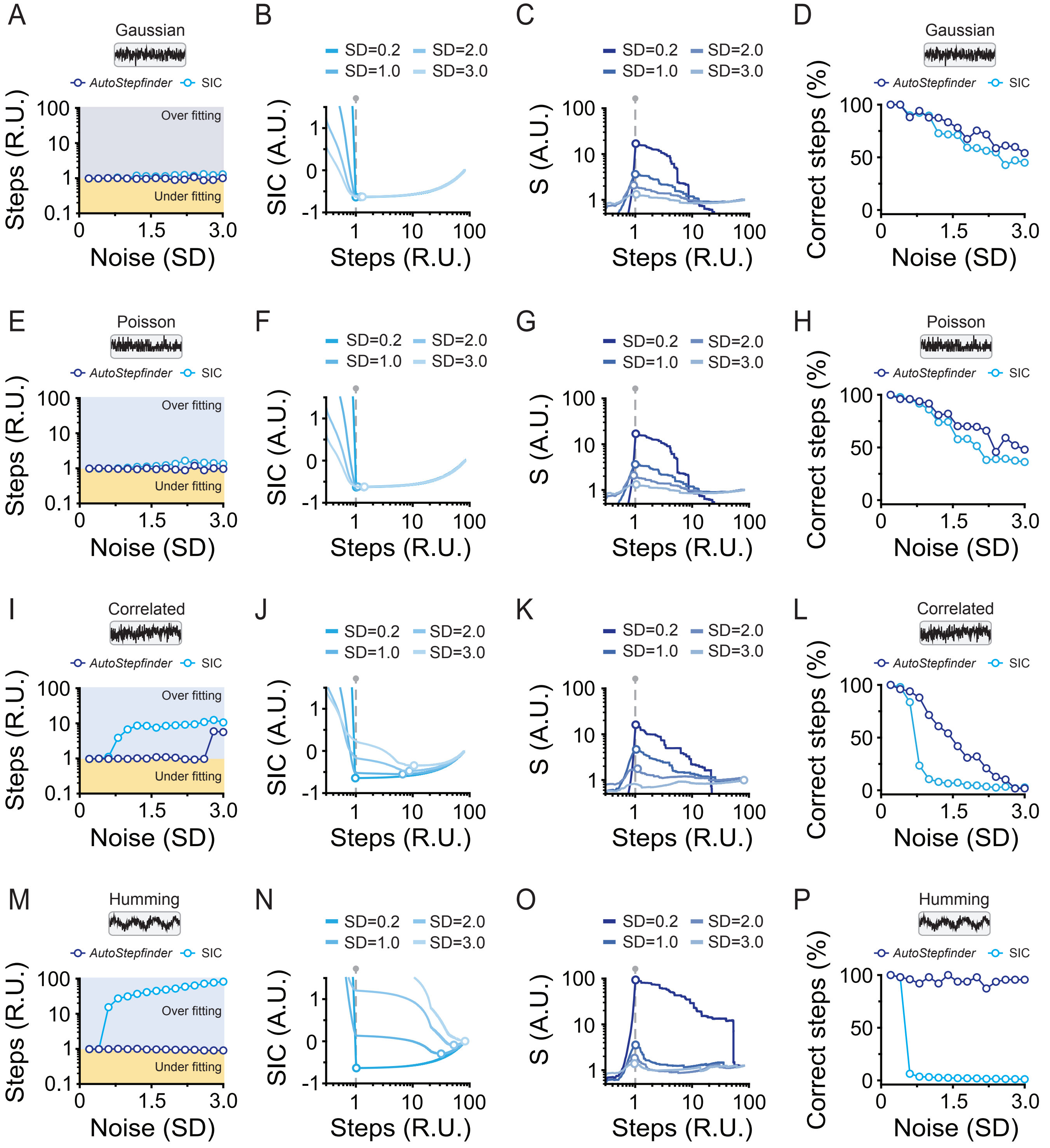

Figure S5: Robustness of *AutoStepfinder* and a SIC based algorithm

(A) Number of steps detected by *AutoStepfinder* and a SIC based algorithm on idealized trajectories (see Figure 6a) exposed to Gaussian noise (inset) with a standard deviation of SD. **(B)** SIC curves of idealized trajectories with Gaussian noise. The dashed grey line indicates the optimal number of steps in the data, normalized at 1.0. The circles indicate the minimum of the SIC curve. **(C)** S-curves of idealized trajectories with Gaussian noise. The dashed grey line indicates the optimal number of steps in the data, normalized at 1.0. The circles indicate the maximum of the S-curve. **(D)** Number of correctly identified steps by the SIC and *AutoStepfinder* algorithm trajectories with Gaussian noise (inset) with a standard deviation of SD. **(E)** Number of steps detected by *AutoStepfinder* and a SIC based algorithm on idealized trajectories (see Figure 2a) exposed to Poissonian noise (inset) with a standard deviation of SD. **(F)** SIC curves of idealized

trajectories exposed Poissonian noise. The dashed grey line indicates the optimal number of steps in the data, normalized at 1.0. The circles indicate the minimum of the SIC curve. **(G)** S-curves of idealized trajectories exposed to Poissonian noise. The dashed grey line indicates the optimal number of steps in the data, normalized at 1.0. The circles indicate the maximum of the S-curve. **(H)** Number of correctly identified steps by the SIC and *AutoStepfinder* algorithm trajectories exposed to Poissonian noise (inset) with a standard deviation of SD. **(I)** Number of steps detected by *AutoStepfinder* and a SIC based algorithm on idealized trajectories (see Figure 4a) exposed to correlated noise (inset) with a standard deviation of SD. **(J)** SIC curves of idealized trajectories with correlated noise. The dashed grey line indicates the optimal number of steps in the data, normalized at 1.0. The circles indicate the minimum of the SIC curve. **(K)** S-curves of idealized trajectories with correlated noise. The dashed grey line indicates the optimal number of steps in the data, normalized at 1.0. The circles indicate the maximum of the S-curve. **(L)** Number of correctly identified steps by the SIC and *AutoStepfinder* algorithm trajectories exposed to correlated noise (inset) with a standard deviation of SD. **(M)** Number of steps detected by *AutoStepfinder* and a SIC based algorithm on idealized trajectories (see Figure 2a) exposed to humming noise (inset) with a standard deviation of SD. **(N)** SIC curves of idealized trajectories with humming noise. The dashed grey line indicates the optimal number of steps in the data, normalized at 1.0. The circles indicate the minimum of the SIC curve. **(O)** S-curves of idealized trajectories with Humming noise. The dashed grey line indicates the optimal number of steps in the data, normalized at 1.0. The circles indicate the maximum of the S-curve. **(P)** Number of correctly identified steps by the SIC and *AutoStepfinder* algorithm trajectories exposed to humming noise (inset) with a standard deviation of SD.

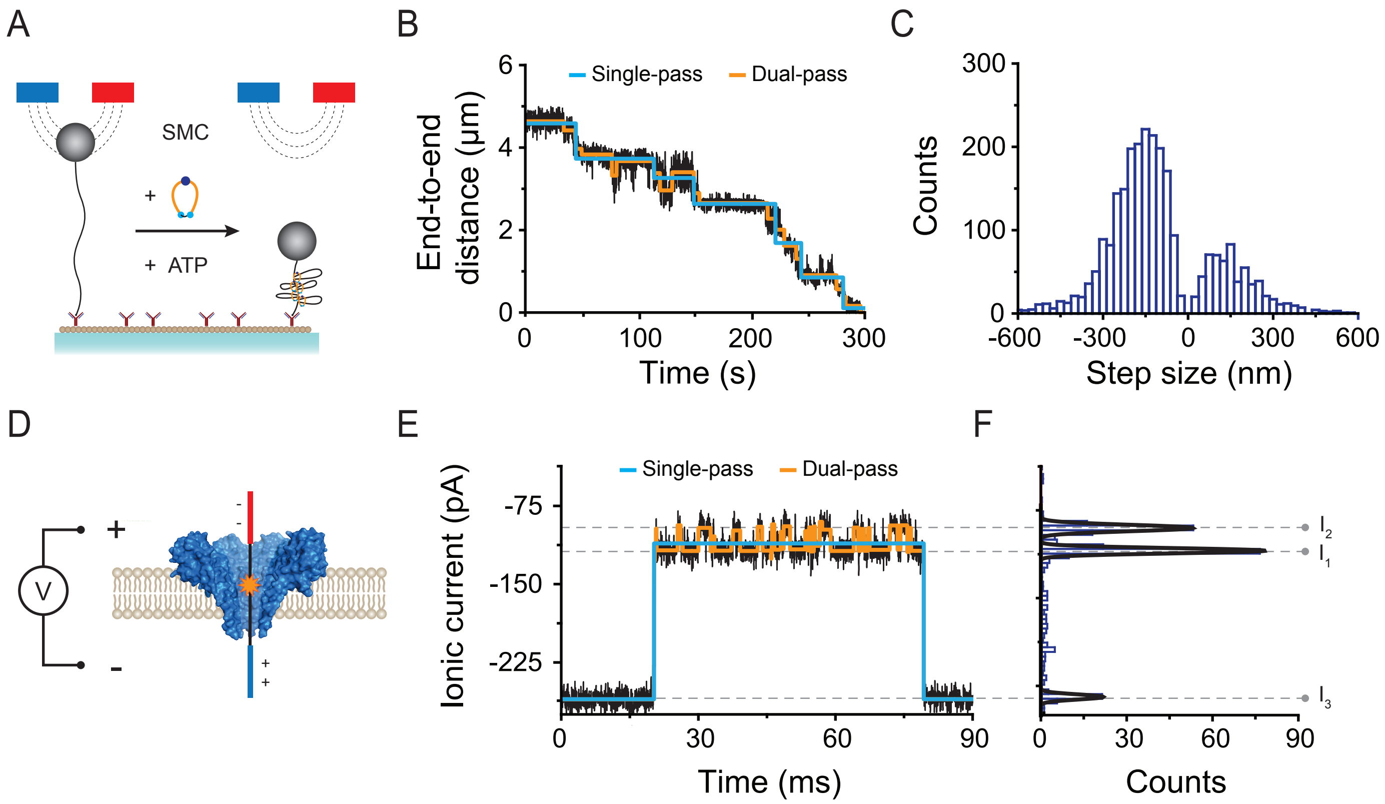

Figure S6: Step detection in magnetic tweezer and nanopore data

(A) Schematic representation of a magnetic tweezer experiment to visualize DNA compaction by condensin (SMC) proteins. A DNA molecule is tethered between a glass slide and a magnetic bead. When condensin and ATP are added, the end-to-end length of the DNA decreases. For a detailed description of these experiments see Eeftens et al.[51]. **(B)** Representative time trajectory displaying step-wise compaction by condensin (black), fitted with the *AutoStepfinder* algorithm over two rounds, single-pass (cyan) dual-pass (orange). **(C)** Distribution of step-sizes in condensin compaction experiments, obtained through the AutoStepfinder algorithm. **(D)** Schematic of a biological nanopore translocating a labelled peptide[59]. **(E)** Representative time trajectory displaying dynamics of a labelled peptide translocating through a biological nanopore (black), ffitted with the *AutoStepfinder* algorithm over two rounds, single-pass (cyan) dual-pass (orange). **(F)** Distribution of blockade levels (I) obtained through the *AutoStepfinder* algorithm. Black lines represent a Gaussian fit.

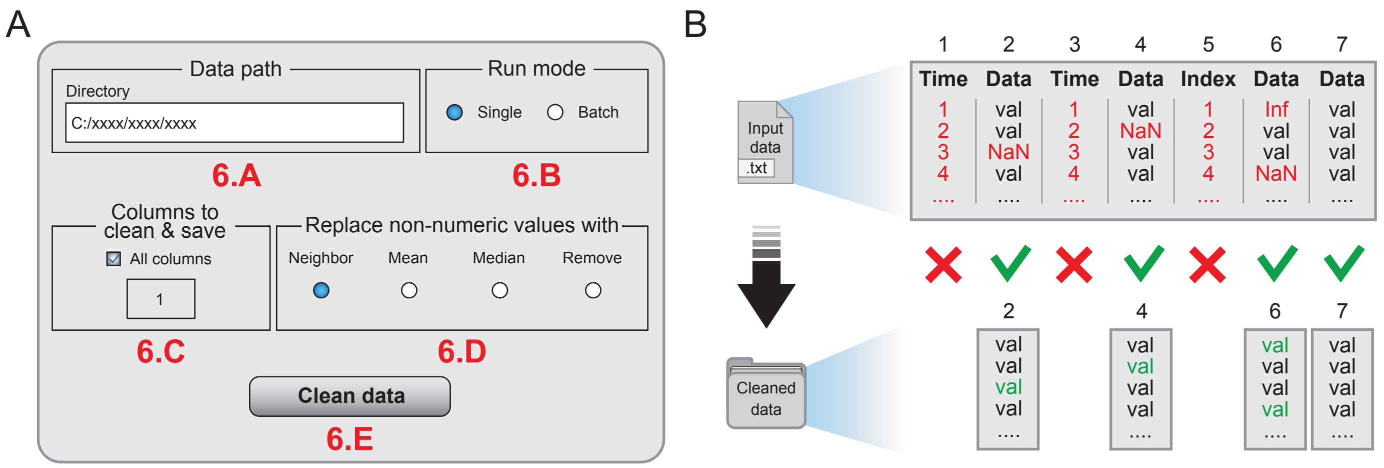

Figure S7: Graphical user interface of *DataDuster*

(A) Schematic of the graphical user interface of *DataDuster*. Red numbers correspond to the steps in the user manual that describe the function of each parameter. **(B)** Workflow of *DataDuster*. When a multi-column .txt file is loaded into *DataDuster*, *DataDuster* will clear each column from NaN and Inf values. Moreover, *DataDuster* will detect and remove the columns that increase uniformly (e.g. time and index access). *DataDuster* will export each column as a separate .txt file that is compatible with *AutoStepfinder*.

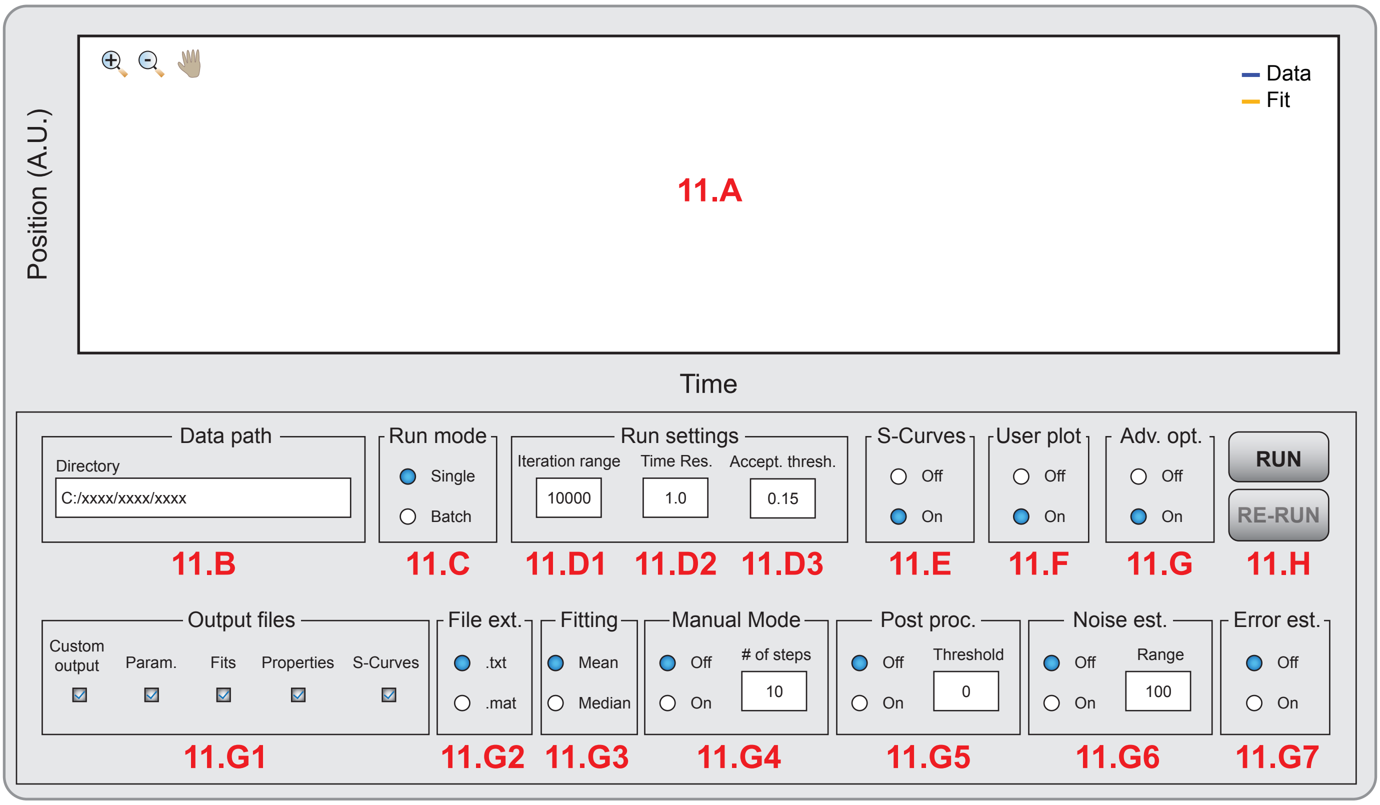

Figure S8: Graphical user interface of *AutoStepfinder*

Schematic of the graphical user interface of AutoStepfinder. The red numbers correspond to the steps in the user manual that describe the function of each parameter.

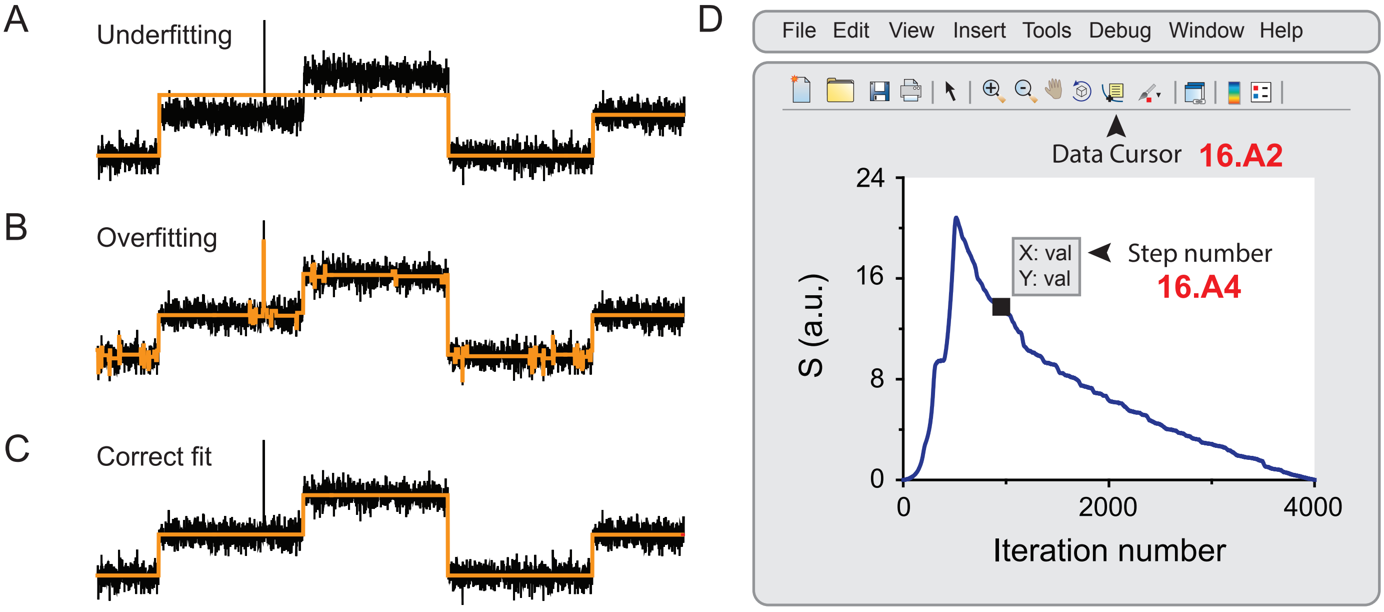

Figure S9: Inspecting the fit of *AutoStepfinder*

(A) Section of an idealized trajectory that is underfitted by *AutoStepfinder*. **(B)** Section of an idealized trajectory that is overfitted by *AutoStepfinder*. **(C)** Section of an idealized trajectory that is correctly fitted by *AutoStepfinder*. **(D)** Schematic of the S-curve window. The data cursor tool can be used to select a feature in the S-curve. The X value represents the step number that can be used in Manual Mode of *AutoStepfinder*. The red number Red numbers correspond to the steps in the user manual that describe the function of each parameter.

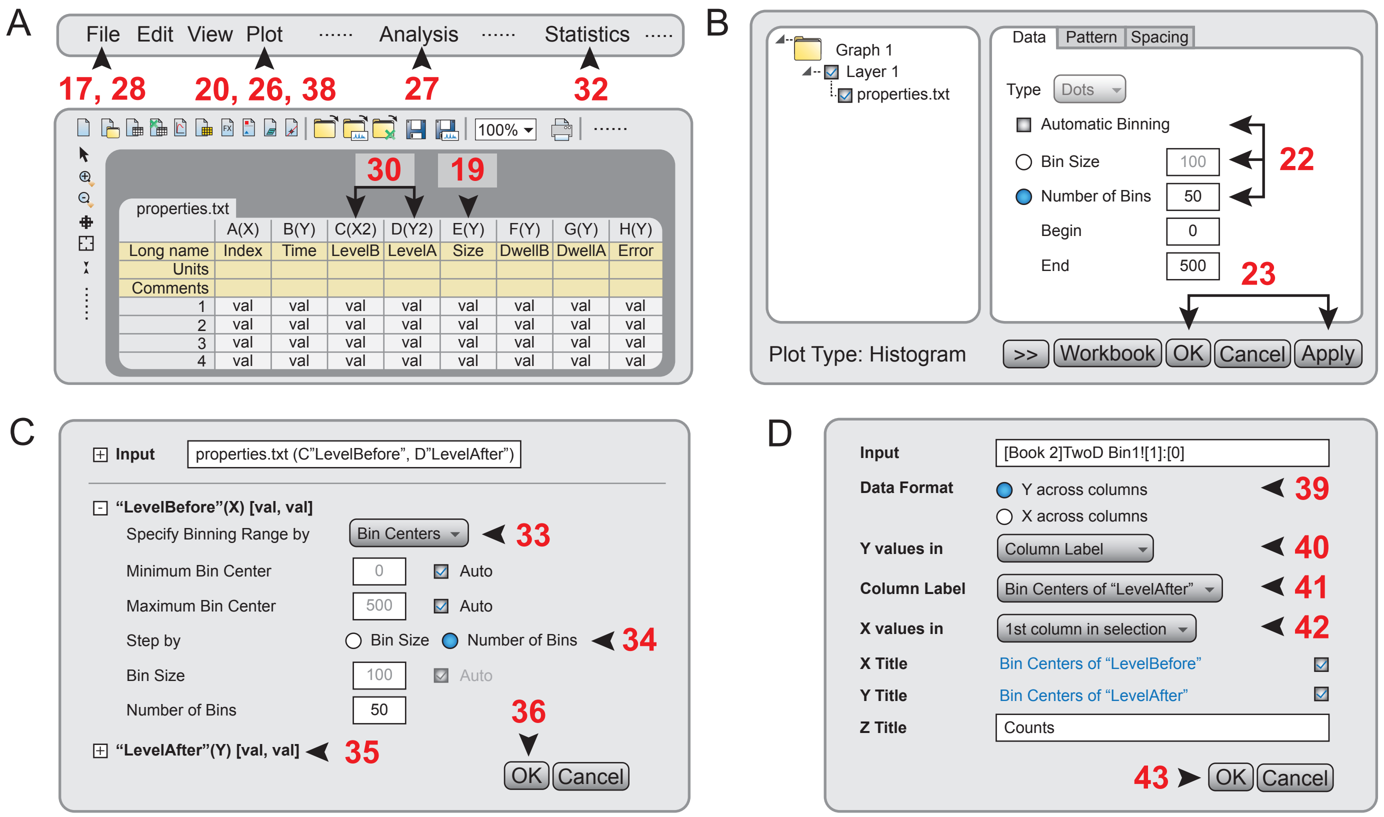

Figure S10: Using the output of *AutoStepfinder* to generate informative plots

(A) Schematic of the main window of OriginPro. Red numbers correspond to the steps in the user manual that describe the function of each parameter. **(B)** Schematic of the histogram binning window in OriginPro. The red numbers correspond to the steps in the user manual that describe the function of each parameter. **(C)** Schematic of the histogram 2D binning window in OriginPro. The red numbers correspond to the steps in the user manual that describe the function of each parameter. **(D)** Schematic of the contour plotting window in OriginPro. The red numbers correspond to the steps in the user manual that describe the function of each parameter.

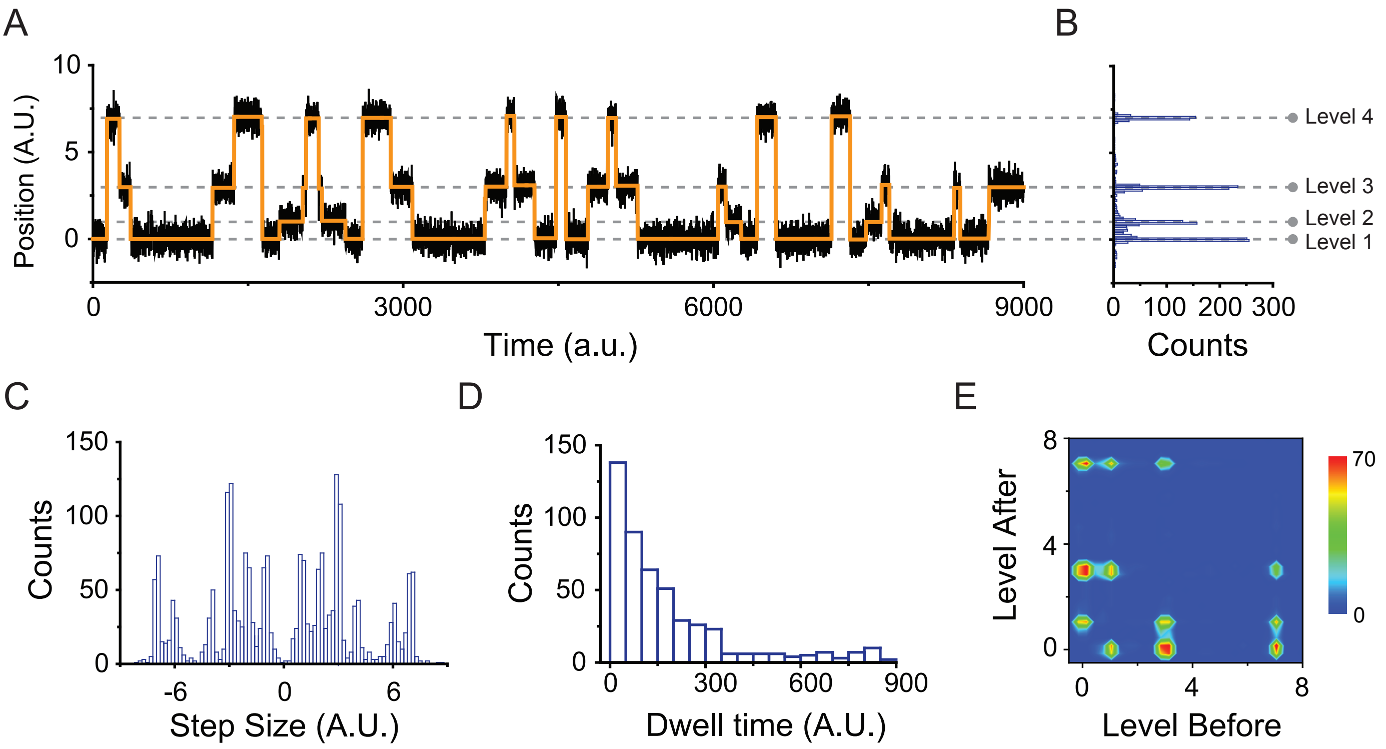

Figure S11: Examples of post-processed *AutoStepfinder* results

(A) Section of an idealized trajectory (black) that is fitted by AutoStepfinder (orange). **(B)** Distribution of levels obtained through the *AutoStepfinder* algorithm. Histogram was obtained by binning and plotting the 'Levels After' column of the properties.txt output file. **(C)** Distribution of step-sizes obtained through the *AutoStepfinder* algorithm. Histogram was obtained by binning and plotting the Step Size column of the properties.txt output file. **(D)** Distribution of dwell times obtained through the *AutoStepfinder* algorithm. Histogram was obtained by binning and plotting the Dwell Time after column of the properties.txt output file. **(E)** Transition density plot obtained through the *AutoStepfinder* algorithm. The Transition Density plot was obtained by 2D binning the Level Before and Level After columns in the properties.txt output file, followed by the generation of a contour plot.

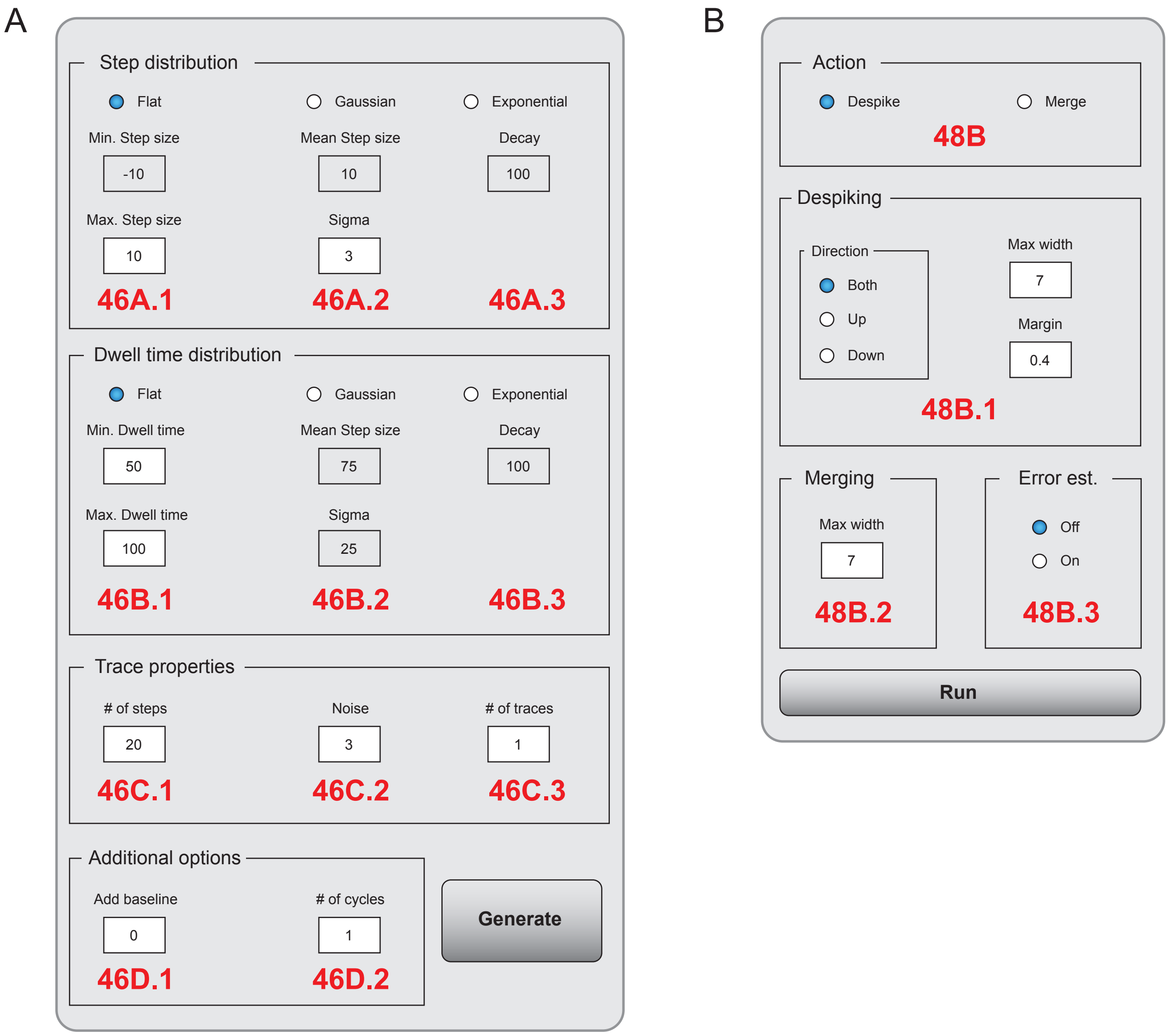

Figure S12: Auxiliary tools in the *AutoStepfinder* package

(A) Schematic of the graphical user interface of *StepMaker*. The red numbers correspond to the steps in the user manual that describe the function of each parameter. **(B)** Schematic of the graphical user interface of *StepMerger*. The red numbers correspond to the steps in the user manual that describe the function of each parameter.

Supplemental Experimental Procedures

Step by step user guide for *AutoStepfinder*

Materials

- A standard PC or Mac suitable for MATLAB with minimum requirements:
    - PC: Windows XP and higher operating system, Processor: any Intel or AMD x86 processor supporting SSE2, Disk space: 2-4 GB, RAM: 2 GB, Graphics: No specific graphics card is recommended.
    - Mac: Mac OS X 10.9.5 or higher operating system, Processor: all Intel-based Macs with an Intel core 2 or later, Disk space: 2-4 GB, RAM: 1 GB, Graphics: No specific graphics card is recommended.
- MathWorks MATLAB version 2015a or above (http://www.mathworks.com)
    - MathWorks MATLAB Database Toolbox
- The *AutoStepfinder* package (the most up-to-date version is available at: http://www.ceesdekkerlab.nl and http://www.chirlmin.org.
- Optional: Data analysis and graphing software to post-process data (e.g. OriginLab Origin (http://www.originlab.com/), Graphpad Prism (http://www.graphpad.com/) or Microsoft Office Exel (http://www.microsoft.com/).

Procedure

General notes on experimental data for optimal step fitting

AutoStepfinder is capable of detecting steps in trajectories of various techniques, including single-molecule fluorescence, nanopores, and magnetic and optical tweezers. While the details of these experimental approaches differ, we provide a set of general guidelines that will maximize the performance of *AutoStepfinder*.

I. **Sampling rate:** *AutoStepfinder* determines the significance of steps based on the number of data points in the plateau (N_i) and the size of the step (Δ) (Figure 2). Thereby, the sampling rate which the single-molecule measurement is performed, i.e. the number of data points per time-unit, is an important factor in step fitting. To facilitate step fitting by *AutoStepfinder*, it is recommended to maximize the number of independent data points per plateau by acquiring data at high sampling rates. *Important:* Increasing the sampling rate in single-molecule measurements may come at a cost. For example, an increased

sampling rate in single-molecule fluorescence measurements will require higher laser powers to collect a large number of photons per frame. These high laser powers will induce fast photobleaching and thereby limit the observation time of the experiment. In addition, a similar upper sampling limit exists for response time-limited systems, such as in magnetic/ optical tweezer and nanopore experiments.

II. **Drift:** A commonly found artefact in single-molecule trajectories is drift or other types of movement in the x-, y- or z-direction. These movements result in trajectories with a gradually decreasing or oscillating signal, which may interfere with the performance of *AutoStepfinder*. Therefore, it is recommended to limit drift during the measurements as much as possible and discard traces from the analysis that show an excessive amount of drift.

III. **Filtering of data:** In single-molecule data analysis, it is a common practice to reduce the noise in the trajectories by smoothing the data with moving averages and filters. However, the use of these filters may also smooth the state-to-state transitions. Given that *AutoStepfinder* works best on instant state-to-state transitions, care should be taken when applying such filters. Therefore, it is advised to run *AutoStepfinder* on raw data or otherwise consider using a step preserving filter, such as a median or Chung-Kennedy filter.

Initializing the *AutoStepfinder* algorithm and auxiliary tools

1. Start MATLAB as described by Mathworks.
2. Copy all the files enclosed in *AutoStepfinder* folder to the working directory of MATLAB. Alternatively, change the initial working directory of MATLAB by going Home tab then Preferences > General > "Initial working folder" and specify the full path to the *AutoStepfinder* folder.

Formatting data for step detection by *AutoStepfinder*

3. *AutoStepfinder* runs on a single-column text file (.txt) that encompasses numeric single-molecule data. Alternatively, *AutoStepfinder* can run two-column text files (.txt) with the time axis in the first column and data in the second column. In the latter case, the time axis will be ignored during the step-fitting procedure.
4. To ensure *AutoStepfinder* runs properly, the input files for *AutoStepfinder* should be free of non-numeric values, including: ‘infinity values’ (Inf) and ‘not a number’ (NaN).

To remove these values, one could use the *DataDuster* auxiliary tool, which is located in the *AutoStepfinder* package.

5. Start the *DataDuster* auxiliary tool by opening DataDuster.m and running the code in the editor tab or the command window of MATLAB.
6. After pressing “Run”, a graphical user interface (GUI) will appear, in which the user can adjust the run settings for *DataDuster* (Figure S7).

   For troubleshooting, see table S2

   6A. *Data Path*: The Data Path box allows one to specify the directory of the input data. <u>*Important:*</u> By default, the directory is set to the current directory of MATLAB. The default location of Data Path can be changed at line 47 of the DataDuster.m file.

   6B. *Run Mode*: Run mode specifies whether one runs *DataDuster* on a single file or run all files in a selected folder. To run a single .txt file, check single and run the algorithm to select a file. For batch style processing, check batch and *DataDuster* will analyze all .txt files in the specified directory.

   6C. *Columns to clean and save*: The columns to clean and save box allows one to specify on which column to run *DataDuster.* By default, it runs through all columns in the loaded .txt file(s). To specify a specific column, uncheck the “all columns” box and specify the number of the column to analyze.

   6D. *Replace non-numeric values with*: The replace non-numeric values with box allows one to specify what to do with the non-numeric values.

   6.D1. *Neighbor*: Replaces non-numeric values with the mean value of the neighboring two data points.

   6.D2. *Mean*: Replaces non-numeric values with the mean value of the dataset.

   6.D3. *Median*: Replaces non-numeric values with the median value of the dataset.

   6.D4. *Remove*: Removes non-numeric values from the dataset.

   6E. *Clean data:* The clean data button initiates *DataDuster* data cleaning procedure.
7. To start the data cleaning procedure, press the “Clean data” button, located on the bottom of the GUI (Figure S7A).
8. Browse to the directory of interest and select the file (single run) or the folder (batch run) that encompasses the single-molecule data for data cleaning.

9. The output of the *DataDuster* is saved in a new folder called "cleaned_data_method", where method refers to the input of the "replace non-numeric values with" box. This folder is generated in the directory of the input file. If a multi-column .txt file was loaded, *DataDuster* will output each column as a separate .txt file named: filename_col_0x.txt, which can be directly loaded into the *AutoStepfinder* algorithm. Notably, the MATLAB console will display the number of replaced values in each column. *Important:* *DataDuster* does not export trajectories that exhibit equidistant increase in signal, e.g. the time axis of trajectories or indices, as these trajectories are featureless and will not result in step detection by the *AutoStepfinder* algorithm.

Startup and Graphical user interface of *AutoStepfinder*

10. Start *AutoStepfinder* by opening AutoStepfinder.m and running the code in the editor tab or the command window of MATLAB.
11. After pressing "Run", a GUI will appear, in which the fitting procedure will be executed and fitting parameters can be adjusted (Figure S8).

    11A. *Fitting Window*: The top half of the GUI comprises a fitting window. This window allows one to visually inspect the fit after executing the *AutoStepfinder* algorithm. The data is displayed in blue and the corresponding fit in orange.

    11B. *Data Path*: The Data Path box allows one to specify the directory of the input data. *Important:* By default, the directory is set to the current directory of MATLAB. The default location of Data Path can be changed at line 38 of the AutoStepfinder.m file.

    11C. *Run Mode*: Run mode specifies whether one runs *AutoStepfinder* on a single file or run all files in a selected folder. To run a single .txt file, check single and run the algorithm to select a file. For batch style processing, check batch and *AutoStepfinder* will analyze all .txt files in the specified directory.

    11D. *Run Settings*: The Run Settings box provides a minimal set fitting of parameters that allows one to tune the fitting procedure (Figure S8).

        11.D1. *Iteration range*: The iteration range parameter determines to what extent *AutoStepfinder* continues the fitting procedure. Once the number defined by the

iteration range is found, the algorithm stops partitioning plateaus to minimize χ^2 and determines the optimal fit. For datasets with limited step numbers, the iteration range parameter can be decreased to reduce the computing time of the fitting procedure. Typically, the initial iteration range is set to ¼ of the number of data points of the input data.

11.D2. *Time resolution*: The time resolution parameter corresponds to the temporal resolution (e.g. the time interval between each data point) of the measurement. This parameter will be used for the time data in the output files of *AutoStepfinder*. Important: If a file with two columns is provided, time and data, the time column (first column) is ignored by the *AutoStepfinder* algorithm.

11.D3. *Accept(ance) thresh(old)*: The acceptance threshold sets a threshold for each fitting round and is compared to S^{max}-1. If the S-curve of the first or second fitting round provides a S^{max} that lays below the threshold, this fitting round will not be executed. Typically, the acceptance threshold ranges between: 0.1 – 1. *Important:* Care should be taken when adjusting the acceptance threshold. If the acceptance threshold is set above the S^{max} of the first round of fitting, *AutoStepfinder* will not execute the step-finding procedure.

11E. *S-Curves*: When S-Curves are turned on, the *AutoStepfinder* will display the S-curves of the first and second round in a separate window.

11F. *User plot*: The user plot box allows one to quickly assess the fitting result of the *AutoStepfinder* algorithm. By turning the *User plot* function on, *AutoStepfinder* will plot the step size, step levels and dwell-time histograms in a separate window.

11G. *Adv(anced) Options*: Enabling the advanced option box displays the advanced setting of *AutoStepfinder* (Figure S8). *Important:* It is noted that these settings are intended for advanced users that have full understanding on the step fitting procedure.

11.G1. *Output files:* The output files box allows one to select which output files *AutoStepfinder* saves. By saving a subset of output parameters, additional speed can be gained, which may be preferred for large datasets or when optimizing fitting settings.

11.G2. *File ext(ension)*: The file extension box allows one to select the file type *AutoStepfinder* outputs. When .txt is checked, *AutoStepfinder* outputs text files. If .mat is checked, *AutoStepfinder* outputs matlab files, which can be used to further post-process the output of *AutoStepfinder* in Matlab.

11.G3. *Fitting*: The fitting box allows one to choose how the position of the plateaus of the final fit is determined. By default, *AutoStepfinder* uses the averages of each plateau to determine its position for each iteration and the final fit. However, in some cases (e.g. when data exhibits spikes), one may choose to build the final fit using the median of each plateau, by checking the median parameter.

11.G4. *Manual mode*: Manual mode allows one to define the number of steps that have to be found by the algorithm. Typically, the number of manually fitted steps does not exceed the iteration range. *Important:* Manual mode overrides the quality assessment of *AutoStepfinder* and should only be used in an informed manner.

11.G5. *Post proc(essing)*: The post processing box allows one to discard the baseline plateaus from the fit. All fitted plateaus that have a value below the provided threshold in units of the input data are removed from the "filename_properties" output file.

11.G6. *Noise estimation*: The noise estimation box allows the user to perform pairwise distance noise estimation on the residual noise in the data (data with the fit substracted). By default, the range over which the noise estimation is performed is set to 100 data points. By enabling the *Noise estimation* function, AutoStepfinder opens a new window with curves that correspond to the Residual noise (blue line), the median (red line) and the pairwise distance noise (red circle). *Important:* For low pass filtered data the pairwise distance error may be under estimated, in this case it is advised the median value as noise estimate.

11.G7. *Error estimation*: The error estimation box allows the user to perform bootstrap analysis that provides the 95% confidence intervals of both the step-sizes and the plateaus. When error estimation is enabled, the *AutoStepfinder* output includes two additional columns with the respective 95% confidence intervals.

11H. *Run and Re-run*: The run button initiates *AutoStepfinder* step fitting procedure. The Re-run button enables after a single run has been executed and allows the user to re-analyze the data without having to load the data.

Running *AutoStepfinder*

12. To start the step-finding procedure press the "Run" button, located on the right side of the GUI (Figure S8).
13. Browse to the directory of interest and select the file (single run) or the folder (batch run) that encompasses the single-molecule data for *AutoStepfinder*.
14. Press open to start the step-finding procedure. The progress of the *AutoStepfinder* analysis is displayed in the console of MATLAB. Once the console indicates "done!" the fitting procedure has been completed and output files have been saved.
15. The output of the *AutoStepfinder* analysis is saved in a new folder (StepFit_Result), which is generated in the directory that is provided in the datapath box of the GUI. By default, the output of *AutoStepfinder* consists of four files: "filename_fits", "filename_properties" and "filename_s_curve" and "filename_config". The "filename_fits" file consists of three columns (Table S1) and can be used to plot the data with corresponding fit. The "filename_properties" file consists of 8 columns (Table S1) and encompasses the information required to generate histograms of the step size, step levels and dwell-times. The "filename_s_curve" file encompasses the information required to plot the S-curves of the first and second round (Table S1). Lastly, *AutoStepfinder* generates a "filename_config" file that encompasses all the parameters that were used to generate the fit (Table S1). Notably, when batch mode is selected, *AutoStepfinder* generates additional .JPEG files of the fit window, user plots and S-curves (Table S1).

Fine tuning the fit parameters for optimal results

16. <u>*Important:*</u> *AutoStepfinder* is a robust approach for automated step detection that determines the optimal fit based on statistical arguments. However, despite the automated detection of steps, it is advised to always carefully inspect the quality of the

fitting result before proceeding with post-processing of the data. The quality of the fit can be assessed by using the fitting window and the built-in controls of the GUI (e.g. zoom in/out and pan) (Figure S8, Step 11.A). Below we provide guidelines on how to interpret and fine-tune fitting parameters to obtain optimal fitting results. As a rule of thumb, it is recommended to maintain a conservative attitude towards step fitting in which it is better to miss small events rather than to introduce spurious steps by overfitting.

16A. *Underfitted data:* Data is considered underfitted when the number of detected steps by *AutoStepfinder* is significantly lower than the number of steps that are present in the data. Therefore, a hallmark for underfitted data is a fit in which a significant number of steps are missed. At the location where steps are obviously missed, the plateau of the fit deviates from the data (Figure S9A) and thereby these plateaus are generally associated with in large step errors (properties output file, column 8). Underfitting of data is typically associated with irregular features in the S-curve; therefore, it is recommended to inspect the corresponding S-curve (11.E). While the S-curve normally shows a sharp peak at the optimal step number, for some datasets the S-curve may have a non-canonical shape. For example, it might have a secondary peak or shoulder that represents a more realistic step number to fit.

Typically, underfitting can be prevented by adjusting the parameterization of the *AutoStepfinder* algorithm. Underfitting may occur when the final number of steps in the data is too close to the user provided iteration range (Step 11.D1) or when the S^{max} of the second round of fitting lies below the acceptance threshold (Step 11.D1). Thereby, underfitting can be prevented by increasing the iteration range or by lowering the acceptance threshold. Alternatively, one can determine the position of a specific feature in the S-curve (e.g. a shoulder or secondary peak) as follows:

16.A1. Select the data cursor tool from the build in controls of the S-curve plotting window (Figure S9D)

16.A2. Use the data cursor tool to determine the step number (X value) at which the shoulder or secondary peak in the S-curve occurs (Figure S9D).

16.A3. Enable to advanced settings and engage manual mode (Step 11.G4) under the advanced settings (Step 11.G).

16.A4. Insert step number that was determined with the data cursor tool in the manual mode box (Figure S9D).

16.A5. Run *AutoStepfinder* with manual mode engaged.

Important: By engaging manual mode *AutoStepfinder* fits the user-defined number of steps to the data, bypassing the quality assessment of the *AutoStepfinder* algorithm. Therefore, the use of manual mode should always be guided by specific features of the S-curve. It is strongly discouraged to use manual mode without a compelling rationale.

16B. *Overfitted data:* Data is considered overfitted when the number of detected steps by *AutoStepfinder* is significantly higher than the number of steps that are present in the data. Therefore, a hallmark for overfitted data is a fit in which plateaus are fitted with a significant number of small steps that follow the noise of the data (Figure S9B). By fitting the noise of the data, plateaus are divided into smaller ones, which can detrimental for the outcome of the step analysis (e.g. dwell-times can be significantly shorter when data is overfitted). In most experimental contexts, it is better to miss small events than to introduce spurious small steps by overfitting.

Overfitting of data is typically associated with wrong parameterization of the *AutoStepfinder* algorithm. Overfitting of data by *AutoStepfinder* typically occurs when the user-defined acceptance threshold is set too low. As a result of the low acceptance threshold, *AutoStepfinder* will consider noise as small steps and overfit the data (Step 11.D3). In some cases, overfitting may occur when the user provides an iteration range that is approximately more than an order of magnitude larger than the number of steps in the data, which can be prevented by lowering the iteration range (Step 11.D1).

16C. *Correctly fitted data:* A fit describes the data well when the majority of the plateaus are fitted, while noise and other artefacts in the data are not included in the fit (Figure S9C). If one is satisfied with the fitting results proceed to step 17 of this protocol.

Post-processing of *AutoStepfinder* output

The output of *AutoStepfinder* can be post-processed to generate informative plots using any kind of spreadsheet or graphing software (e.g. OriginPro, Prism, SigmaPlot, MATLAB, Python and Excel). Below we provide a description on how the data can be processed using OriginPro.

Step-size, level and dwell-time histograms

17. Open OriginPro and load the filename_properties.txt file by going to File > Import and select Single ASCII (Figure S10A).

18. Select the filename_properties.txt in the StepFit_Result folder and click “Open”.
19. Select a column of interest (e.g. column 5, StepSize) by clicking on the column header (E(Y)). The column should now be highlighted in black (Figure S10A).
20. To generate a histogram, go to Plot > Statistics and select “Histogram” (Figure S10A).
21. Double clicking on the bars of the histogram will open the Plot Details window (Figure S10B) that allows one to tune the bin size. Alternatively, right click on the bars of the histograms and select “Plot Details”.
22. Uncheck “Automatic Binning”, and define a bin size or a number of bins by selecting “Bin Size” or “Number of Bins”, respectively (Figure S10B). As a rule of thumb, one can estimate the appropriate number of bins for a dataset by taking the square root of the number of data points in the dataset (round off if necessary).
23. Once the appropriate number of bins has been determined press “Apply” and “OK” (Figure S10B). This will generate a histogram of the selected column, for example with Levels (Figure S11B), Step size (Figure S11C) or Dwell-times (Figure S11D). Notably, for step size histograms a peak at a negative step size indicates a step from a higher level to a lower level, whereas a peak at a positive step size indicates a step from a lower to a higher level (Figure S11C).
24. To fit the histograms, the histograms need to be converted to a bar plot. To convert the histogram to a bar plot, right click on the histogram and select “Go to bin worksheet”.
25. Select the “Bin Centers (X)” and “Counts (Y)” columns.
26. With the columns selected go to Plot > Column/ Bar/ Pie and select “Column” (Figure S10A).
27. This bar plot can be fitted with different functions, depending on the distribution of the data. For example, normally distributed data can be fitted with a Gauss function by going to Analysis > Peaks and Baseline > Multiple Peak Fit and selecting: “Open Dialog”, whereas data that follows an exponential decay can be fitted by going to Analysis > Fitting > Exponential Fit and selecting: “Open Dialog” (Figure S10A).

Transition density plots

28. Open OriginPro and load the filename_properties.txt file by going to File > Import and select Single ASCII (Figure S10A).
29. Select the level before (C(Y)) column.

30. Right click on the selected column and click on: Set As > X, the column header should change from C(Y) to C(X2) (Figure S10A).
31. Select the level before (C(X2)) and level after (D(Y2)) column by clicking on the column header. The column should now be highlighted in black.
32. Bin the data in 2D by going to Statistics > Descriptive Statistics > 2D Frequency Count/ Binning and select "Open Dialog" (Figure S10A).
33. Adjust "Specify Binning Range by" to "Bin Centers" (Figure S10C).
34. Uncheck "Automatic Binning", and define a bin size or a number of bins by selecting "Bin Size" or "Number of Bins" (Figure S10C). As a rule of thumb, one can estimate the appropriate number of bins for a dataset by taking the square root of the number of data points in the dataset, round off if necessary.
35. Repeat step 33-34 for the Y data, selecting the same parameters, such as bin size/ bin numbers (Figure S10C).
36. Press "OK" to generate a new workbook with 2D binned data (Figure S10C).
37. Select all columns of the newly generated workbook with 2D binned data.
38. With the columns selected go to Plot > Contour and select "Color Fill" (Figure S10A).
39. In the pop-up window select "Y across columns" for "Data Format" (Figure S10D).
40. Change "Y Values in" to "Column Label" (Figure S10D).
41. Make sure that in the "Bin Centers", "LevelAfter" is selected under "Column Label" (Figure S10D).
42. Select "1st column in selection" for "X values in" (Figure S10D).
43. Press "OK" (Figure S10D) and the transition density plot will be generated (Figure S11E).
44. The contour plot can be formatted by right clicking on the center of the graph window and going to "Plot Details".

Generating trajectories with StepMaker

StepMaker is a tool that allows a user to generate trajectories of various techniques, including single-molecule fluorescence, nanopores, and magnetic and optical tweezers. Below we provide a set of general guidelines that demonstrate how *StepMaker* can be tuned to obtain specific trajectories.

Startup and Graphical user interface of *StepMaker*

45. Start *StepMaker* by opening StepMaker.m and running the code in the editor tab or the command window of MATLAB.
46. After pressing “Run”, a GUI will appear, in which the simulation procedure will be executed and parameters can be adjusted (Figure S12A).

    46A. The step distribution box allows one to select how steps are distributed in the simulated trajectory (Figure S12A). The user has the choice between:

    46A.1 *Flat distribution*: When the step distribution is flat all step sizes between the indicated minimum and maximum step size have an equal chance of occurring.

    46A.2 *Gaussian distribution*: When the step distribution is gaussian step sizes are randomly picked from a gaussian distribution with the indicated mean and sigma.

    46A.3 *Exponential distribution*: When the step distribution is exponential, steps are randomly picked from an exponential distribution with the indicated decay constant.

    46B. The dwell time distribution box allows one to select how steps are distributed in the simulated trajectory. The user has the choice between:

    46B.1 *Flat distribution*: When the dwell time distribution is flat all dwell times between the indicated minimum and maximum dwell times have an equal chance of occurring.

    46B.2 *Gaussian distribution*: When the dwell time distribution is gaussian dwell times are randomly picked from a gaussian distribution with the indicated mean and sigma.

    46B.3 *Exponential distribution*: When the dwell time distribution is exponential, dwell times are randomly picked from an exponential distribution with the indicated decay constant.

46C. The trace properties box (Figure S11A) allows the user to select the properties of the simulated trajectory:

46C.1 *# of steps*: Number of steps in the generated trajectory. Naturally, the number of dwells is one unit higher.

46C.2 *Noise*: Standard deviation of the Gaussian noise in the signal.

46D.2 *# of Traces*: Number of trajectories that are generated by *StepMaker*.

46D. Additional options (Figure S12A):

46D.1 *Add baseline*: Number of data points to be added before or after the trajectory. When the value of the baseline is negative the baseline is added to the beginning to the trajectory. When the value of the baseline is positive the baseline is added to the beginning to the trajectory.

46D.2 *# of cycles*: Number of repeats within the trajectories that are generated by *StepMaker*. For example, this option can be used to make two-state transition trajectories.

Postprocessing trajectories with StepMerger

StepMerger is a tool that allows a user to remove statistically significant features that may not of interest, such as blinking and spikes from the output of *AutoStepfinder*. Below we provide a set of general guidelines that demonstrate how *StepMerger* can be tuned to remove these features.

Startup and Graphical user interface of *StepMerger*.

47. Start *StepMerger* by opening StepMerger.m and running the code in the editor tab or the command window of MATLAB.
48. After pressing "Run", a GUI will appear, in which the merging procedure will be executed and parameters can be adjusted (Figure S12B).
    48A *Input directory:* The Input directory box (Figure S12B) allows one to specify the directory of the input data. *Important:* By default, the directory is set to the current directory of MATLAB.

    48B. *Action:* The action box allows the user to choose how the data is processed.

48B.1 *Despiking:* Despiking allows to the user to remove blinks and spikes that return to the same level from the *AutoStepfinder* output. Spikes and blinks that are within the indicated maximum width will be removed. Moreover, the margin option determines the maximum relative difference between the up and down steps that comprises the spike.

48B.2 *Merging:* Merging allows the user to merge small spurious steps that are within the indicated max width and that move after another in the same direction. These small spurious steps are typically associated with non-instantaneous steps.

48B.3 *Error estimation*: The error estimation box allows the user to perform bootstrap analysis that provides the 95% confidence intervals of both the step-sizes and the plateaus. When error estimation is enabled, the *StepMerger* output includes two additional columns with the respective 95% confidence intervals.

49. The output of the *StepMerger* has the same format as *AutoStepfinder* (the fit and properties), which are generated in the directory that is provided in the input directory box of the GUI.

Table S1| Output of the *AutoStepfinder* algorithm

File	Column	Name	Description
filename_fits	1	Time	Time axis of the dataset. *Important:* If the time resolution was not provided in the Run Settings box, the time axis is converted to indices.
	2	Data	Data that has been loaded into *AutoStepfinder*.
	3	Fit	The corresponding fit of the data that was generated by *AutoStepfinder*.
filename_properties	1	Index Step	Index based location of the step between two plateaus. The location of the step is defined by the last data point of the plateau that is located on the left.
	2	Time Step	Time based location of the step between two plateaus. *Important:* Notably, if the time resolution was not provided in the Run Settings box, the time is converted to indices.
	3	Level Before	Level of the plateau before the step occurred.
	4	Level After	Level of the plateau after the step occurred.
	5	Step Size	Signal difference between the two plateaus. Notably, a negative step size indicates a step from a higher level to a lower level, whereas a positive step size indicates a step from a lower to a higher level.
	6	Dwell Time Step Before	Dwell time of the plateau before the step occurred.
	7	Dwell Time Step After	Dwell time of the plateau after the step occurred.

	8	Error	Predicted error of the step size, which is based on the plateau length and step size.
	9	Bootstrap error of the step size	The 95% confidence interval of the step size determined by bootstrap analysis
	10	Bootstrap error of the time	The 95% confidence interval of the time determined by bootstrap analysis
filename_SCurve	1	Step Number	The number of steps that have been fitted to the data.
	2	SCurve Round 1	S-values of the first round of fitting.
	3	SCurve Round 2	S-values of the second round of fitting.
filename_config	-	-	A list of all the fitting parameters that were used by *AutoStepfinder*.
filename_fitfig (exclusive for batch analysis)	-	-	An .JPEG image of the fitting window (11.A), showing the raw data and fit.
filename_s_curve (exclusive for batch analysis)	-	-	An .JPEG image of the s-curve window (11.E3), showing S-curves of round 1 and 2.
filename_user_plot (exclusive for batch analysis)	-	-	An .JPEG image of the userplot window (11.G), showing the plots of step-size and step-levels.

Table S2: Troubleshooting *AutoStepfinder*

Step	Problem	Possible reason	Solution
6A	Pop-up with Error: The provided directory is not valid.	The provided directory does not exist.	Provide an existing data path.
		The provided directory is not a folder.	Provide a data path to the file directory.
6C	Popup with Error: The input for the number of columns is NaN.	The input for the number of columns is not a number (NaN).	Provide a number as input for the number of columns.
7	Popup with Error: The provided input folder is empty.	The provided input folder is empty.	Select a folder that contains .txt files with your data.
	Pop-up with Error: 'FileName' contains is not formatted properly.	The data may be the wrong filetype.	Check the file extension of the input data. The file extension should be .txt.
		The data may contain characters	Alternatively, check if the input data contains characters.
10	The *AutoStepfinder* GUI does is not displayed as in Figure 8.	Screen resolution is too low.	Increase screen resolution. Alternatively, resize GUI window.
		Monitor size is smaller than 17".	Connect a larger monitor to your computer. Alternatively, resize GUI window.
11.B	Pop-up with Error: The provided directory is not valid.	The provided directory does not exist.	Provide an existing data path.
		The provided directory is not a folder.	Provide a data path to the file directory.
11.C	Popup with Error: Empty folder.	The provided input folder is empty.	Select a folder that contains .txt files with your data.
11D.1	Popup with Error: The iteration range parameter is NaN.	The input for the iteration range parameter is not a number (NaN).	Provide a number as input for iteration range parameter.
11D.2	Popup with Error: The time resolution parameter is NaN.	The input for the time resolution parameter is not a number (NaN).	Provide a number as input for the time resolution parameter.
11D.3	Popup with Error: The acceptance threshold is NaN.	The input for the acceptance threshold is not a number (NaN).	Provide a number as input for the acceptance threshold parameter.
11.G4	Popup with Error: The input for manual mode is NaN.	The input for the manual mode parameter is not a number (NaN).	Provide a number as input for the manual mode parameter.
	Popup with Error: The input for manual mode is smaller than 1.	The input for manual mode parameter is smaller than 1.	Provide a parameter value for manual mode that is larger than 1.
11.G5	Popup with Error: The mean baseline parameter is NaN.	The input for the mean baseline parameter is not a number (NaN).	Provide a number as input for the baseline parameter.
11.G6	Popup with Error: The time range for noise estimation is NaN.	The input for the time range for noise estimation is not a number (NaN).	Provide a number as input for the time range parameter.
	Pop-up with Error: 'FileName' contains more than two columns.	The input data is containing more than 2 columns.	Provide a .txt file with a single or double column. Multi-column files can be split

			into single columns using DataDuster. see step 3 to 9.
14	Pop-up with Error: 'FileName' contains NaN values.	Data contains values that are not a number (NaN).	Remove or replace NaN data point(s) by using DataDuster see step 3 to 9.
	Pop-up with Error: 'FileName' contains infinite values.	Data contains values that are infinite (Inf).	Remove or replace Inf data point(s) by using the DataDuster see step 3 to 9.
	Pop-up with Error: 'FileName' contains is not formatted properly.	The data may be the wrong filetype.	Check the file extension of the input data, which should be .txt.
		The data may contain characters	Alternatively, check if the input data contains characters.
	Pop-up: No significant steps detected.	Detected S^{max} of round 1 is below the acceptance threshold.	Decrease the acceptance threshold parameter in advanced settings (Step 11.D3).
15		Data does not contain significant steps.	An inherent feature of the input data, e.g. linear data, that cannot be solved.
	AutoStepfinder does not detect a significant portion of my events.	Base line type events: The baseline between events is too long.	Decrease the baseline between events. A typical range for effective step detection are baselines that have an equal or smaller dwell time than the events.
		Data exhibits big features (large-step sizes or long plateaus) that are not of interest.	Remove or decrease size of these features from dataset.
		Events are too short or too small to fulfill the S-criteria.	Inspect S-curve and if necessary tune fitting with sensitivity or manual mode.
	AutoStepfinder splits big steps into smaller unwanted steps.	Steps in the data are not instantaneous.	An inherent feature of the input data, no obvious solution.
		Data was low pass filtered or smoothened.	*AutoStepfinder* works best on instant steps, remove smoothing of data. Alternatively, consider step preserving filtering, such as median or Chung-Kennedy filtering.
	AutoStepfinder fits high frequency features that are not of interest.	Data contains high frequency features.	Consider step preserving filtering, such as median or Chung-Kennedy filtering. Alternatively, consider removing the features corresponding to the high frequency features from the fit. For example, by using the StepMerger auxiliary tool.
