# Supplementary material for: AutoStepfinder: A fast and automated step detection method for single-molecule analysis
Source: Patterns (N Y). 2021 Apr 30;2(5):100256. doi: 10.1016/j.patter.2021.100256 (PMC8134948; doi:10.1016/j.patter.2021.100256)
Supplement: Document S2. Article plus supplemental information [file mmc2.pdf]

Patterns

Descriptor

AutoStepfinder: A fast and automated step detection method for single-molecule analysis

Graphical abstract

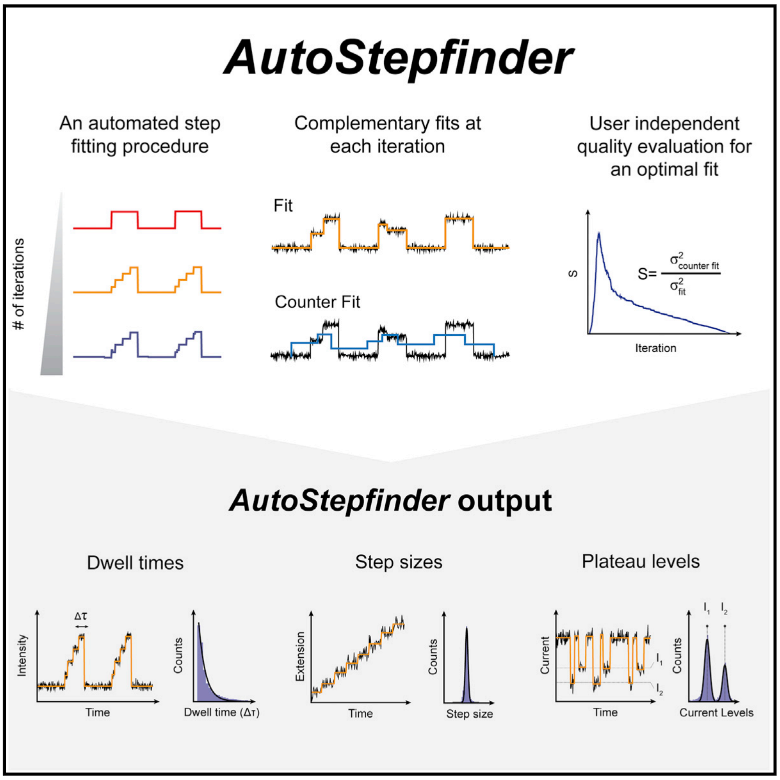

Authors

Luuk Loeff, Jacob W.J. Kerssemakers, Chirlmin Joo, Cees Dekker

Correspondence

c.joo@tudelft.nl (C.J.),
c.dekker@tudelft.nl (C.D.)

In brief

Loeff et al. report on an automated step detection method called *AutoStepfinder*. The algorithm allows researchers to determine the kinetic states within single-molecule time trajectories without any prior knowledge on the underlying noise contributions and step locations. A dual-pass strategy determines the optimal fit and allows *AutoStepfinder* to detect steps of a wide variety of sizes. The user-friendly interface and the automated detection of *AutoStepfinder* provides a robust analysis procedure that enables anyone without programming knowledge to generate step fits.

Highlights

- Fast, automated, and bias-free detection of steps within single-molecule trajectories
- Robust step detection without any prior knowledge on the data
- A dual-pass strategy for the detection of steps over a wide variety of scales
- A user-friendly interface for a simplified step fitting procedure

Loeff et al., 2021, Patterns 2, 100256
May 14, 2021 © 2021 The Authors.
https://doi.org/10.1016/j.patter.2021.100256

CellPress

Patterns

CellPress
OPEN ACCESS

Descriptor

AutoStepfinder: A fast and automated step detection method for single-molecule analysis

Luuk Loeff,[1,2,3] Jacob W.J. Kerssemakers,[1,3] Chirlmin Joo,[1,*] and Cees Dekker[1,4,*]
[1]Kavli Institute of Nanoscience and Department of Bionanoscience, Delft University of Technology, 2629 HZ Delft, The Netherlands
[2]Present address: Department of Biochemistry, University of Zurich, 8057 Zurich, Switzerland
[3]These authors contributed equally
[4]Lead contact
*Correspondence: c.joo@tudelft.nl (C.J.), c.dekker@tudelft.nl (C.D.)
https://doi.org/10.1016/j.patter.2021.100256

THE BIGGER PICTURE Single-molecule techniques have made it possible to track individual protein complexes in real time with a nanometer spatial resolution and a millisecond timescale. Accurate determination of the dynamic states within single-molecule time traces provides valuable kinetic information that underlie the function of biological macromolecules. Here, we present a new automated step detection method called *AutoStepfinder*, a versatile, robust, and easy-to-use algorithm that allows researchers to determine the kinetic states within single-molecule time trajectories without any bias.

1 2 **3** 4 5 **Development/Pre-production:** Data science output has been rolled out/validated across multiple domains/problems

SUMMARY

Single-molecule techniques allow the visualization of the molecular dynamics of nucleic acids and proteins with high spatiotemporal resolution. Valuable kinetic information of biomolecules can be obtained when the discrete states within single-molecule time trajectories are determined. Here, we present a fast, automated, and bias-free step detection method, *AutoStepfinder*, that determines steps in large datasets without requiring prior knowledge on the noise contributions and location of steps. The analysis is based on a series of partition events that minimize the difference between the data and the fit. A dual-pass strategy determines the optimal fit and allows *AutoStepfinder* to detect steps of a wide variety of sizes. We demonstrate step detection for a broad variety of experimental traces. The user-friendly interface and the automated detection of *AutoStepfinder* provides a robust analysis procedure that enables anyone without programming knowledge to generate step fits and informative plots in less than an hour.

INTRODUCTION

Over the last 25 years, single-molecule techniques have greatly enhanced our understanding of complex biological processes.[1,2] These techniques have made it possible to track the molecular dynamics of individual proteins and protein complexes with a (sub)nanometer spatial resolution and a (sub)millisecond timescale.[3,4] For example, molecular motor protein complexes were observed to move in a step-by-step fashion along cytoskeleton filaments.[5–7] More generally, force spectroscopy (using, e.g., optical or magnetic tweezers) has been exploited as a versatile tool for probing the forces and motions that are associated with biological macromolecules.[8,9] Single-molecule fluorescence techniques have been used to determine the stoichiometry, binding kinetics, and conformational dynamics of nucleic acids and proteins.[10–13] Nanopores have provided a powerful tool for the label-free detection of nucleic acids and proteins.[14,15]

Accurate determination of different states within a single-molecule time trace provides valuable information about the kinetic properties that underlie the function of biological macromolecules. For trajectories that display complex behavior, manual analysis is commonly practiced, in which a person with a trained eye picks out each state, a routine which, however, is prone to induce user bias. A common challenge in single-molecule data analysis is to distinguish these states in a reliable, reproducible, and unbiased manner. To facilitate reliable single-molecule trajectories, several automated step detection

This is an open access article under the CC BY license (http://creativecommons.org/licenses/by/4.0/).

methods have been developed over the years. Initial step detection methods relied on thresholding[16] or pairwise distribution analysis.[5,17] While these methods are capable of detecting clear state-to-state transitions, they do not suffice when the state transitions are close to the noise level and when the data exhibit multiple steps of variable size. Alternatively, statistical modeling can be used to extract kinetic information from single-molecule trajectories (see reviews by Colomb and Sarkar[18] and Tavakoli et al.[19]).

Early model-based step detection methods relied on the use of a generalized likelihood-ratio test to detect steps assuming Gaussian[20] or Poissonian[21] noise, without the need to make any kinetic model assumptions. Other statistical model-based step detection methods rely on the use of an information criterion (IC).[22–24] The general concept of these algorithms is to generate a variety of candidate models with steps at different locations.[19,22] Each of these models is scored on the goodness of the fit to the data, the number of used parameters in the model, and a term that penalizes each extra step that is added to the fit to prevent overfitting of the data.[22] The optimal model for the data is subsequently selected by minimizing the IC score, resulting in a "hands-off" fitting procedure.[22,25–29] For IC-based approaches, selecting the correct mathematical modeling of the noise contributions in the signal is crucial to obtain reliable fitting results, thereby requiring a full description of the noise in the data. Given that the sources of noise can vary substantially per experimental setup, it is difficult to make an IC-based algorithm that is applicable to a wide variety of trajectories.

One of the most commonly used approaches is based on hidden Markov modeling (HMM), which involves estimating the transition probabilities of a number of postulated states that are visited during the time course of an experiment. Various improvements were made to HMM, for example, by making use of a local and global HMM that allows to overcome the need for a state to successively occur within the same trajectory.[30] While HMMs have proven to be a powerful algorithm for the analysis of single-molecule trajectories, they are limited when it comes to analyzing systems with unknown dynamics. HMMs are often used in a supervised manner where the user provides parameters, such as the number of visited states and the allowed transitions between each state.[31–37] However, generally these parameters are unknown *a priori*[33,38,39] and require the user to sample a parameter space to find a suitable model. In a more objective approach, HMM is combined with Bayesian nonparametrics,[40] allowing one to use HMM without any knowledge on the number of visited states *a priori*. However, depending on the parameter space that is covered, this can dramatically increase the required computational time. In summary, HMMs are a powerful statistical tool, but it remains challenging to apply HMM models to systems with unknown system dynamics or when states are not frequently visited (e.g., bleaching data).

More recent algorithms have focused on combining model-based approaches with machine learning to allow unsupervised classification and idealization of single-molecule trajectories in a high-throughput manner.[41,42] While machine learning algorithms provide a powerful tool for high-throughput and unsupervised processing of data across a wide range of single-molecule techniques, the underlying models need to be (re)trained to work reliably on complex single-molecule trajectories.[41,42] Therefore, there is a need for explorative approaches, hereafter called first-order approaches, that are not tailored to a specific noise model and do not require information on the underlying states *a priori*. Such first-order approaches provide flexibility in interpreting and analyzing features in single-molecule trajectories and can provide important input for machine learning and model-based algorithms.

Previously, Kerssemakers et al. reported on a first-order approach called *Stepfinder*.[20] Given its robustness, flexibility, and simplicity, *Stepfinder* received great interest in the field of biophysics and was applied to the experimental trajectories from a wide variety of biological systems.[43–50] Despite its popularity, the algorithm faced several caveats: (1) the algorithm was subject to user bias, requiring the user to determine the final number of steps, (2) it was computationally demanding when presented with large datasets, (3) it failed in step evaluation when presented with data that exhibited a broad spectrum of step sizes, which especially holds true for baseline-type trajectories, and (4) it lacked a user-friendly interface. To overcome these short-comings, we here present a significantly revised and superior algorithm (*AutoStepfinder*) that facilitates high-throughput and automated step detection.[51,52]

AutoStepfinder has been designed as a first-order analysis tool that requires minimal knowledge of the location of steps and the various signal contributions in the data. A central feature of both *Stepfinder* and *AutoStepfinder* is the application of two complementary fits with an equal number of steps. To do so, this algorithm iteratively fits steps at locations that yield the biggest reduction in the variance (σ^2). Subsequently, the σ^2 of the fit is compared with the σ^2 of a worst-case-fit with the same number of steps, called a counter fit.[43,51,52] To assess the quality of the fit, the algorithm generates a step spectrum or S-curve that displays a sharp peak when the signal harbors step-like features, whereas smooth changes (such as drift) result in a flat S-curve. In contrast to *Stepfinder*, *AutoStepfinder* assesses the quality of the fit over multiple rounds, allowing for step analysis at various scales. This multiscale step fit procedure allows the researcher to evaluate which steps are relevant for the analysis.

The *AutoStepfinder* algorithm is a first-order step detection approach that provides a survey of the step-landscape and is complementary to more refined statistical methods that require a full description of the experimental parameters. The user-friendly interface of *AutoStepfinder* simplifies step detection in single-molecule trajectories, making it accessible to a wide variety of users. We illustrate the effectiveness of *AutoStepfinder* with a variety of different experimental traces from diverse single-molecule techniques. Taken together, the *AutoStepfinder* algorithm can be regarded as a robust and versatile tool that can be used for the many experimental cases where a full description of the noise in the data is unavailable.

RESULTS

Overview of the procedure

The workflow of the *AutoStepfinder* algorithm is outlined in Figure 1. *AutoStepfinder* can analyze the trajectories of a wide variety of single-molecule techniques (Figure 1A). The step-finding algorithm functions in three main steps: input of data, automated

CellPress
OPEN ACCESS

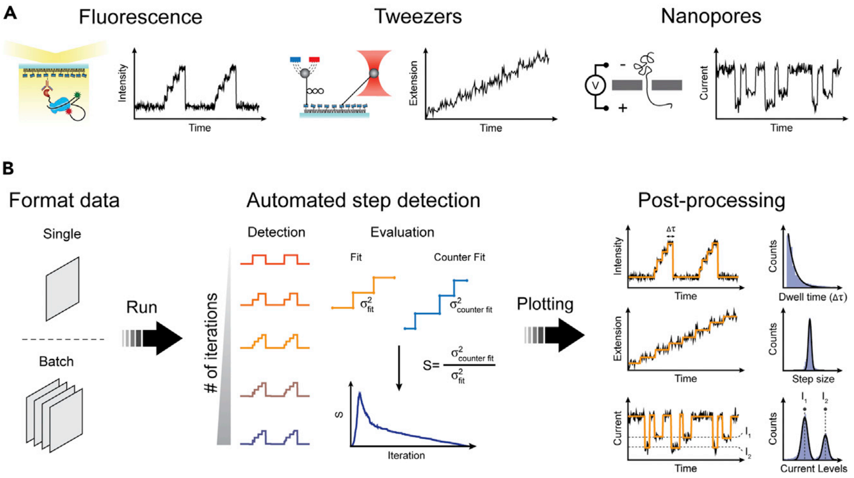

Figure 1. Workflow of *AutoStepfinder*

(A) *AutoStepfinder* can be applied on a wide variety single-molecule of trajectories, including single-molecule fluorescence, magnetic and optical tweezers, and nanopore data.

(B) The algorithm requires input in the form of one or multiple.txt files with one or two columns (signal value or time and signal value). After pressing run, the algorithm iteratively adds single steps to the data that minimizes the σ^2 value. For each iteration, the quality is assessed by means of a secondary counter fit. Finally, the best fit is selected and the algorithm outputs the corresponding fit, dwell times, step sizes, and levels. Fitting large datasets (>10^6 data points) can be done in less than 1 min with a desktop computer.

Also see Figure S1.

step detection, and output of the result (Figures 1B and S1). The *AutoStepfinder* algorithm can run on a single data file or on multiple files using a batch mode. Once input is retrieved, *AutoStepfinder* runs a first round of step fitting, in which the algorithm minimizes the variance (σ^2) between the data and the fit. During this procedure the data are iteratively split into multiple plateaus (Figure 1B). At each iteration, a new plateau is fitted at a location that yields the biggest reduction in σ^2. After each partition event, *AutoStepfinder* determines the quality of the fit by performing an additional fit (called a counter fit)[43,51,52] (Figures 1B and S1).

Once the most prominent steps are fitted during the first round of fitting and counter fitting, the *AutoStepfinder* algorithm subtracts the optimal fit from the data and executes the second round of iterative fitting and counter fitting on the residual data (Figure S1). This dual-pass strategy allows *AutoStepfinder* to facilitate step fitting of data with steps that vary widely in size. Once the optimal fit for the second round is determined, the algorithm generates a final fit by combining the step indices of the two rounds of fitting, and it outputs several files that allow post-processing of the results (Figures 1B and S1). This dual-pass step-fitting method provides a robust approach for automated step detection. The user-friendly interface and the automated detection of *AutoStepfinder* provides a hands-off fitting procedure that can be executed by anyone without programming knowledge.

Principles of step detection

The *AutoStepfinder* algorithm fits data through a series of partition events that minimize σ^2 (Figure 1). To fit data, the algorithm makes the sole assumption that the data contain instantaneous steps of interest with variable size (Δ_i) and plateau length (N_i). These plateaus are exposed to "noise," which can be defined as the residual variance (σ_R^2) from random signals that arise from the experimental setup (true noise) or features at a different scale that are not of interest (Figure 2A). The algorithm initiates the fitting procedure by fitting one step to all data points at a location that gives the lowest value of σ^2. This initial partition event generates a fit with two plateaus at a position that represents the average of the data points within the plateau (Figure 2A).[43] After the first fit, the plateau that exhibits a step yielding the largest reduction σ^2 is selected for the next partition event, resulting in a fit with three plateaus (Figure 2A, dashed red line). The algorithm continues this process of adding a single step to one of the plateaus for each iteration (Figure 2B, cyan arrow heads), until *AutoStepfinder* has performed the user-defined maximum iteration number (Figure S1).

AutoStepfinder successively selects one of previously fitted plateaus for the next partition event based on the biggest reduction in σ^2 (Figure 2A). This makes *AutoStepfinder* a so-called greedy algorithm that makes a locally optimal choice without considering its effect on the next step fits (see comparison of *AutoStepfinder* with other methods). Because *AutoStepfinder* iteratively prioritizes the next fit that gives the biggest reduction in σ^2, the most prominent features of the data are fitted first, followed by fits for the more refined features. The iteration continues until the user-defined number of steps is reached. Typically, this number is large enough so that the step fits is likely to go beyond any "optimal fit" (Figure 2B, middle). This results in "overfitting," where new steps are fitted to the noise of the data (Figure 2B, bottom).

Probing a step fit spectrum

Next, to determine the optimal fit for a given dataset, it is important to evaluate the quality of the fit for every step that is added to the analysis (Figure 1). The quality of the existing fit is evaluated by performing an additional fit for each iteration, hereafter called a counter fit.[43] *AutoStepfinder* generates such a counter fit by means of three steps: (1) *AutoStepfinder* determines the next partition location (i_{next}) within each plateau (Figure 3A); (2) the algorithm ignores the existing step locations; and (3) *AutoStepfinder* builds a new fit based on the i_{next} locations, generating new plateaus with a position that represents the average of the data points within each plateau (Figure 3A). These three steps result in a counter fit with steps that are all located in between the existing best-fit locations (Figure 3A). If the analyzed data do not display step-like behavior, both the existing fit and counter fit will have similar values of σ^2 (Figure S2).[43,51,52] However, when the data do display step-like behavior, counter fitting results in a fit that is much worse than the existing fit (Figure 3A) and thereby yields a larger value of σ.[2,43,51,52] To evaluate the

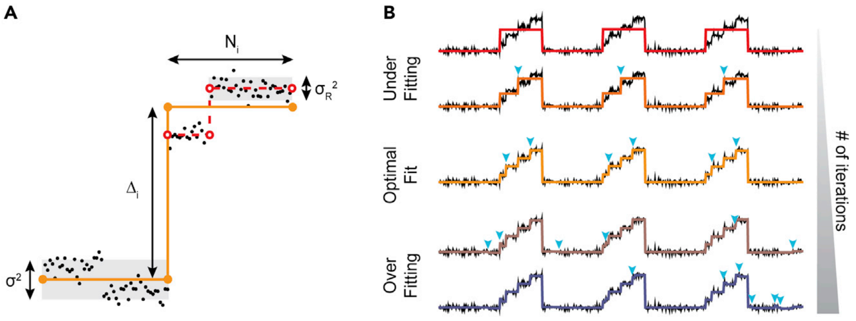

Figure 2. Global arrangement of the *AutoStepfinder* algorithm

(A) An example of an iterative step fit (orange line) on a single-molecule trajectory (black dots). Single-molecule trajectories are fitted by the *AutoStepfinder* algorithm by iteratively minimizing the σ^2 value. To perform a step fit the program assumes that the data contain steps (Δ_i), bounded by a plateau (N_i) that is subject to residual noise ($\sigma_R{}^2$, gray box). After the first step fit, *AutoStepfinder* selects the plateau with the largest value of σ^2, for the next partition event (red dashed lines). This process continues until the maximum number of iterations is reached.

(B) An example of the iterative process of step fitting by the *AutoStepfinder* algorithm. The algorithm successively adds a single step to the data (cyan triangles) and thereby minimizes the σ^2 value. Step fitting below the optimal number of steps is considered underfitting, whereas step fitting beyond the optimal number of steps is considered overfitting.
Also see Figure S1.

quality of a fit, the *AutoStepfinder* algorithm takes advantage of the sharply changing σ^2 landscape upon counter fitting.

The quality of a fit (S-score), can be quantified by taking the ratio of the σ^2 value from the existing fit and the counter fit, which is defined as:

$$S = \frac{\sigma^2_{counter\ fit}}{\sigma^2_{existing\ fit}}.$$

If the existing fit is at the optimal number of iterations, the variance of the existing fit approximates the residual variance in the data (σ^2). In contrast, the counter fit misses all real steps and places step location fits at random plateau positions. Thus, the fit values differ on average ½Δ from the data plateaus, yielding a σ^2 of the counter fit that reaches its maximum value ($\sim\Delta^2/4\sigma_R{}^2$).[43] Thereby, the maximum S-value (S^{max}) can be described by $S^{max} = 1 + P$, where P equals the maximum value of the counter fit ($\Delta^2/4\sigma_R{}^2$).[43] The strong difference of σ^2 between the fit and the counter fit when an optimal number of iterations is reached results in an S-value that is much larger than 1 (Figure 3). In contrast, when the data are under-fitted, the σ^2 value of the counter fit and existing fit approximate each other, resulting in an S-value that is close to 1 (Figure 3B). Similarly, overfitting a dataset, in which steps follow the noise, only results in a marginal change in the σ^2 value of the counter fit and thus the S-value also becomes close to 1 (Figures 2B, 3B, and 3C). Therefore, the S-curve is an effective indicator for stepped behavior in the analysis (Figure 3B). In effect, the S-curve provides an assessment of the step fit spectrum, displaying the scales at which steps occur in the signal as prominent peaks.

The use of two orthogonal fits allows *AutoStepfinder* to highlight features that evoke a strong discrepancy in the fit and counter fit S-values, which correspond to the most step-like features in single-molecule trajectories. The discrepancy between the two fits allows *AutoStepfinder* to use σ^2 minimization for very different kinds of noise types without a significant decay of its performance. To display this behavior, we generated an additional version of *AutoStepfinder* that uses a different error signal: the sum of absolute differences (SAD), and compared its behavior with *AutoStepfinder*, which uses minimization of the variance (σ^2). To compare the two algorithms, we generated trajectories that mimic stepped behavior of a kinesin-like motor protein with n_s steps of 8 nm that are bounded by plateaus with N_w points (Figure 3B). Comparison of the two versions of *AutoStepfinder* yielded S-curves with a sharp peak that was located at an identical global maximum (Figures 3B–3D).

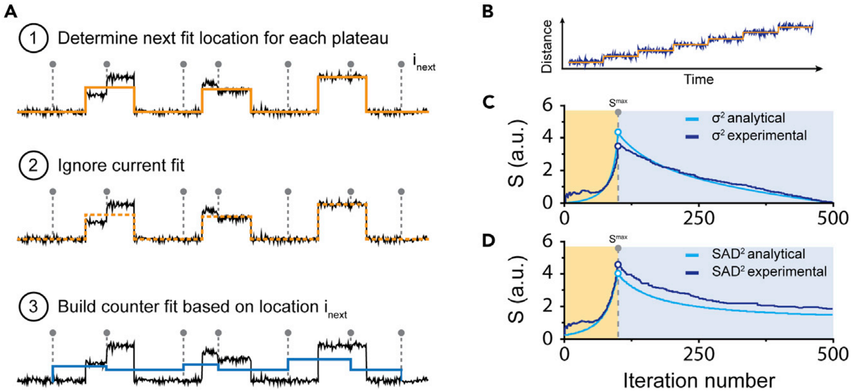

Figure 3. Determining the quality of a step fit

(A) For every step fit the algorithm performs, the quality of the fit (orange line) is evaluated by means of an additional fit (blue line, called a counter fit). The counter fit is built by determining the next partition point (i_{next}), after which the current is rejected. Subsequently, the algorithm places the counter fit (blue) plateaus at locations within the existing fit (orange).

(B) Simulated trajectory representing a motor stepping behavior.

(C) Representative example of experimental and analytical S-curves obtained by fitting the trajectory in (B) through minimization of σ^2. Shaded areas indicate the underfitting (yellow) and overfitting (light blue) regime.

(D) Representative example of experimental and analytical S-curves obtained by fitting the trajectory in (B) through minimization of the sum of absolute differences (SAD^2). Shaded areas indicate the underfitting (yellow) and overfitting (light blue) regime.
Also see Figure S2.

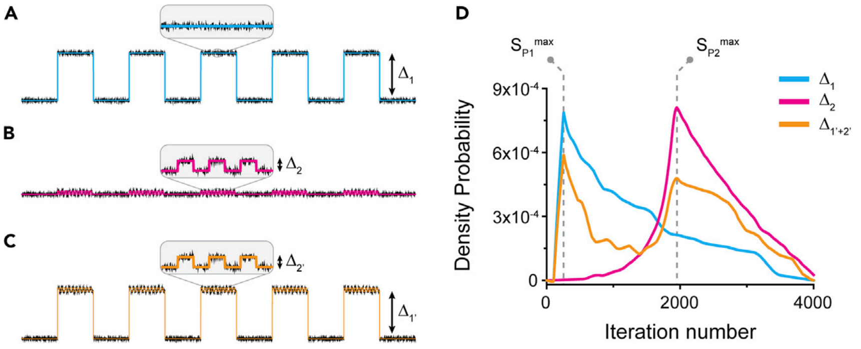

Figure 4. Dual-pass step detection to detect a wide range of step sizes

(A) A simulated single-molecule trajectory displaying uniform steps with a size of Δ1.

(B) An example trace displaying uniform steps with a size of Δ_2.

(C) A simulated single-molecule trajectory displaying non-uniform steps with a size of $\Delta_{1'}$ and $\Delta_{2'}$.

(D) S-curves for the three example traces displayed in (A–C). The global maximums of peak 1 (S_{P1}^{max}) and peak 2 (S_{P2}^{max}) are indicated with dashed gray lines. The S-curve for the dataset with both large (Δ_1) and small (Δ_2) steps exhibits two peaks.

Moreover, the simple nature of this signal allowed us to provide analytical approximations of the S-curves. The analytical solution of the S-curve using the variance for over and under fitting can be approximated by:

$$S_{f<1}(f) = \frac{P\left[\frac{2+f}{3}\right]+1}{2P(1-f)+1} \quad ; \quad S_{f>1}(f) = 1+\frac{P}{f}+\frac{(1-1/f)}{4N_f},$$

where N_f is the average location of the plateau, $P = \Delta^2/4\sigma_R^2$, and the relative fit fraction (f) can be described by $f = n_i/n_s$, where n_i is the actual number of fitted steps. In this equation the correct number of steps corresponds to f = 1. The analytical solution of the S-curve using the SAD can be approximated by:

$$S_{f<1}(f) = \frac{P_\omega\left(\frac{2+f}{3}\right)}{2P_\omega(1-f)+(2f-1)}; \quad S_{f>1}(f) = 1+\frac{P_\omega}{f}+\frac{1-1/f}{2N_f}-\frac{1}{f},$$

where $P_\omega = \Delta/2\omega$. These analytical solutions result in S-curves that reflect the observed trends for the two experimental S-curves (Figures 3C and 3D). Taken together, this comparison suggests that the exact nature of the residual noise does not need to be known for effective step fitting with *AutoStepfinder*. Thereby, *AutoStepfinder* constitutes an inherently robust first-order step analysis tool.

Step detection over a wide spectrum of sizes by a dual-pass strategy

Both *Stepfinder* and *AutoStepfinder* use the S-curve as a robust measure to determine the quality of a fit, showing a distinct peak when the optimal number of iterations is reached. When the steps in the data are within the same order of magnitude (e.g., Δ_1 or Δ_2 in Figures 4A and 4B), the *Stepfinder* algorithm would plot the S-curve and require the user to select the optimal fit by providing the number of iterations that corresponds to the global maximum of the S-curve (S^{max}) (Figure 4D). However, the optimal number of iterations cannot be determined through S^{max} when the data exhibit steps that vary widely in size. Especially when large and small steps are combined in a single-trajectory (e.g., $\Delta_{1'}$ and $\Delta_{2'}$, Figure 4C), the S-curve may exhibit multiple peaks or shoulders (S_{P2}) that have a lower S_{P2}^{max} than the first peak (S_{P1}^{max}) (Figure 4D). Notably, the position of these peaks is identical to the peaks observed for a dataset with either Δ_1 or Δ_2 (Figure 4D). In this case, the previous version of the algorithm (*Stepfinder*) is thereby not capable of suggesting an optimal fit for the data.

To facilitate step detection across a wide variety of scales, we developed a dual-pass strategy that determines the optimal fit for the data over two rounds (Figure S1). The *AutoStepfinder* algorithm first performs a step fit based on the global maximum of the S-curve (S_{P1}^{max}) that corresponds to the most prominent features in the data. This step fit is then subtracted from the data and a secondary step fit is performed on the "residual data." Only if the global maximum of the secondary step fit is above the user-defined threshold, coined "acceptance threshold," will the fit be accepted (Figure S1). The dual-pass approach combined with the acceptance threshold on the second round of fitting provides a robust method for automated step detection. Note that one might ponder yet deeper levels of refinement, with a third round of step fitting or beyond. We have explored this, but so far never encountered a case where more than two rounds were required. Note that under these circumstances, the existence of multiple peaks in the S-curve displays multiple scales of step sizes. Thereby, the S-curve functions as a step fit spectrum with peaks indicating different step size scales or marked step behavior. Depending on the experimental context, some of these scales may be of more or less of interest to the user.

Computationally efficient step detection with *AutoStepfinder*

AutoStepfinder is a so-called greedy algorithm[53] that iteratively selects an existing plateau (N_w) and splits it into a left (N_L) and a right (N_R) plateau (Figure S3A) at a location that results in the biggest reduction in σ^2. As a result, *AutoStepfinder* makes a locally optimal choice without considering its effect on the next step fits, significantly reducing the amount of computing power that is required to determine the fit. The position of these newly acquired plateaus is strongly dependent on the location of the partition point within N_w (Figure S3A). Therefore, *AutoStepfinder* calculates the average position (A) of a plateau (e.g., N_L) for any given location (i), which can be described by:

$$A_L = \frac{1}{N_L}\sum_{i=1}^{N_L} x(i).$$

These positions can be used to generate a σ^2 landscape that shows cusps at the optimal fitting positions (Figure S3B). While

misplacement of a step fit affects the slope of the remaining cusps, the minima positions remain identical, implying that the location of a step fit is not affected by prior and subsequent step fitting. The robustness of the cusp location justifies the use of a greedy procedure for step fitting by *AutoStepfinder*.

Despite the greedy nature of the algorithm, the iterative process of determining the partition point requires a substantial amount of computing power and becomes problematic when analyzing large datasets (e.g., >1 × 10^6 data points) (Figure S3C). Previously, *Stepfinder* determined the next partition point of N_w by calculating the σ^2 value for all possible locations (i), selecting the step fit that would yield the largest reduction in the σ^2 value. However, this meant that for a dataset with N_0 data points, the algorithm performed N_0^2 single x(i) operations to determine a single partition point. Next, the algorithm would repeat the same cycle to generate the next left (N_L) and right (N_R) plateau. With this scheme, this required ½N_0^2 single x(i) operations to locate the next two partition points. This cycle of partitioning continued to deduce plateaus until the algorithm reached the maximum number of iterations. In total, this yields a factor of (1+½+¼+ …) $\cdot N_0^2$ or roughly $2 \cdot N_0^2$ operations per dataset. Thereby, the required computing time increased significantly with an increase in the number of data points in a dataset (Figure S3C).

To reduce the operations that are required to fit a dataset, we comprehensively re-organized the code and streamlined the iteration process. A strong reduction in the number of required operations (i) can be made by re-using the information that is obtained during the localization of the first partition point. After the algorithm has determined the average (A_w) value of a plateau (N_w), *AutoStepfinder* determines the location of both N_L and N_R for x(i), using a single operation. The procedure starts with x(1) that is located at the left side of N_w (Figure S3A). The location (A_L) of N_L can be deduced by A_L(i) = x(i), whereas the level of N_R is defined by:

$$A_r(\mathrm{i}) = \frac{(N_w \bullet A_w - x(i))}{(N_w - 1)} .$$

This procedure is repeated for the next location (i + 1) until each location of N_w is calculated, requiring only N_0 operations per plateau. For a whole dataset, this scales with $2 \cdot N_0$, which is a gain of a factor of N_0 compared with the previous algorithm. Depending on the size of the analyzed dataset, this improvement yields a significant speed gain of several orders of magnitude (Figure S3B).

Quantifying the detection limits of *AutoStepfinder*

One of the major limiting factors in the detection of step-like behavior in single-molecule trajectories is noise, which can have various origins, such as thermal fluctuations of the biological system and the electronics of the measurement system (shot noise, thermal noise, 1/f noise).[54–56] Therefore, both the nature and the amount of noise in the single-molecule trajectories is highly dependent on the technique used to acquire the data. In its simplest form, the noise in single-molecule trajectories can be approximated as random white Gaussian noise, which can be characterized by the standard deviation of the noise.[26] As the noise intensity (SD) (Figure 2A) increases relative to the step size, step detection becomes significantly more challenging (Figure S4). Notably, to estimate the performance of *AutoStepfinder*, we compare the signal to noise ratio (SNR), which can be defined as: SNR = ½Δ/SD, where Δ represents the step size.

To probe the detection limitations of *AutoStepfinder*, we simulated data that was composed of a signal that featured a systematic decrease in step size. The data start with a step of 10 arbitrary units (a.u.), the subsequent steps decrease by 1 a.u. until the smallest step size of 1 a.u. is reached (Figure 5A). This idealized trajectory was repeated for 100 times, resulting in a dataset in which each step size occurred 100 times. Next, this dataset was exposed to various levels white of Gaussian noise (SD) and fitted with the *AutoStepfinder* algorithm (Figure 5A). When *AutoStepfinder* detects all states within the idealized trajectory, a histogram of the distribution of step sizes should be equally populated in each bin (Figure 5B, red dashed line).

For the idealized trajectories that were subject to noise with an SD of 0.1 and 0.25, *AutoStepfinder* correctly identified >98% all the steps in the trajectory (Figure 5B). However, when the SD of the noise was equal to the smallest step size in the trajectory (SD = 1.0, SNR = 0.5), *AutoStepfinder* detected only 2% of the smallest step size of 1 a.u. and ~50% of the steps that were 2 a.u. in size (SNR = 1.0). As a consequence of the missed steps in this regime, *AutoStepfinder* overestimated (~150%) steps that were 3 a.u. in size. This trend continued when the SD of the noise was twice the size of the smallest step in the trajectory (SD = 2.0, SNR = 0.25) (Figure 5B).

To further quantify the response of *AutoStepfinder* to noise, we generated several benchmark traces (Figure 5C, bottom) at different noise levels by injecting steps with various sizes (Δ_{inject}, Figure 5C, middle) at known locations into an existing trajectory (Figure 5C, top). To generate statistically relevant data, this process was repeated 100 times for each noise level, randomizing Δ_{inject} between the values 0 and 1. We subsequently quantified the probability that *AutoStepfinder* would detect the injected steps (Figure 5D). *AutoStepfinder* shows a sharp cutoff in its detection probability (Figure 5D), which shifts toward larger step sizes when noise is increased and smaller steps are drowned in the noise. We note that our conservative choice of short-lived plateaus (<50 data points) increases the associated error within the steps. Under these circumstances, *AutoStepfinder* is effective (i.e., deduces steps with a >50% detection probability) in detecting steps that are twice the size of the SD of the noise ($\Delta_{detected}$ = ½SD, SNR = 1). We note that, to estimate the reliability of the obtained results, the step injection test can also be applied to experimental data, as we previously reported in a case study (see Eeftens et al.[51]).

To determine the uncertainty in the placed steps, we implemented a bootstrap analysis function[57] in *AutoStepfinder* that closely follows the error estimation by Li et al.[58] In brief, once *AutoStepfinder* has determined the fit, each plateau is resampled (typically 1,000 times) by bootstrap analysis and the relative positions between neighboring plateaus is re-assessed, allowing the algorithm to determine the 95% confidence interval of the step sizes. In addition, this procedure provides a confidence interval of the variance, which allows for a direct estimation of the 95% confidence interval of the step time (Figure S3B, shaded areas).[58] As a result, prominent steps that are associated with a sharp cusp in the variance have smaller 95% confidence

CellPress
OPEN ACCESS

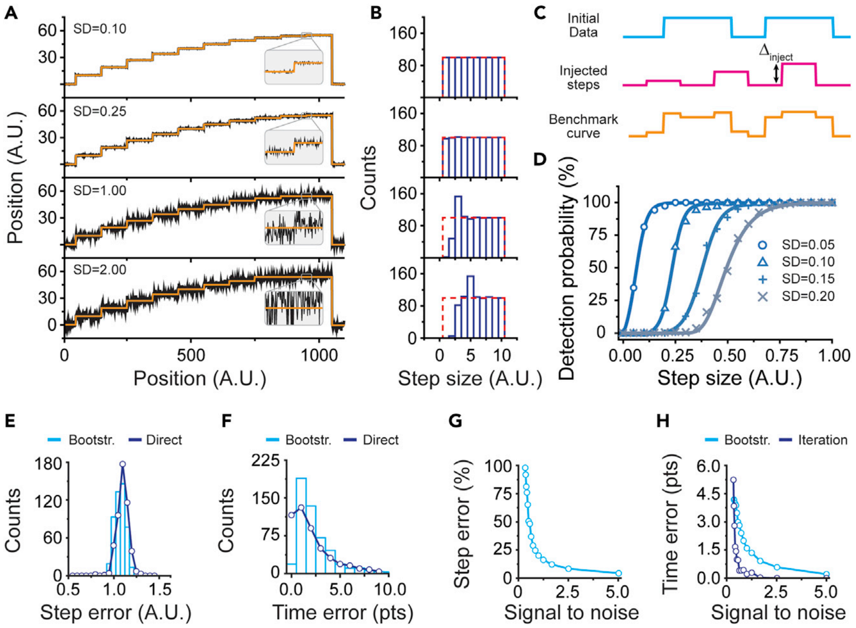

Figure 5. Testing the detection limits of *AutoStepfinder*

(A) Simulated time trajectories that were exposed to various noise levels to benchmark the *AutoStepfinder* algorithm. The data start with a step of 10 arbitrary units (a.u.), the subsequent steps decrease by 1 a.u. until the smallest step size of 1 a.u. is reached. This idealized trajectory was repeated 100 times, resulting in a dataset in which each step size occurred 100 times.

(B) Distribution of step sizes of the simulated trajectories, obtained through the *AutoStepfinder* algorithm. The red dashed lines indicate the position of each bin when 100% of the steps are correctly identified.

C) Schematic of the step injection test. To quantify the probability that *AutoStepfinder* would detect steps with a certain size (Δ_{inject}), steps were injected (pink curve, middle) into an existing trajectory (blue curve, top) to generate a benchmark curve (orange curve, bottom).

(D) Histogram of the detection probability of step sizes at various noise levels (SD). Solid lines represent sigmoidal fits to the data.

(E) Histogram showing the distribution of the 95% confidence intervals of the step sizes (cyan bars) obtained by bootstrap analysis. The line (purple) indicates the deviation of the fit from the ground truth.

(F) Histogram showing the distribution of the 95% confidence intervals of the plateaus lengths (cyan bars) obtained by bootstrap analysis. The line (purple) indicates the deviation of the fit from the ground truth.

(G) Relation between the SNR and the error in the determined steps.

(H) Relation between the SNR and the error in the determined plateaus (cyan line). The purple line indicates the deviation between the final fit and a local refit at various noise levels (iteration error).

Also see Figure S4.

intervals, whereas steps with a broad minimum are associated with larger errors. To validate this approach, we simulated trajectories that mimic the stepping behavior of a motor protein (Figure 3B) at an SNR of 1 and compared the bootstrapped confidence intervals with the deviation of the *AutoStepfinder* output to the absolute solution (hereafter called direct error). This validation shows that the bootstrapped confidence intervals provide an accurate estimation of the errors associated with the step sizes (Figure 5E) and step times (Figure 5F).

We further benchmarked the *AutoStepfinder* algorithm and probed how the error landscape develops when the amount of noise increases. As expected, the 95% confidence intervals associated with the step size (Figure 5G) and step time (Figure 5H) increase when the SNR becomes smaller. To assess if the greedy nature of *AutoStepfinder* causes deviation from the true location of the fitted steps, we redetermined the optimal fit locally and compared its location with the locations in the final fit by *AutoStepfinder* (hereafter called iteration error). These results show that the greedy step search provides an accurate description of the stepped behavior in the trajectories. Based on the results of these benchmarks, we conclude that *AutoStepfinder* provides an accurate fit down to an SNR of 0.75.

While noise is the major determinant for step detection, the frequency of occurrence of steps may also influence capability of *AutoStepfinder* to detect steps. As described in the section "Principles of step detection." *AutoStepfinder* selects the next step fit (R_{next}) based on a ranking system, choosing the step that yields the largest reduction in σ^2. This ranking system is based on the expected squared relative accuracy, which can be defined as:

$$R_{next} = \frac{\Delta^2}{{}^{1}/_{N_L} + {}^{1}/_{N_R}},$$

where Δ corresponds to the step size and N_L and N_R correspond to the number of data points in the left and right plateau, respectively. Consequently, fits with a large step size (Δ) or a large window size (N_i) are prioritized. This has important implications when fitting trajectories that have a long baseline. Since *AutoStepfinder* considers the baseline as a plateau, it prioritizes long baselines for step fitting when the data exhibit a sparse density of short events. Thereby, *AutoStepfinder* may not detect these sparse events. As a rule of thumb, it is advised to truncate the dwell time of the base line when it is >10 times longer than the dwell time of the events. For optimal fitting results, we advise to use a baseline with a duration that is in the same order as the dwell time of the events.

Comparison of *AutoStepfinder* with other methods

The *AutoStepfinder* algorithm was designed as a robust fitting tool that provides a first-order fitting approach of experimental trajectories where a full mathematical description of the noise in the data is unattainable. To benchmark *AutoStepfinder*

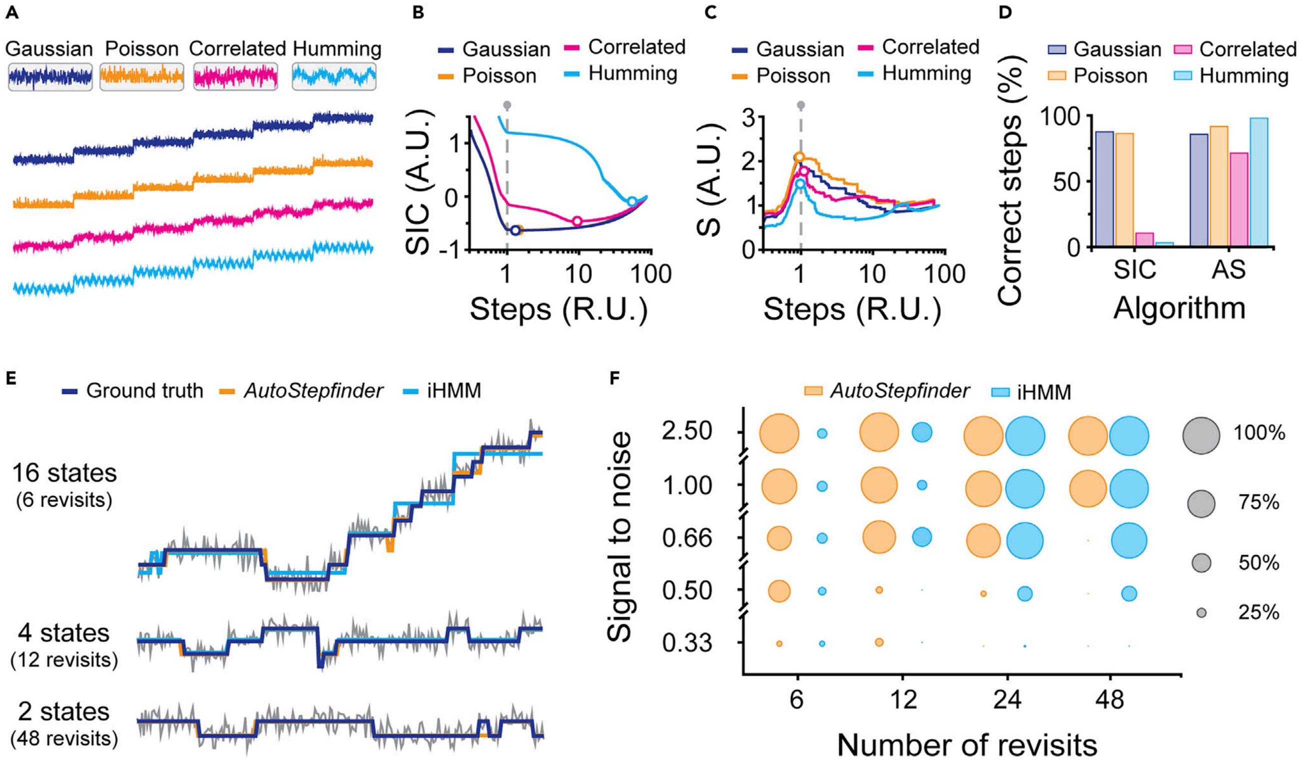

Figure 6. Comparison of *AutoStepfinder* with other methods

(A) Examples of simulated single-molecule trajectories that were exposed to distinct noise types, each with an SD of 2.0. The noise types are Gaussian noise (purple), Poissonian noise (orange), correlated noise (pink), and humming noise (light blue).

(B) Step detection by a Schwarz information criterion (SIC)-based algorithm.[22] For each step fit, the quality of the fit is evaluated by calculating an SIC score. The SIC curve displays a minimum when the optimal fit is reached (circle). The dashed gray line indicates the number of steps in the data. Notably, the SIC curve of Gaussian noise (purple) overlaps the SIC curve of the Poissonian noise (orange).

(C) Step detection by the *AutoStepfinder* algorithm. For each step fit, the quality of the fit is evaluated by performing an additional fit, called counter fit, and calculating an S-score. The S-curve displays a maximum when the optimal fit is reached (circle). The dashed gray line indicates the number of steps in the data.

(D) Performance of the *AutoStepfinder* algorithm and SIC-based algorithm on simulated single-molecule trajectories that were exposed to distinct noise types with SD = 2.0. A more extensive overview on the robustness of *AutoStepfinder*- and the SIC-based algorithms[22] is provided in Figure S4.

(E) Examples of simulated single-molecule trajectories each with a distinct number of states (gray). The purple, orange, and cyan lines indicate the ground truth, states found by *AutoStepfinder*, and the states found by iHMM,[33] respectively. The displayed trajectories were exposed to Gaussian noise with an SNR of 1.0.

(F) Comparison of *AutoStepfinder* (orange) and iHMM[33] (cyan). The size of the circles indicates the percentage states that were within a distance of 25% of a step size of the ground truth. The circles in gray indicate the percentage scale.

Also see Figure S5.

against a wide variety of noise types, we simulated data featuring a signal that systematically varied over time in a stepwise manner (Figure 6A). This signal was exposed to four different types of noise with the same SD: Gaussian noise, Poisson noise, and two other noise artifacts that are commonly found in single-molecule trajectories (Figure 6A): correlated noise that results in irregular correlated features, and humming noise, e.g., often associated with a line frequency (Figure 6A). Apart from *AutoStepfinder*, we used a Schwarz IC (SIC)-based algorithm[22] tailored to Gaussian noise in our benchmark.

For the simulated data that were exposed to Gaussian noise, both *AutoStepfinder* and the Gaussian-based SIC algorithm[22] fitted a similar number of steps across the range of noise (SD) tested (Figures 6 and S5A–S5C). Both algorithms correctly identified 98% of the steps at low noise levels (SD = 0.2), which decreased to approximately 50% at when the noise level was increased (SD = 2.0) (Figures 6D, S5D, and S5H). Thus, under ideal conditions, where a full mathematical description of the noise is available (i.e., for conditions optimal for SIC), *AutoStepfinder* performs equally well as compared with SIC-based algorithms. Next, the benchmark was repeated on trajectories with Poissonian, correlated, and humming noise. When subjected to these trajectories, *AutoStepfinder* was still capable of correctly identifying the states of interest in the data with any of these types of noise (Figures 6C, 6D, and S5). Notably, when we repeated the benchmark with the Gaussian-based SIC algorithm,[22] the analysis yielded strongly diverging results as expected (Figures S4 and S5). These results show that *AutoStepfinder* performs robustly over a broad spectrum of noise types.

Next, we compared the performance of *AutoStepfinder* against infinite HMM (iHMM),[33] a hands-off HMM-based algorithm that is developed to determine a limited number of states in a trajectory without making parameter assumptions *a priori*.[33]

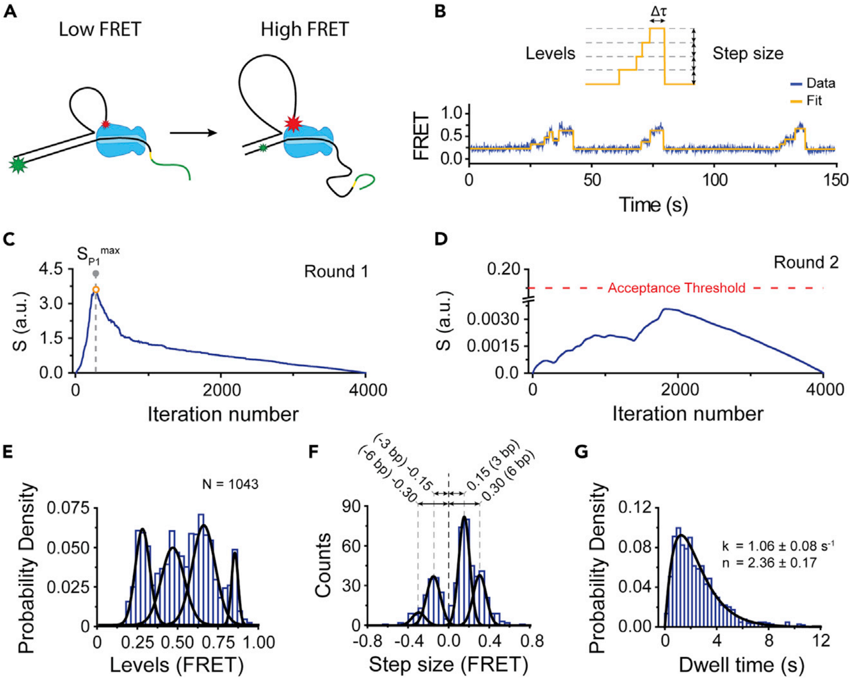

Figure 7. Application of *AutoStepfinder* on experimental FRET data

(A) Schematic of loop formation by the CRISPR-associated Cas3 helicase/nuclease protein (blue). The appearance of FRET during loop formation is indicated by the size of the star: low FRET, large green star, or high FRET, large red star.

(B) A representative FRET trace (dark blue) fitted with the *AutoStepfinder* algorithm (orange).

(C) S-curve for the first round of fitting by *AutoStepfinder*. The dashed gray line indicates the S_{P1}^{max} of the S-curve.

(D) S-curve for the second round of fitting by *AutoStepfinder*. The global maximum of the S-curve for the second round was below the set acceptance threshold and therefore the second round of fitting was not executed.

(E) Distribution of FRET levels obtained through the *AutoStepfinder* algorithm. Black lines represent a Gaussian fit.

(F) Distribution of step sizes obtained through the *AutoStepfinder* algorithm. Data were fitted with a gamma distribution (solid line) to obtain the number of hidden steps (n) and rate (k). Error represents the 95% confidence interval obtained through bootstrap analysis.

(G) Dwell time distribution obtained through the *AutoStepfinder* algorithm. Black lines represent a gamma distribution.

Also see Figure S6.

To benchmark both algorithms, we generated trajectories with a number of states (N) that have a mean step size of 10 a.u. between them. At each time point within the state there is a 4% chance of making a transition to a higher state and a 1% chance of a transition to a lower state. This model allows one to generate a diverse set of trajectories by only changing the number of states that are present within the data (Figure 6E). For example, a low number of states implies that the chance that a state is revisited is high, generating trajectories that resemble the output of single-molecule fluorescence measurements. In contrast, for a large number of states the chance of revisiting a state becomes low, resulting in trajectories that resemble the motor stepping behavior in magnetic and optical tweezer measurements.

To compare *AutoStepfinder* and iHMM over a wide variety of signals, we generated trajectories that were limited in time with various amounts of revisits per state (6, 12, 24, and 48 times). In addition, we subjected the trajectories to different amounts of Gaussian noise, ranging from an SNR of 0.25 up to 1.5. To obtain output from *AutoStepfinder* that mimics HMM, we performed k-means clustering on the output of *AutoStepfinder*, which clusters the fitted levels into the same number of initial states as were used for iHMM. Next, we compared the output of both algorithms on each trajectory against the ground truth. All states that deviated more than 25% of a step size of the ground truth were rejected, whereas the states within 25% of a step size of the ground truth were counted as detected.

This benchmark shows that *AutoStepfinder* performs robustly and independently from the number of revisits of each state, detecting <95% of the states in the low-noise regime. For trajectories that contained only a limited number of revisits per state (Figure 6F, 6 and 12 revisits) *AutoStepfinder* outperformed the iHMM algorithm. In contrast, the performance of the iHMM algorithm increased with an increased number of revisits per state. For trajectories where a state was frequently revisited (Figure 6F, 48 revisits), iHMM outperformed *AutoStepfinder* by correctly detecting more states at a higher noise level. In conclusion, we show that *AutoStepfinder* and iHMM are complementary algorithms, where *AutoStepfinder* is favored for trajectories with limited state-to-state transitions, and where HMM is favored for trajectories with many state-to-state transitions.

Step fitting of experimental data

Execution of the user manual (see supplemental information) described in this paper yields a robust step-detection analysis of single-molecule trajectories (Figure 7). To demonstrate the power of *AutoStepfinder* for one example in more detail, we applied the algorithm on single-molecule fluorescence resonance energy transfer (FRET) trajectories of the CRISPR-associated helicase Cas3. A detailed description on the experimental procedures is described in Loeff et al.[52] In brief, DNA-bound Cas3 molecules were presented with ATP to unwind DNA. The fluorophores on the DNA substrate reported on DNA unwinding through an increase in FRET (Figure 7A). Before ATP was added, the labeling positions on the DNA yielded a FRET value that was indistinguishable from the background signals. Upon addition of ATP, a stepwise increase in FRET was observed and was analyzed using *AutoStepfinder* (Figure 7B).

The first round of step fitting by *AutoStepfinder* yielded a sharp peak in the S-curve (Figure 7C). In contrast, the second round of step fitting yielded a global maximum below the acceptance threshold and was therefore not executed (Figure 7D). This indicates that *AutoStepfinder* detected steps which had a step size distribution within the same order of magnitude. Next, we used the step *Properties* file (Table S1) to generate histograms of

the FRET levels (Figure 7E), step size (Figure 7F), and dwell time (Figure 7G). These histograms show that the helicase moves along the DNA in well-defined steps, resulting in four equally spaced peaks in the FRET level histogram and a dominant peak at 0.15 in the step size histogram. Given that *AutoStepfinder* runs on any signal that exhibits step-like behavior, the algorithm is widely applicable on the trajectories of single-molecule techniques, including force spectroscopy[51] (Figure S6A–S6C) and nanopore data (Figure S6D–S6F).[59] Notably, in contrast to the single-molecule FRET data, both the examples of force spectroscopy and nanopore data required dual-pass data fitting (Figures S6B and S6E). Taken together, these analyses show that the *AutoStepfinder* algorithm can detect steps in a wide variety of single-molecule trajectories without any prior knowledge on their size and position.

DISCUSSION

AutoStepfinder is a robust and sensitive first-order step analysis tool that allows step fitting of single-molecule trajectories without any prior knowledge on the step size, step location, and noise contributions within the data. By probing the quality of the fit for every step that is added to the analysis, *AutoStepfinder* provides an assessment of the step fit spectrum (S-curve) within the data. This allows the user to perform a component analysis on the data, where steps at different scales within the data are separated from each other during the analysis. Our benchmark shows that the S-curve provides a robust quality assessment of the steps within the data, displaying a sharp peak when the data are fitted with correct number of steps at each scale.

While the S-curve provides a strong indication of the best solution, users may want to fine-tune the fit by focusing on steps of a particular scale in the data. For example, one may be interested in the large steps in the data, rather than the small steps within each plateau. The user-friendly interface of *AutoStepfinder* provides an environment that allows the user to make an educated decision on which features to fit based on the local maxima within the S-curve. Alternatively, based on the outcome of *AutoStepfinder*, one may design a model-based approach (AIS, SIC) to further fine-tune the fit or use the output of *AutoStepfinder* for machine-learning-based algorithms for high-throughput unsupervised classification and fitting of complex single-molecule trajectories.

Taken together, *AutoStepfinder* facilitates high-throughput step detection with minimal user input within a user-friendly environment that is both robust and sensitive, allowing users to fit experimental single-molecule trajectories without any prior knowledge on the noise and steps within the data. Moreover, given that *AutoStepfinder* is a versatile approach that can be applied on any signal with step-like behavior, we envision that the *AutoStepfinder* algorithm can be used beyond the field of single-molecule biophysics.

EXPERIMENTAL PROCEDURES

Resource availability

Lead contact

Further information and requests for resources should be directed to and will be fulfilled by the lead contact, Cees Dekker (c.dekker@tudelft.nl).

Materials availability

There are no physical materials associated with this study.

Data and code availability

The AutoStepfinder algorithm can be accessed at Zenodo: https://doi.org/10.5281/zenodo.4657659.

SUPPLEMENTAL INFORMATION

Supplemental information can be found online at https://doi.org/10.1016/j.patter.2021.100256.

ACKNOWLEDGMENTS

We would like to thank Jorine Eeftens for providing magnetic tweezer data, Laura Restrepo Perez for providing nanopore data, and Marileen Dogterom for contributions to the earlier version of *Stepfinder* and for critically reading this manuscript. C.D. was funded by the ERC Advanced Grant LoopingDNA (no. 883684) and The Netherlands Organization of Scientific Research (NWO/OCW) as part of the Frontiers of Nanoscience Program. C.J. was funded by Vidi (864.14.002) of the Netherlands Organisation for Scientific Research.

AUTHOR CONTRIBUTIONS

L.L., J.K., C.J., and C.D. conceived the study. L.L. and J.K. developed the code. L.L. and J.K. analyzed the data. L.L., J.K., C.J., and C.D. discussed the data and wrote the manuscript.

DECLARATION OF INTERESTS

The authors declare no competing financial interests.

Received: September 14, 2020
Revised: October 12, 2020
Accepted: April 8, 2021
Published: April 30, 2021

REFERENCES

1. Juette, M.F., Terry, D.S., Wasserman, M.R., Zhou, Z., Altman, R.B., Zheng, Q., and Blanchard, S.C. (2014). The bright future of single-molecule fluorescence imaging. Curr. Opin. Chem. Biol. *20*, 103–111.
2. Ha, T. (2014). Single-molecule methods leap ahead. Nat. Methods *11*, 1015–1018.
3. Forties, R.A., and Wang, M.D. (2014). Minireview discovering the power of single molecules. Cell *157*, 4–7.
4. Joo, C., Fareh, M., and Narry Kim, V. (2013). Bringing single-molecule spectroscopy to macromolecular protein complexes. Trends Biochem. Sci. *38*, 30–37.
5. Svoboda, K., Schmidt, C.F., Schnapp, B.J., and Block, S.M. (1993). Direct observation of kinesin stepping by optical trapping interferometry. Nature *365*, 721–727.
6. Yildiz, A., Tomishige, M., Vale, R.D., and Selvin, P.R. (2004). Kinesin walks hand-over-hand. Science *303*, 676–678.
7. Kaseda, K., Higuchi, H., and Hirose, K. (2003). Alternate fast and slow stepping of a heterodimeric kinesin molecule. Nat. Cell Biol. *5*, 1079–1082.
8. Sun, B., and Wang, M.D. (2015). Single-molecule perspectives on helicase mechanisms and functions. Crit. Rev. Biochem. Mol. Biol. *9238*, 1–11.
9. Ha, T., Kozlov, A.G., and Lohman, T.M. (2012). Single-molecule views of protein movement on single-stranded DNA. Annu. Rev. Biophys. *41*, 295–319.
10. Aggarwal, V., and Ha, T. (2016). Single-molecule fluorescence microscopy of native macromolecular complexes. Curr. Opin. Struct. Biol. *41*, 225–232.
11. Fareh, M., Loeff, L., Szczepaniak, M., Haagsma, A.C., Yeom, K.-H., and Joo, C. (2016). Single-molecule pull-down for investigating protein-nucleic acid interactions. Methods *105*, 99–108.

12. Blosser, T.R., Loeff, L., Westra, E.R., Vlot, M., Künne, T., Sobota, M., Dekker, C., Brouns, S.J.J., and Joo, C. (2015). Two distinct DNA binding modes guide dual roles of a CRISPR-Cas protein complex. Mol. Cell *58*, 60–70.
13. Gallardo, I.F., Pasupathy, P., Brown, M., Manhart, C.M., Neikirk, D.P., Alani, E., and Finkelstein, I.J. (2015). High-throughput universal DNA curtain arrays for single-molecule fluorescence imaging. Langmuir *31*, 10310–10317.
14. Restrepo-Pérez, L., Joo, C., and Dekker, C. (2018). Paving the way to single-molecule protein sequencing. Nat. Nanotechnol. https://doi.org/10.1038/s41565-018-0236-6.
15. Venkatesan, B.M., and Bashir, R. (2011). Nanopore sensors for nucleic acid analysis. Nat. Nanotechnol. https://doi.org/10.1038/nnano.2011.129.
16. Mckinney, S.A., Lilley, D.M.J., and Ha, T. (2003). Structural dynamics of individual Holliday junctions. Nat. Struct. Mol. Biol. *10*, 93–97.
17. Kuo, S.C., Gelles, J., Steuer, E., and Sheetz, M.P. (1991). A model for kinesin movement from nanometer-level movements of kinesin and cytoplasmic dynein and force measurements. J. Cell Sci. *14*, 135–138.
18. Colomb, W., and Sarkar, S.K. (2015). Extracting physics of life at the molecular level: a review of single-molecule data analyses. Phys. Life Rev. *13*, 107–137.
19. Tavakoli, M., Taylor, J.N., Li, C.B., Komatsuzaki, T., and Pressé, S. (2017). Single molecule data analysis: an introduction. Adv. Chem. Phys. *162*, 205–305.
20. Montiel, D., Cang, H., and Yang, H. (2006). Quantitative characterization of changes in dynamical behavior for single-particle tracking studies. J. Phys. Chem. B. https://doi.org/10.1021/jp062024j.
21. Watkins, L.P., and Yang, H. (2005). Detection of intensity change points in time-resolved single-molecule measurements. J. Phys. Chem. B. https://doi.org/10.1021/jp0467548.
22. Kalafut, B., and Visscher, K. (2008). An objective, model-independent method for detection of non-uniform steps in noisy signals. Comput. Phys. Commun. *179*, 716–723.
23. Schwarz, G. (1978). Estimating the dimension of a model. Ann. Stat. *6*, 461–464.
24. Akaike, H. (1973). Information theory and an extension of the maximum likelihood principle. Int. Symp. Inf. Theory. https://doi.org/10.1007/978-1-4612-1694-0.
25. Bronson, J.E., Fei, J., Hofman, J.M., Jr, R.L.G., and Wiggins, C.H. (2009). Learning rates and states from biophysical time series: a Bayesian approach to model selection and single-molecule FRET data. Biophys. J. *97*, 3196–3205.
26. Carter, B.C., Vershinin, M., and Gross, S.P. (2008). A comparison of step-detection methods: how well can you do? Biophys. J. *94*, 306–319.
27. LaMont, C.H., and Wiggins, P.A. (2015). Information-based inference for singular models and finite sample sizes. Arxiv, 1–12. https://arxiv.org/pdf/1506.05855.pdf.
28. Wiggins, P.A. (2015). An information-based approach to change-point analysis with applications to biophysics and cell biology. Biophys. J. *109*, 346–354.
29. Tsekouras, K., Custer, T.C., Jashnsaz, H., Walter, N.G., and Pressé, S. (2016). A novel method to accurately locate and count large numbers of steps by photobleaching. Mol. Biol. Cell *27*, 3601–3615.
30. Schmid, S., Götz, M., and Hugel, T. (2016). Single-molecule analysis beyond dwell times: demonstration and assessment in and out of equilibrium. Biophys. J. https://doi.org/10.1016/j.bpj.2016.08.023.
31. Mckinney, S.A., Joo, C., and Ha, T. (2006). Analysis of single-molecule FRET trajectories using hidden Markov modeling. Biophys. J. *91*, 1941–1951.
32. Greenfeld, M., Pavlichin, D.S., Mabuchi, H., and Herschlag, D. (2012). Single molecule analysis research tool (SMART): an integrated approach for analyzing single molecule data. PLoS One *7*.
33. Sgouralis, I., and Presse, S. (2017). Biophysical perspective an introduction to infinite HMMs for single-molecule data analysis. Biophys. J. 2021–2029. https://doi.org/10.1016/j.bpj.2017.04.027.
34. Blanco, M., and Walter, N.G.; Elsevier Inc. (2010). Analysis of Complex Single-Molecule FRET Time Trajectories. Single Molecule Tools: Fluorescence Based Approaches, *472* (Methods in Enzymology), pp. 153–178.
35. Hadzic, M.C.A.S., Börner, R., König, S.L.B., Kowerko, D., and Sigel, R.K.O. (2018). Reliable state identification and state transition detection in fluorescence intensity-based single-molecule Förster resonance energy-transfer data. J. Phys. Chem. B. https://doi.org/10.1021/acs.jpcb.7b12483.
36. Juette, M.F., Terry, D.S., Wasserman, M.R., Altman, R.B., Zhou, Z., Zhao, H., and Blanchard, S.C. (2016). Single-molecule imaging of non-equilibrium molecular ensembles on the millisecond timescale. Nat. Methods. https://doi.org/10.1038/nmeth.3769.
37. Van De Meent, J.W., Bronson, J.E., Wiggins, C.H., and Gonzalez, R.L. (2014). Empirical Bayes methods enable advanced population-level analyses of single-molecule FRET experiments. Biophys. J. https://doi.org/10.1016/j.bpj.2013.12.055.
38. Hines, K.E. (2015). A primer on Bayesian inference for biophysical systems. Biophys. J. *108*, 2103–2113.
39. Hines, K.E., Bankston, J.R., and Aldrich, R.W. (2015). Analyzing single-molecule time series via nonparametric Bayesian inference. Biophys. J. *108*, 540–556.
40. Ferguson, T.S. (1973). A Bayesian analysis of some nonparametric problems. Ann. Stat. *1*, 209–230.
41. White, D.S., Goldschen-Ohm, M.P., Goldsmith, R.H., and Chanda, B. (2020). Top-down machine learning approach for high-throughput single-molecule analysis. eLife. https://doi.org/10.7554/eLife.53357.
42. Thomsen, J., Sletfjerding M B, Jensen S B, Stella S, Paul B, Malle M G, Montoya G, Petersen T C, and Hatzakis N C. (2020). DeepFRET, a software for rapid and automated single-molecule FRET data classification using deep learning. eLife. https://doi.org/10.7554/eLife.60404.
43. Kerssemakers, J.W.J., Munteanu, E.L., Laan, L., Noetzel, T.L., Janson, M.E., and Dogterom, M. (2006). Assembly dynamics of microtubules at molecular resolution. Nature *442*, 709–712.
44. Myong, S., Bruno, M.M., Pyle, A.M., and Ha, T. (2007). Spring-loaded mechanism of DNA unwinding by hepatitis C virus NS3 helicase. Science, 513–517.
45. Dame, R.T., Noom, M.C., and Wuite, G.J.L. (2006). Bacterial chromatin organization by H-NS protein unravelled using dual DNA manipulation. Nature *444*, 387–390.
46. Reck-Peterson, S.L., Yildiz, A., Carter, A.P., Gennerich, A., Zhang, N., and Vale, R.D. (2006). Single-molecule analysis of dynein processivity and stepping behavior. Cell *126*, 335–348.
47. Beuwer, M.A., Prins, M.W.J., and Zijlstra, P. (2015). Stochastic protein interactions monitored by hundreds of single-molecule plasmonic biosensors. Nano Lett. *15*, 3507–3511.
48. Vlijm, R., Smitshuijzen, J.S.J., Lusser, A., and Dekker, C. (2012). NAP1-assisted nucleosome assembly on DNA measured in real time by single-molecule magnetic tweezers. PLoS One *7*, 1–11.
49. Harada, B.T., Hwang, W.L., Deindl, S., Chatterjee, N., Bartholomew, B., and Zhuang, X. (2016). Stepwise nucleosome translocation by RSC remodeling complexes. eLife *5*, 1–20.
50. Isojima, H., Iino, R., Niitani, Y., Noji, H., and Tomishige, M. (2016). Direct observation of intermediate states during the stepping motion of kinesin-1. Nat. Chem. Biol. *12*, 290–297.
51. Eeftens, J.M., Bisht, S., Kerssemakers, J., Kschonsak, M., Haering, C.H., and Dekker, C. (2017). Real-time detection of condensin-driven DNA compaction reveals a multistep binding mechanism. EMBO J *36*, e201797596.
52. Loeff, L., Brouns, S.J.J., and Joo, C. (2018). Repetitive DNA reeling by the Cascade-Cas3 complex in nucleotide unwinding steps. Mol. Cell *70*, 1–10.

53. Little, M.A., and Jones, N.S. (2011). Generalized methods and solvers for noise removal from piecewise constant signals. I. Background theory. Proc. R. Soc. A: Math. Phys. Eng. Sci. https://doi.org/10.1098/rspa.2010.0671.

54. Heerema, S.J., Schneider, G.F., Rozemuller, M., Vicarelli, L., Zandbergen, H.W., and Dekker, C. (2015). 1/F noise in graphene nanopores. Nanotechnology *26*, 074001.

55. Greenleaf, W.J., Woodside, M.T., and Block, S.M. (2007). High-resolution, single-molecule measurements of biomolecular motion. Annu. Rev. Biophys. Biomol. Struct. *36*, 171–190.

56. Moffitt, J.R., Chemla, Y.R., Smith, S.B., and Bustamante, C. (2008). Recent advances in optical tweezers. Annu. Rev. Biochem. *77*, 205–228.

57. Dekking, F.M. (2005). A Modern Introduction to Probability and Statistics: Understanding Why and How. (Springer Science & Business Media).

58. Li, C.B., Ueno, H., Watanabe, R., Noji, H., and Komatsuzaki, T. (2015). ATP hydrolysis assists phosphate release and promotes reaction ordering in F1-ATPase. Nat. Commun. https://doi.org/10.1038/ncomms10223.

59. Restrepo-Pérez, L., Huang, G., Bohländer, P.R., Worp, N., Eelkema, R., Maglia, G., Joo, C., and Dekker, C. (2019). Resolving chemical modifications to a single amino acid within a peptide using a biological nanopore. ACS Nano. https://doi.org/10.1021/acsnano.9b05156.

Patterns, Volume *2*

Supplemental information

AutoStepfinder: A fast and automated step detection method for single-molecule analysis

Luuk Loeff, Jacob W.J. Kerssemakers, Chirlmin Joo, and Cees Dekker

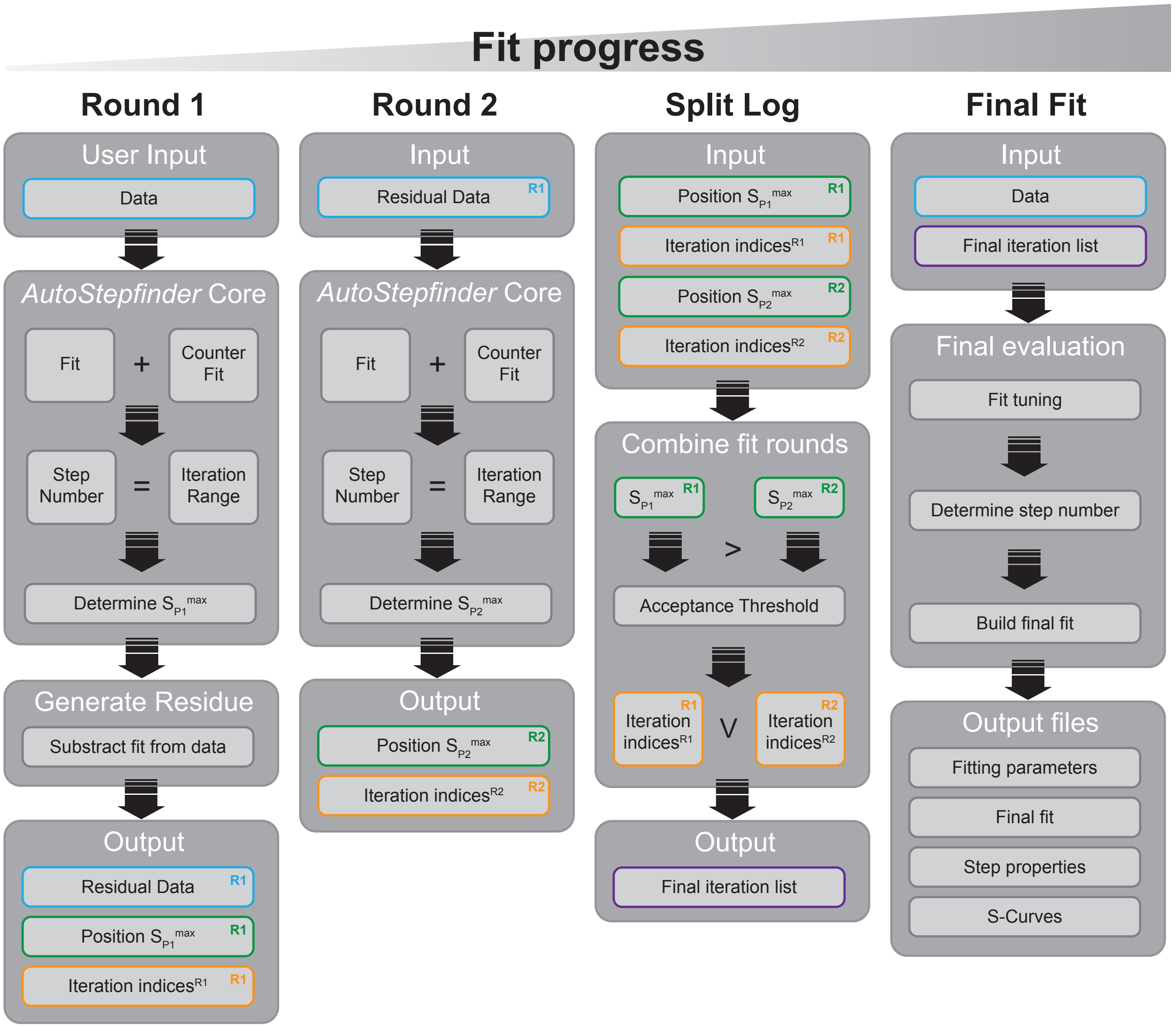

Figure S1: Flow diagram of *AutoStepfinder*

Overall layout of *AutoStepfinder* algorithm. After loading data, the *AutoStepfinder* core executes a series of partition events in which the variance between the fit and the data is minimized. This iterative process of partitioning existing plateaus continues until *AutoStepfinder* executes the maximum number of iterations. After this first round of fitting, *AutoStepfinder* determines the optimal for the data by determining the global maximum of the S-curve (S_{P1}^{max}) and saves the indices of all plateaus in the optimal fit. Subsequently, *AutoStepfinder* subtracts the fit from the data and repeats this step-fitting procedure on the residual data (Round 2). After “Round two”, *AutoStepfinder* enters the “Split Log” stage of the fitting process. In the split log stage *AutoStepfinder* determines if the S_{max} of the first and second round of fitting are above the acceptance threshold and generates a final iteration list. This final iteration list is then used to build the final fit, resulting in multiple output files.

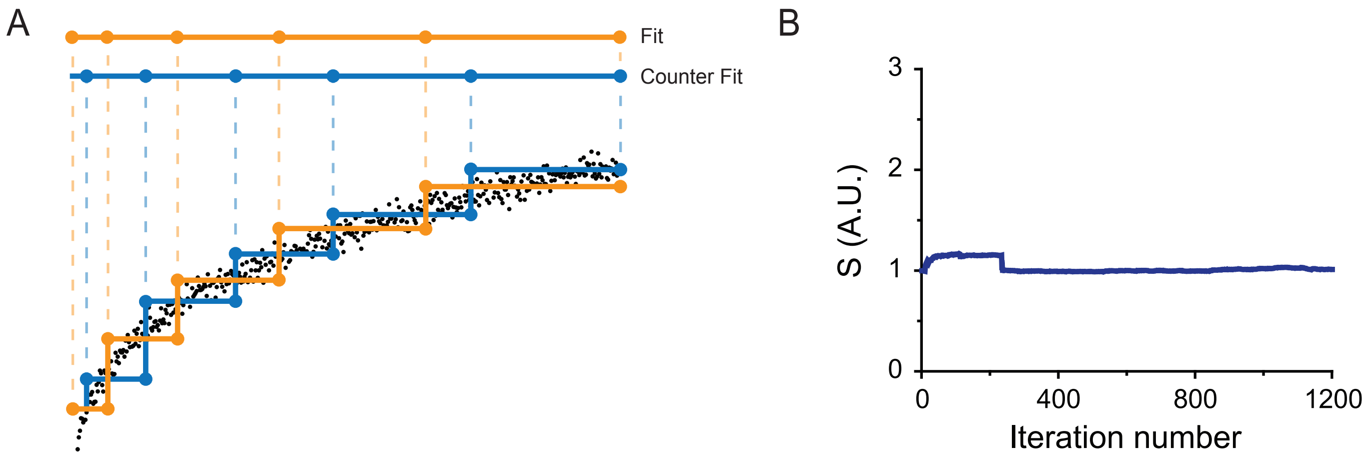

Figure S2: Step-detection in trajectories without steps

(A) Example of a step-fit on a trajectory that does not display step-like behavior. The fit is highlighted in orange line, whereas the additional counter fit is highlighted in blue. Because the data does not display step-like behavior, both the existing fit and counter fit have similar variance. **(B)** A representative example of an S-curve for data that does not exhibit steps. The S value can be calculated by taking the variance of the fit and dividing it by the variance of the counter fit. When the data does not display a step-like behavior, both the existing fit and counter fit have similar variance values, resulting in an S-value close to 1.

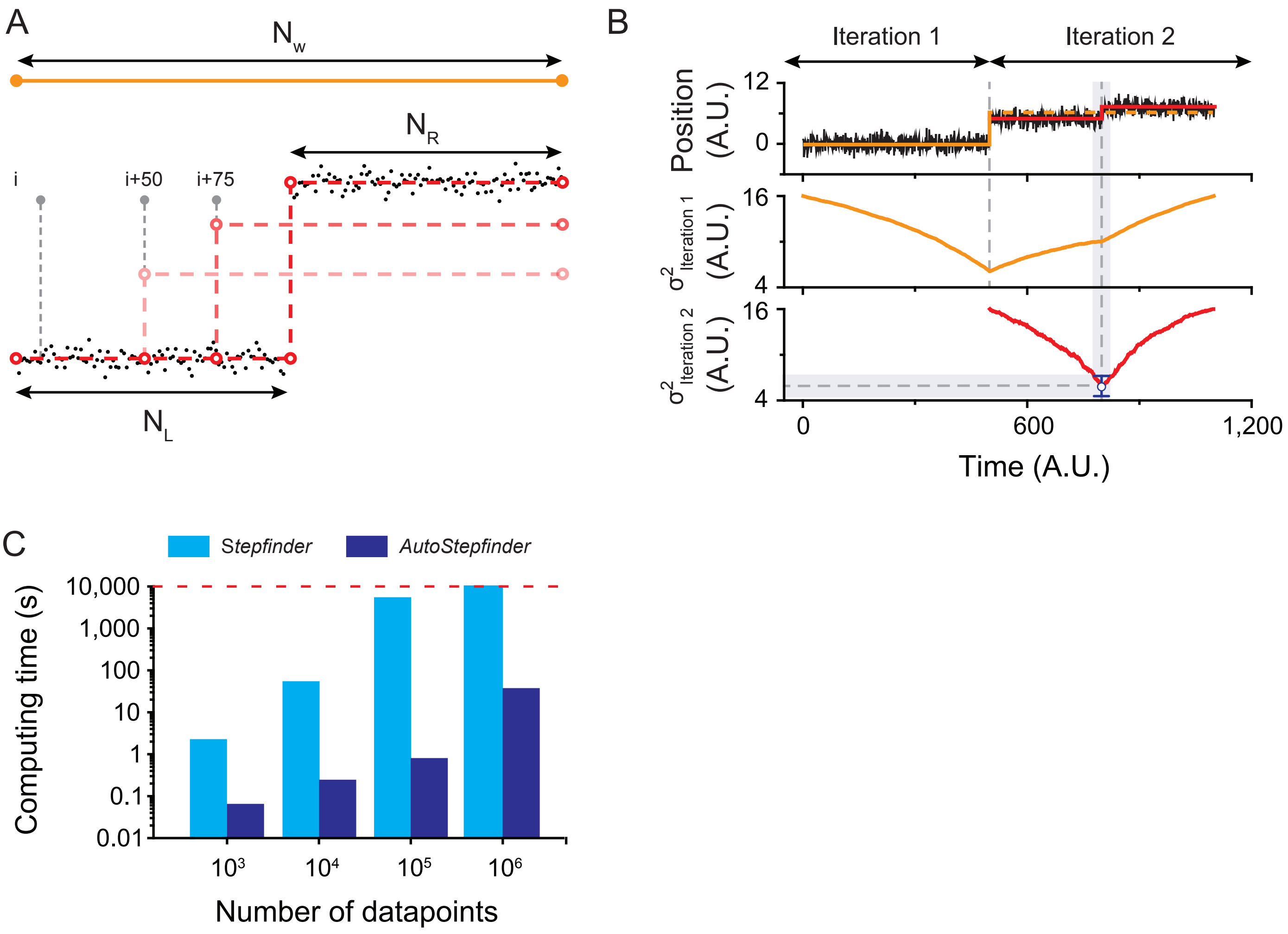

Figure S3: *AutoStepfinder* for step detection in large datasets

(A) An example of the iterative nature of the step fit procedure. The existing plateau (N_W, orange line) is partitioned into two new plateaus (N_L and N_R, dark red dashed line) at a point that yields the largest reduction in the variance. To determine this partition point, the algorithm recalculates the variance for each data point, starting at i until all data points of N_W have been calculated (e.g. i+50, faded red dashed lines). **(B)** Variance landscapes for iterative step-fitting by *AutoStepfinder*. Horizontal and vertical grey shaded areas indicate the bootstrapped step error and time error, respectively. **(C)** Comparison between *Stepfinder*[43] and the *AutoStepfinder* algorithm. The algorithms were tested by measuring the computing time of various datasets on a desktop computer, with default settings of the algorithms. The red dashed line indicates the limit (10,000 sec) that was set for the computing time.

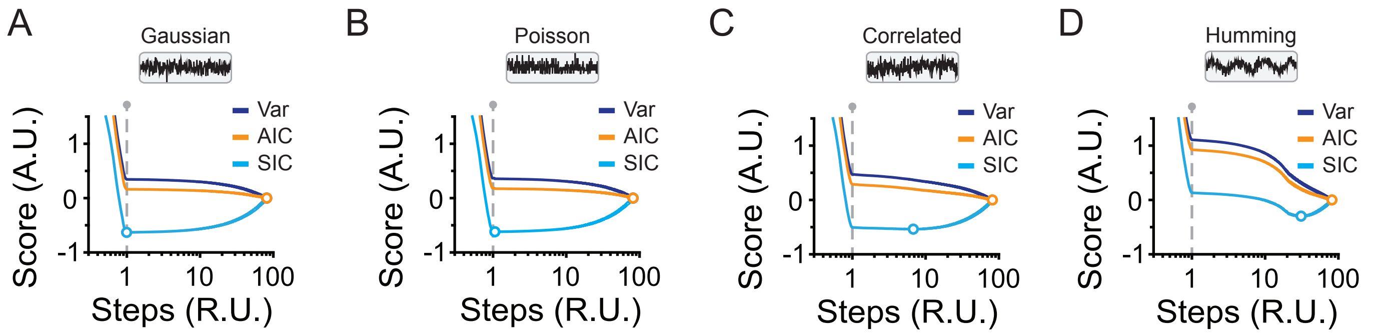

Figure S4: Step-detection with information criteria-based algorithms

(A-D) Step detection on idealized trajectories (see Figure 6a) using the variance (Var), Akaike information criterion (AIC) and Schwarz information criterion (SIC). The idealized trajectories were exposed to distinct noise types: Gaussian noise [a], Poissonian noise [b], correlated noise [c] and humming noise [d]. Each of these trajectories were exposed to noise with a SD=2.0. The dashed grey line indicates the optimal number of steps in the data, normalized at 1.0. The circles indicate the minimum of the respective information criterion.

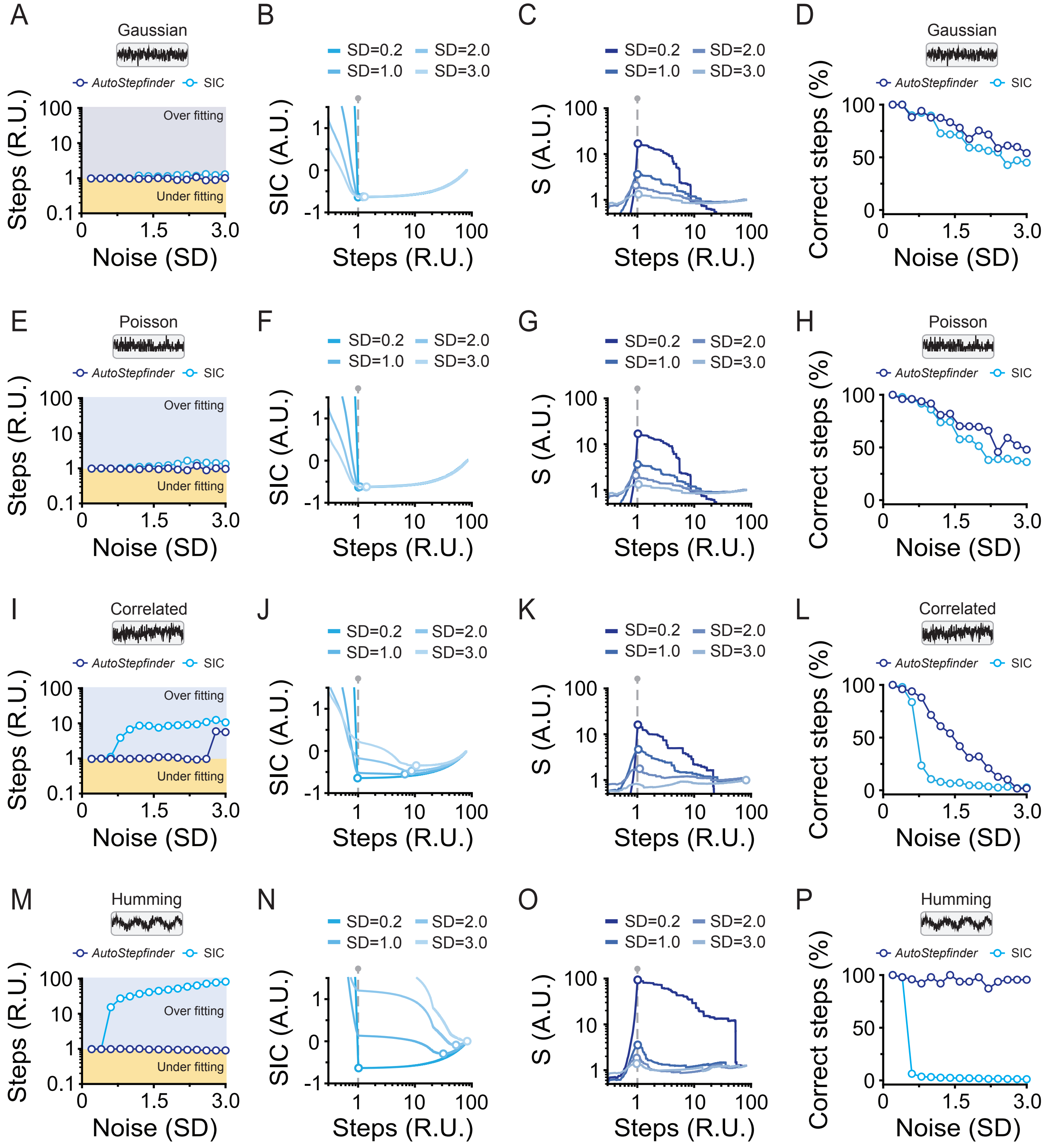

Figure S5: Robustness of *AutoStepfinder* and a SIC based algorithm

(A) Number of steps detected by *AutoStepfinder* and a SIC based algorithm on idealized trajectories (see Figure 6a) exposed to Gaussian noise (inset) with a standard deviation of SD. **(B)** SIC curves of idealized trajectories with Gaussian noise. The dashed grey line indicates the optimal number of steps in the data, normalized at 1.0. The circles indicate the minimum of the SIC curve. **(C)** S-curves of idealized trajectories with Gaussian noise. The dashed grey line indicates the optimal number of steps in the data, normalized at 1.0. The circles indicate the maximum of the S-curve. **(D)** Number of correctly identified steps by the SIC and *AutoStepfinder* algorithm trajectories with Gaussian noise (inset) with a standard deviation of SD. **(E)** Number of steps detected by *AutoStepfinder* and a SIC based algorithm on idealized trajectories (see Figure 2a) exposed to Poissonian noise (inset) with a standard deviation of SD. **(F)** SIC curves of idealized

trajectories exposed Poissonian noise. The dashed grey line indicates the optimal number of steps in the data, normalized at 1.0. The circles indicate the minimum of the SIC curve. **(G)** S-curves of idealized trajectories exposed to Poissonian noise. The dashed grey line indicates the optimal number of steps in the data, normalized at 1.0. The circles indicate the maximum of the S-curve. **(H)** Number of correctly identified steps by the SIC and *AutoStepfinder* algorithm trajectories exposed to Poissonian noise (inset) with a standard deviation of SD. **(I)** Number of steps detected by *AutoStepfinder* and a SIC based algorithm on idealized trajectories (see Figure 4a) exposed to correlated noise (inset) with a standard deviation of SD. **(J)** SIC curves of idealized trajectories with correlated noise. The dashed grey line indicates the optimal number of steps in the data, normalized at 1.0. The circles indicate the minimum of the SIC curve. **(K)** S-curves of idealized trajectories with correlated noise. The dashed grey line indicates the optimal number of steps in the data, normalized at 1.0. The circles indicate the maximum of the S-curve. **(L)** Number of correctly identified steps by the SIC and *AutoStepfinder* algorithm trajectories exposed to correlated noise (inset) with a standard deviation of SD. **(M)** Number of steps detected by *AutoStepfinder* and a SIC based algorithm on idealized trajectories (see Figure 2a) exposed to humming noise (inset) with a standard deviation of SD. **(N)** SIC curves of idealized trajectories with humming noise. The dashed grey line indicates the optimal number of steps in the data, normalized at 1.0. The circles indicate the minimum of the SIC curve. **(O)** S-curves of idealized trajectories with Humming noise. The dashed grey line indicates the optimal number of steps in the data, normalized at 1.0. The circles indicate the maximum of the S-curve. **(P)** Number of correctly identified steps by the SIC and *AutoStepfinder* algorithm trajectories exposed to humming noise (inset) with a standard deviation of SD.

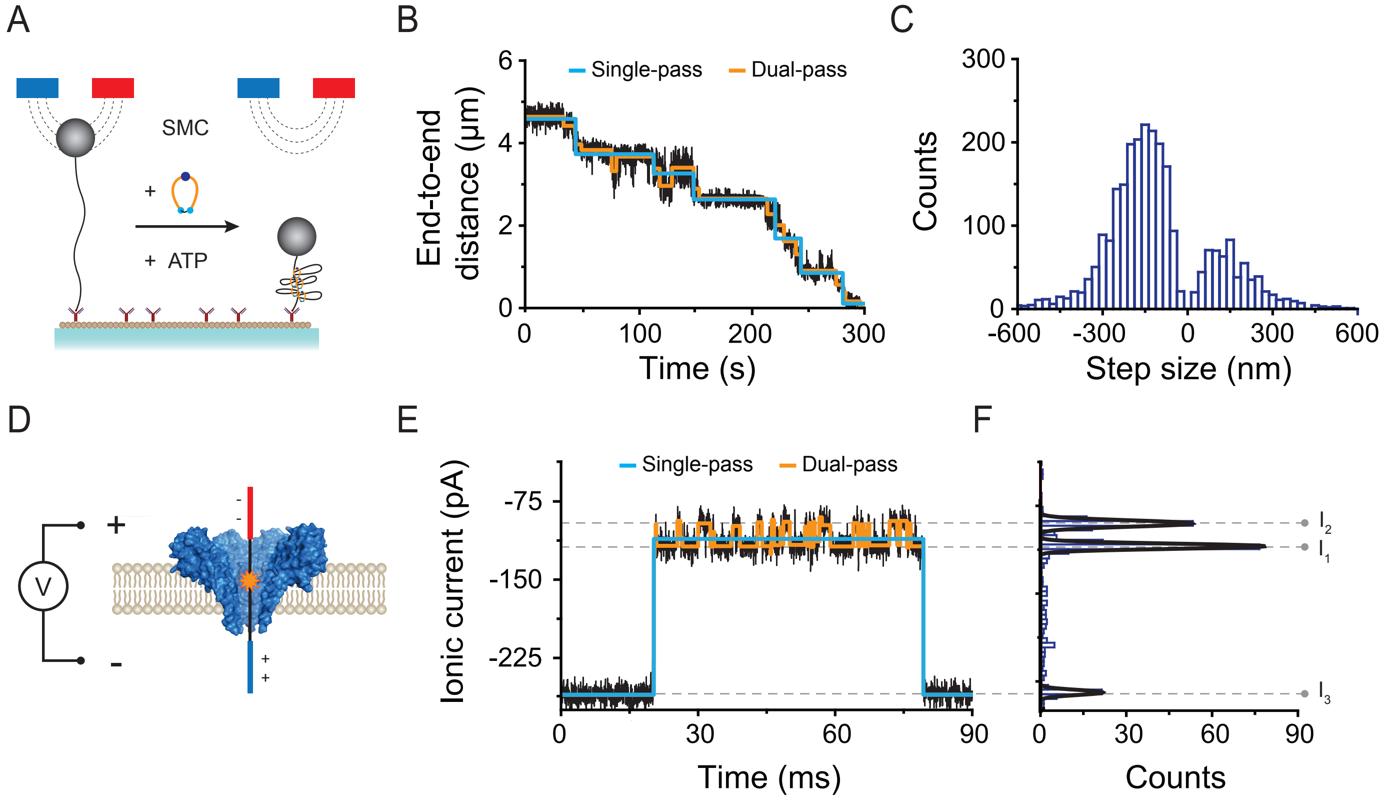

Figure S6: Step detection in magnetic tweezer and nanopore data

(A) Schematic representation of a magnetic tweezer experiment to visualize DNA compaction by condensin (SMC) proteins. A DNA molecule is tethered between a glass slide and a magnetic bead. When condensin and ATP are added, the end-to-end length of the DNA decreases. For a detailed description of these experiments see Eeftens et al.[51]. **(B)** Representative time trajectory displaying step-wise compaction by condensin (black), fitted with the *AutoStepfinder* algorithm over two rounds, single-pass (cyan) dual-pass (orange). **(C)** Distribution of step-sizes in condensin compaction experiments, obtained through the AutoStepfinder algorithm. **(D)** Schematic of a biological nanopore translocating a labelled peptide[59]. **(E)** Representative time trajectory displaying dynamics of a labelled peptide translocating through a biological nanopore (black), ffitted with the *AutoStepfinder* algorithm over two rounds, single-pass (cyan) dual-pass (orange). **(F)** Distribution of blockade levels (I) obtained through the *AutoStepfinder* algorithm. Black lines represent a Gaussian fit.

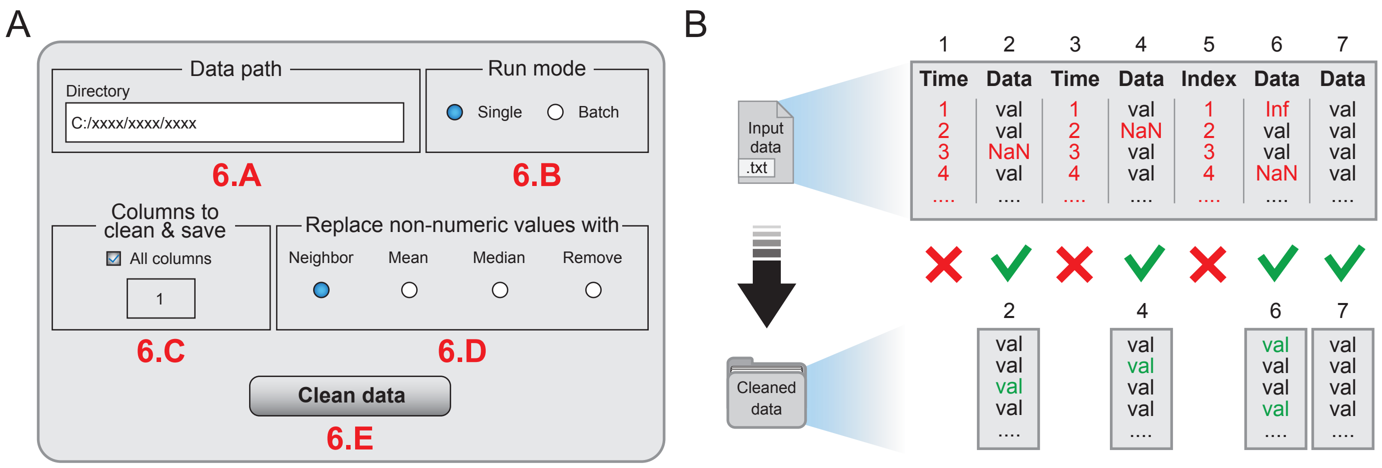

Figure S7: Graphical user interface of *DataDuster*

(A) Schematic of the graphical user interface of *DataDuster*. Red numbers correspond to the steps in the user manual that describe the function of each parameter. **(B)** Workflow of *DataDuster*. When a multi-column .txt file is loaded into *DataDuster*, *DataDuster* will clear each column from NaN and Inf values. Moreover, *DataDuster* will detect and remove the columns that increase uniformly (e.g. time and index access). *DataDuster* will export each column as a separate .txt file that is compatible with *AutoStepfinder*.

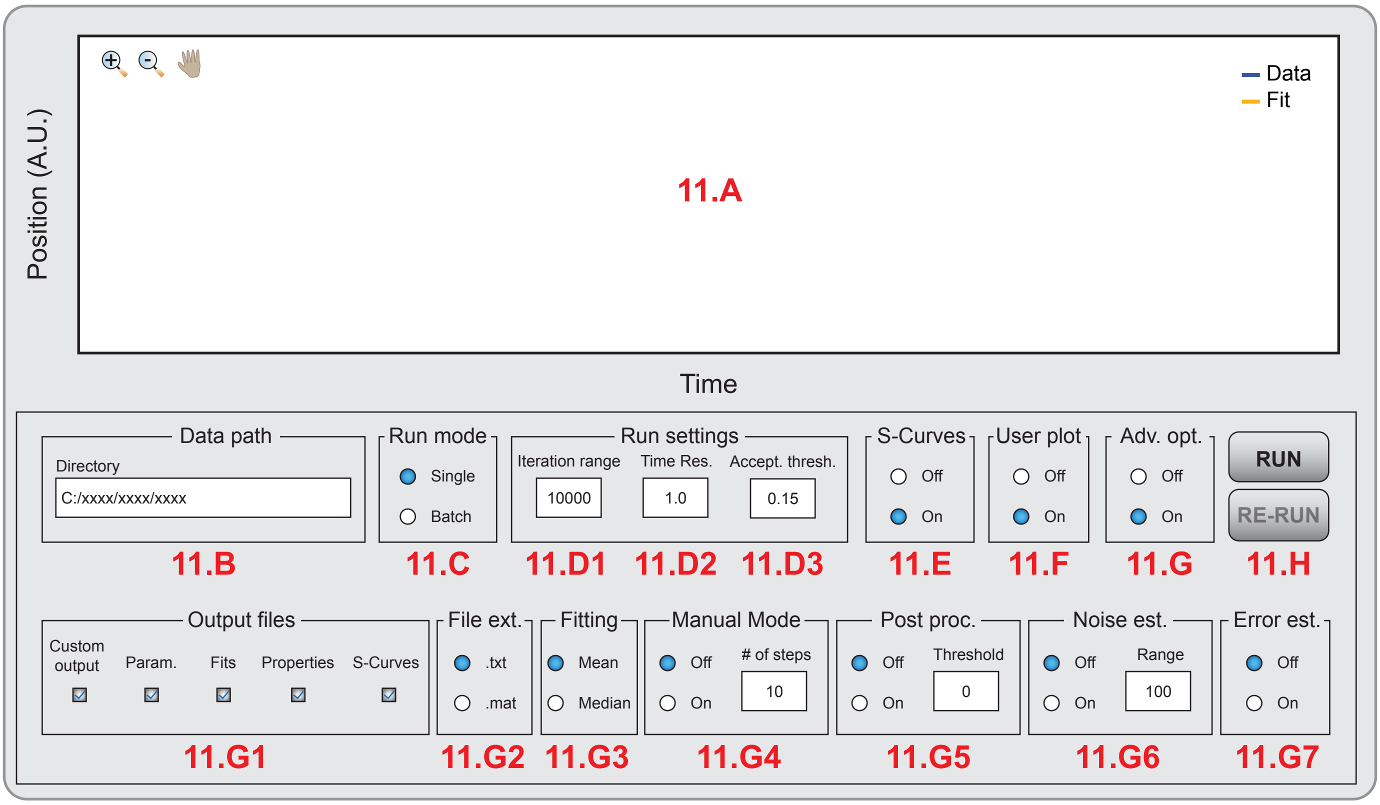

Figure S8: Graphical user interface of *AutoStepfinder*

Schematic of the graphical user interface of AutoStepfinder. The red numbers correspond to the steps in the user manual that describe the function of each parameter.

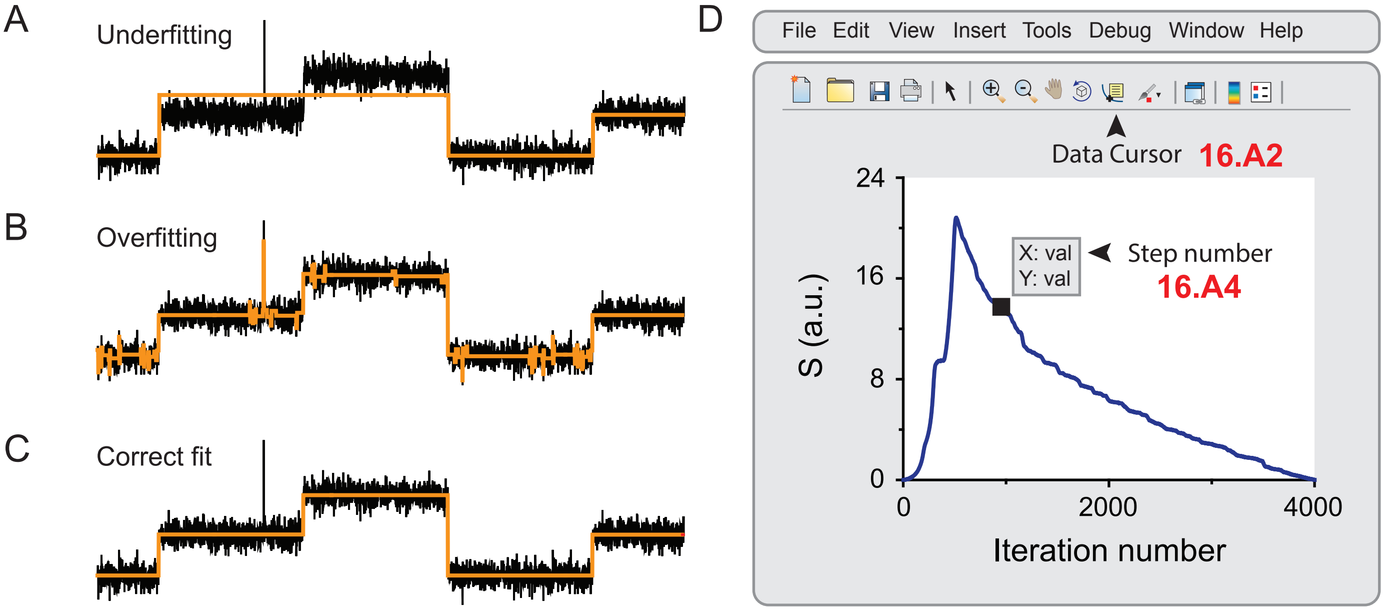

Figure S9: Inspecting the fit of *AutoStepfinder*

(A) Section of an idealized trajectory that is underfitted by *AutoStepfinder*. **(B)** Section of an idealized trajectory that is overfitted by *AutoStepfinder*. **(C)** Section of an idealized trajectory that is correctly fitted by *AutoStepfinder*. **(D)** Schematic of the S-curve window. The data cursor tool can be used to select a feature in the S-curve. The X value represents the step number that can be used in Manual Mode of *AutoStepfinder*. The red number Red numbers correspond to the steps in the user manual that describe the function of each parameter.

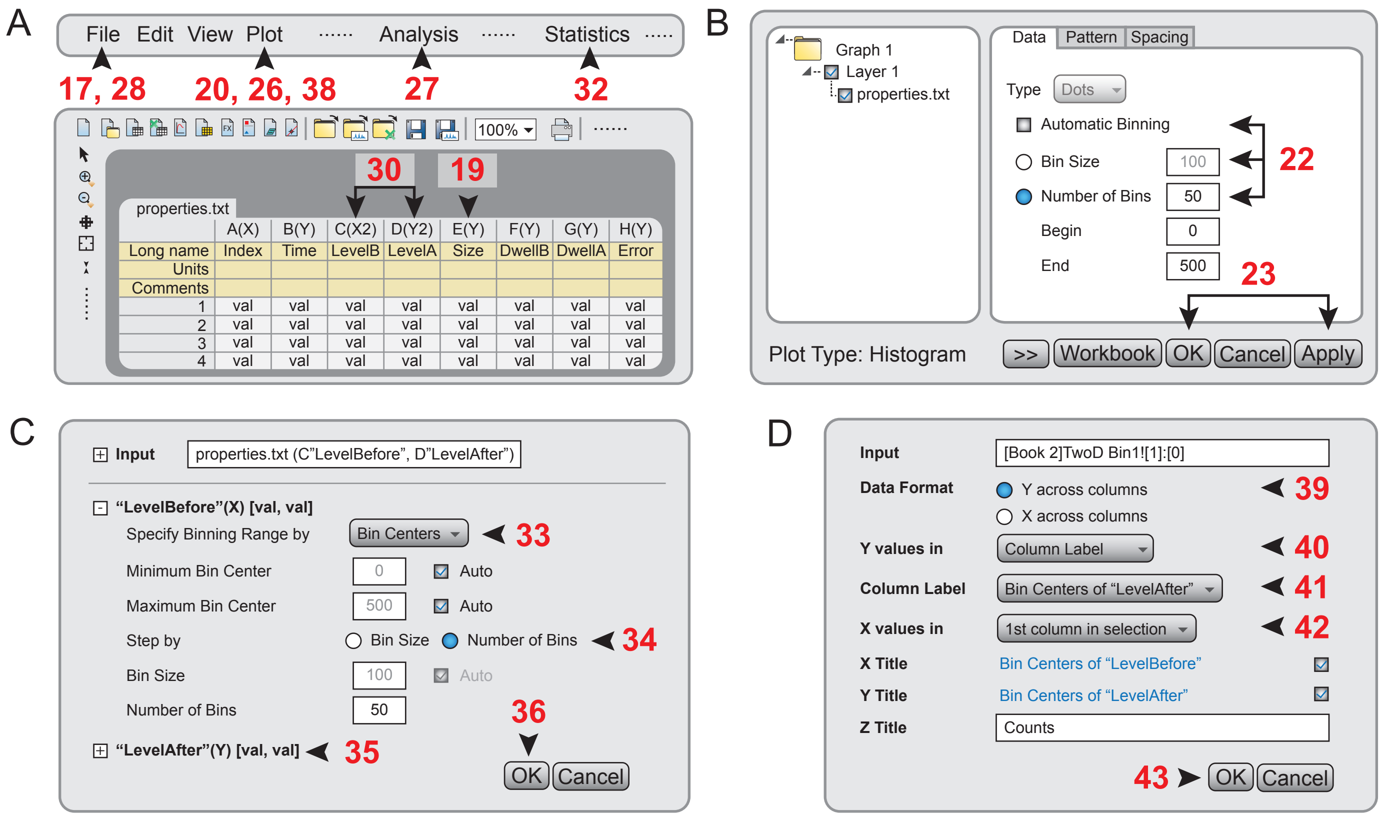

Figure S10: Using the output of *AutoStepfinder* to generate informative plots

(A) Schematic of the main window of OriginPro. Red numbers correspond to the steps in the user manual that describe the function of each parameter. **(B)** Schematic of the histogram binning window in OriginPro. The red numbers correspond to the steps in the user manual that describe the function of each parameter. **(C)** Schematic of the histogram 2D binning window in OriginPro. The red numbers correspond to the steps in the user manual that describe the function of each parameter. **(D)** Schematic of the contour plotting window in OriginPro. The red numbers correspond to the steps in the user manual that describe the function of each parameter.

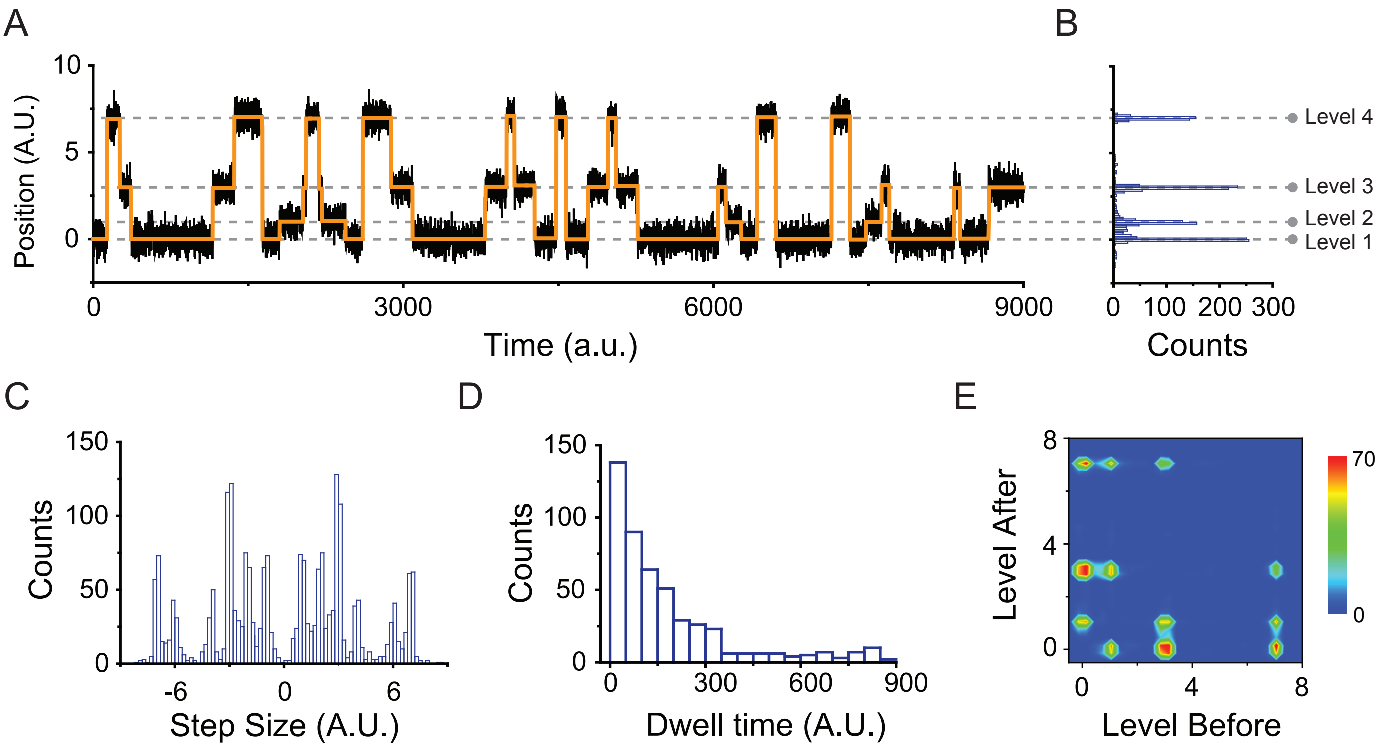

Figure S11: Examples of post-processed *AutoStepfinder* results

(A) Section of an idealized trajectory (black) that is fitted by AutoStepfinder (orange). **(B)** Distribution of levels obtained through the *AutoStepfinder* algorithm. Histogram was obtained by binning and plotting the 'Levels After' column of the properties.txt output file. **(C)** Distribution of step-sizes obtained through the *AutoStepfinder* algorithm. Histogram was obtained by binning and plotting the Step Size column of the properties.txt output file. **(D)** Distribution of dwell times obtained through the *AutoStepfinder* algorithm. Histogram was obtained by binning and plotting the Dwell Time after column of the properties.txt output file. **(E)** Transition density plot obtained through the *AutoStepfinder* algorithm. The Transition Density plot was obtained by 2D binning the Level Before and Level After columns in the properties.txt output file, followed by the generation of a contour plot.

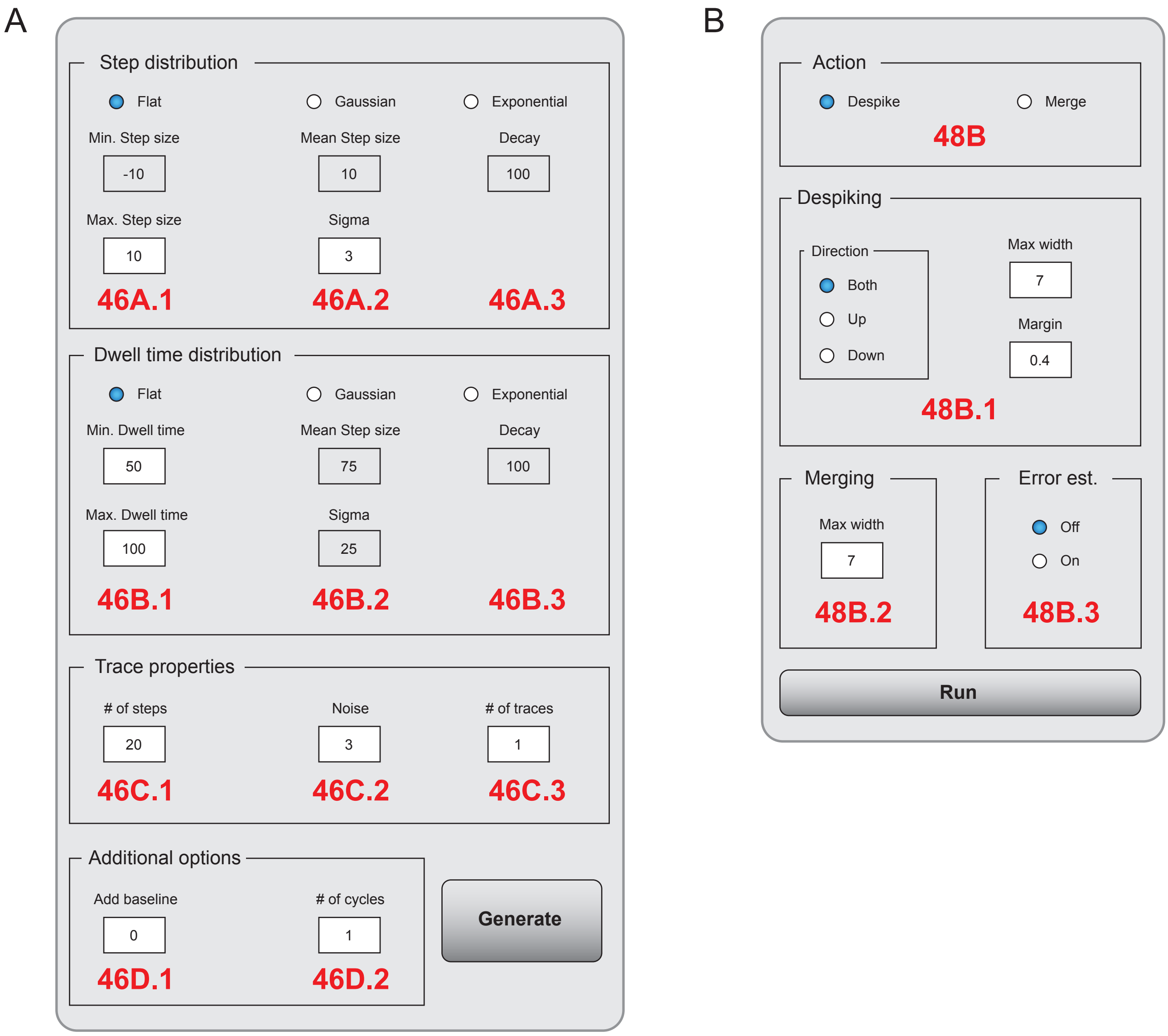

Figure S12: Auxiliary tools in the *AutoStepfinder* package

(A) Schematic of the graphical user interface of *StepMaker*. The red numbers correspond to the steps in the user manual that describe the function of each parameter. **(B)** Schematic of the graphical user interface of *StepMerger*. The red numbers correspond to the steps in the user manual that describe the function of each parameter.

Supplemental Experimental Procedures

Step by step user guide for *AutoStepfinder*

Materials

- A standard PC or Mac suitable for MATLAB with minimum requirements:
    - PC: Windows XP and higher operating system, Processor: any Intel or AMD x86 processor supporting SSE2, Disk space: 2-4 GB, RAM: 2 GB, Graphics: No specific graphics card is recommended.
    - Mac: Mac OS X 10.9.5 or higher operating system, Processor: all Intel-based Macs with an Intel core 2 or later, Disk space: 2-4 GB, RAM: 1 GB, Graphics: No specific graphics card is recommended.
- MathWorks MATLAB version 2015a or above (http://www.mathworks.com)
    - MathWorks MATLAB Database Toolbox
- The *AutoStepfinder* package (the most up-to-date version is available at: http://www.ceesdekkerlab.nl and http://www.chirlmin.org.
- Optional: Data analysis and graphing software to post-process data (e.g. OriginLab Origin (http://www.originlab.com/), Graphpad Prism (http://www.graphpad.com/) or Microsoft Office Exel (http://www.microsoft.com/).

Procedure

General notes on experimental data for optimal step fitting

AutoStepfinder is capable of detecting steps in trajectories of various techniques, including single-molecule fluorescence, nanopores, and magnetic and optical tweezers. While the details of these experimental approaches differ, we provide a set of general guidelines that will maximize the performance of *AutoStepfinder*.

I. **Sampling rate:** *AutoStepfinder* determines the significance of steps based on the number of data points in the plateau (N_i) and the size of the step (Δ) (Figure 2). Thereby, the sampling rate which the single-molecule measurement is performed, i.e. the number of data points per time-unit, is an important factor in step fitting. To facilitate step fitting by *AutoStepfinder*, it is recommended to maximize the number of independent data points per plateau by acquiring data at high sampling rates. *Important:* Increasing the sampling rate in single-molecule measurements may come at a cost. For example, an increased

sampling rate in single-molecule fluorescence measurements will require higher laser powers to collect a large number of photons per frame. These high laser powers will induce fast photobleaching and thereby limit the observation time of the experiment. In addition, a similar upper sampling limit exists for response time-limited systems, such as in magnetic/ optical tweezer and nanopore experiments.

II. **Drift:** A commonly found artefact in single-molecule trajectories is drift or other types of movement in the x-, y- or z-direction. These movements result in trajectories with a gradually decreasing or oscillating signal, which may interfere with the performance of *AutoStepfinder*. Therefore, it is recommended to limit drift during the measurements as much as possible and discard traces from the analysis that show an excessive amount of drift.

III. **Filtering of data:** In single-molecule data analysis, it is a common practice to reduce the noise in the trajectories by smoothing the data with moving averages and filters. However, the use of these filters may also smooth the state-to-state transitions. Given that *AutoStepfinder* works best on instant state-to-state transitions, care should be taken when applying such filters. Therefore, it is advised to run *AutoStepfinder* on raw data or otherwise consider using a step preserving filter, such as a median or Chung-Kennedy filter.

Initializing the *AutoStepfinder* algorithm and auxiliary tools

1. Start MATLAB as described by Mathworks.
2. Copy all the files enclosed in *AutoStepfinder* folder to the working directory of MATLAB. Alternatively, change the initial working directory of MATLAB by going Home tab then Preferences > General > "Initial working folder" and specify the full path to the *AutoStepfinder* folder.

Formatting data for step detection by *AutoStepfinder*

3. *AutoStepfinder* runs on a single-column text file (.txt) that encompasses numeric single-molecule data. Alternatively, *AutoStepfinder* can run two-column text files (.txt) with the time axis in the first column and data in the second column. In the latter case, the time axis will be ignored during the step-fitting procedure.
4. To ensure *AutoStepfinder* runs properly, the input files for *AutoStepfinder* should be free of non-numeric values, including: ‘infinity values’ (Inf) and ‘not a number’ (NaN).

To remove these values, one could use the *DataDuster* auxiliary tool, which is located in the *AutoStepfinder* package.

5. Start the *DataDuster* auxiliary tool by opening DataDuster.m and running the code in the editor tab or the command window of MATLAB.
6. After pressing “Run”, a graphical user interface (GUI) will appear, in which the user can adjust the run settings for *DataDuster* (Figure S7).

   For troubleshooting, see table S2

   6A. *Data Path*: The Data Path box allows one to specify the directory of the input data. *Important:* By default, the directory is set to the current directory of MATLAB. The default location of Data Path can be changed at line 47 of the DataDuster.m file.

   6B. *Run Mode*: Run mode specifies whether one runs *DataDuster* on a single file or run all files in a selected folder. To run a single .txt file, check single and run the algorithm to select a file. For batch style processing, check batch and *DataDuster* will analyze all .txt files in the specified directory.

   6C. *Columns to clean and save*: The columns to clean and save box allows one to specify on which column to run *DataDuster.* By default, it runs through all columns in the loaded .txt file(s). To specify a specific column, uncheck the “all columns” box and specify the number of the column to analyze.

   6D. *Replace non-numeric values with*: The replace non-numeric values with box allows one to specify what to do with the non-numeric values.

   6.D1. *Neighbor*: Replaces non-numeric values with the mean value of the neighboring two data points.

   6.D2. *Mean*: Replaces non-numeric values with the mean value of the dataset.

   6.D3. *Median*: Replaces non-numeric values with the median value of the dataset.

   6.D4. *Remove*: Removes non-numeric values from the dataset.

   6E. *Clean data:* The clean data button initiates *DataDuster* data cleaning procedure.

7. To start the data cleaning procedure, press the “Clean data” button, located on the bottom of the GUI (Figure S7A).
8. Browse to the directory of interest and select the file (single run) or the folder (batch run) that encompasses the single-molecule data for data cleaning.

9. The output of the *DataDuster* is saved in a new folder called "cleaned_data_method", where method refers to the input of the "replace non-numeric values with" box. This folder is generated in the directory of the input file. If a multi-column .txt file was loaded, *DataDuster* will output each column as a separate .txt file named: filename_col_0x.txt, which can be directly loaded into the *AutoStepfinder* algorithm. Notably, the MATLAB console will display the number of replaced values in each column. *Important:* *DataDuster* does not export trajectories that exhibit equidistant increase in signal, e.g. the time axis of trajectories or indices, as these trajectories are featureless and will not result in step detection by the *AutoStepfinder* algorithm.

Startup and Graphical user interface of *AutoStepfinder*

10. Start *AutoStepfinder* by opening AutoStepfinder.m and running the code in the editor tab or the command window of MATLAB.
11. After pressing "Run", a GUI will appear, in which the fitting procedure will be executed and fitting parameters can be adjusted (Figure S8).

    11A. *Fitting Window*: The top half of the GUI comprises a fitting window. This window allows one to visually inspect the fit after executing the *AutoStepfinder* algorithm. The data is displayed in blue and the corresponding fit in orange.

    11B. *Data Path*: The Data Path box allows one to specify the directory of the input data. *Important:* By default, the directory is set to the current directory of MATLAB. The default location of Data Path can be changed at line 38 of the AutoStepfinder.m file.

    11C. *Run Mode*: Run mode specifies whether one runs *AutoStepfinder* on a single file or run all files in a selected folder. To run a single .txt file, check single and run the algorithm to select a file. For batch style processing, check batch and *AutoStepfinder* will analyze all .txt files in the specified directory.

    11D. *Run Settings*: The Run Settings box provides a minimal set fitting of parameters that allows one to tune the fitting procedure (Figure S8).

    11.D1. *Iteration range*: The iteration range parameter determines to what extent *AutoStepfinder* continues the fitting procedure. Once the number defined by the

iteration range is found, the algorithm stops partitioning plateaus to minimize χ^2 and determines the optimal fit. For datasets with limited step numbers, the iteration range parameter can be decreased to reduce the computing time of the fitting procedure. Typically, the initial iteration range is set to ¼ of the number of data points of the input data.

11.D2. *Time resolution*: The time resolution parameter corresponds to the temporal resolution (e.g. the time interval between each data point) of the measurement. This parameter will be used for the time data in the output files of *AutoStepfinder*. Important: If a file with two columns is provided, time and data, the time column (first column) is ignored by the *AutoStepfinder* algorithm.

11.D3. *Accept(ance) thresh(old)*: The acceptance threshold sets a threshold for each fitting round and is compared to S^{max}-1. If the S-curve of the first or second fitting round provides a S^{max} that lays below the threshold, this fitting round will not be executed. Typically, the acceptance threshold ranges between: 0.1 – 1. *Important:* Care should be taken when adjusting the acceptance threshold. If the acceptance threshold is set above the S^{max} of the first round of fitting, *AutoStepfinder* will not execute the step-finding procedure.

11E. *S-Curves*: When S-Curves are turned on, the *AutoStepfinder* will display the S-curves of the first and second round in a separate window.

11F. *User plot*: The user plot box allows one to quickly assess the fitting result of the *AutoStepfinder* algorithm. By turning the *User plot* function on, *AutoStepfinder* will plot the step size, step levels and dwell-time histograms in a separate window.

11G. *Adv(anced) Options*: Enabling the advanced option box displays the advanced setting of *AutoStepfinder* (Figure S8). *Important:* It is noted that these settings are intended for advanced users that have full understanding on the step fitting procedure.

11.G1. *Output files:* The output files box allows one to select which output files *AutoStepfinder* saves. By saving a subset of output parameters, additional speed can be gained, which may be preferred for large datasets or when optimizing fitting settings.

11.G2. *File ext(ension)*: The file extension box allows one to select the file type *AutoStepfinder* outputs. When .txt is checked, *AutoStepfinder* outputs text files. If .mat is checked, *AutoStepfinder* outputs matlab files, which can be used to further post-process the output of *AutoStepfinder* in Matlab.

11.G3. *Fitting*: The fitting box allows one to choose how the position of the plateaus of the final fit is determined. By default, *AutoStepfinder* uses the averages of each plateau to determine its position for each iteration and the final fit. However, in some cases (e.g. when data exhibits spikes), one may choose to build the final fit using the median of each plateau, by checking the median parameter.

11.G4. *Manual mode*: Manual mode allows one to define the number of steps that have to be found by the algorithm. Typically, the number of manually fitted steps does not exceed the iteration range. *Important:* Manual mode overrides the quality assessment of *AutoStepfinder* and should only be used in an informed manner.

11.G5. *Post proc(essing)*: The post processing box allows one to discard the baseline plateaus from the fit. All fitted plateaus that have a value below the provided threshold in units of the input data are removed from the "filename_properties" output file.

11.G6. *Noise estimation*: The noise estimation box allows the user to perform pairwise distance noise estimation on the residual noise in the data (data with the fit substracted). By default, the range over which the noise estimation is performed is set to 100 data points. By enabling the *Noise estimation* function, AutoStepfinder opens a new window with curves that correspond to the Residual noise (blue line), the median (red line) and the pairwise distance noise (red circle). *Important:* For low pass filtered data the pairwise distance error may be under estimated, in this case it is advised the median value as noise estimate.

11.G7. *Error estimation*: The error estimation box allows the user to perform bootstrap analysis that provides the 95% confidence intervals of both the step-sizes and the plateaus. When error estimation is enabled, the *AutoStepfinder* output includes two additional columns with the respective 95% confidence intervals.

11H. *Run and Re-run*: The run button initiates *AutoStepfinder* step fitting procedure. The Re-run button enables after a single run has been executed and allows the user to re-analyze the data without having to load the data.

Running *AutoStepfinder*

12. To start the step-finding procedure press the “Run” button, located on the right side of the GUI (Figure S8).
13. Browse to the directory of interest and select the file (single run) or the folder (batch run) that encompasses the single-molecule data for *AutoStepfinder*.
14. Press open to start the step-finding procedure. The progress of the *AutoStepfinder* analysis is displayed in the console of MATLAB. Once the console indicates “done!” the fitting procedure has been completed and output files have been saved.
15. The output of the *AutoStepfinder* analysis is saved in a new folder (StepFit_Result), which is generated in the directory that is provided in the datapath box of the GUI. By default, the output of *AutoStepfinder* consists of four files: “filename_fits”, “filename_properties” and “filename_s_curve” and “filename_config”. The “filename_fits” file consists of three columns (Table S1) and can be used to plot the data with corresponding fit. The “filename_properties” file consists of 8 columns (Table S1) and encompasses the information required to generate histograms of the step size, step levels and dwell-times. The “filename_s_curve” file encompasses the information required to plot the S-curves of the first and second round (Table S1). Lastly, *AutoStepfinder* generates a “filename_config” file that encompasses all the parameters that were used to generate the fit (Table S1). Notably, when batch mode is selected, *AutoStepfinder* generates additional .JPEG files of the fit window, user plots and S-curves (Table S1).

Fine tuning the fit parameters for optimal results

16. <u>*Important:*</u> *AutoStepfinder* is a robust approach for automated step detection that determines the optimal fit based on statistical arguments. However, despite the automated detection of steps, it is advised to always carefully inspect the quality of the

fitting result before proceeding with post-processing of the data. The quality of the fit can be assessed by using the fitting window and the built-in controls of the GUI (e.g. zoom in/out and pan) (Figure S8, Step 11.A). Below we provide guidelines on how to interpret and fine-tune fitting parameters to obtain optimal fitting results. As a rule of thumb, it is recommended to maintain a conservative attitude towards step fitting in which it is better to miss small events rather than to introduce spurious steps by overfitting.

16A. *Underfitted data:* Data is considered underfitted when the number of detected steps by *AutoStepfinder* is significantly lower than the number of steps that are present in the data. Therefore, a hallmark for underfitted data is a fit in which a significant number of steps are missed. At the location where steps are obviously missed, the plateau of the fit deviates from the data (Figure S9A) and thereby these plateaus are generally associated with in large step errors (properties output file, column 8). Underfitting of data is typically associated with irregular features in the S-curve; therefore, it is recommended to inspect the corresponding S-curve (11.E). While the S-curve normally shows a sharp peak at the optimal step number, for some datasets the S-curve may have a non-canonical shape. For example, it might have a secondary peak or shoulder that represents a more realistic step number to fit.

Typically, underfitting can be prevented by adjusting the parameterization of the *AutoStepfinder* algorithm. Underfitting may occur when the final number of steps in the data is too close to the user provided iteration range (Step 11.D1) or when the S^{max} of the second round of fitting lies below the acceptance threshold (Step 11.D1). Thereby, underfitting can be prevented by increasing the iteration range or by lowering the acceptance threshold. Alternatively, one can determine the position of a specific feature in the S-curve (e.g. a shoulder or secondary peak) as follows:

16.A1. Select the data cursor tool from the build in controls of the S-curve plotting window (Figure S9D)

16.A2. Use the data cursor tool to determine the step number (X value) at which the shoulder or secondary peak in the S-curve occurs (Figure S9D).

16.A3. Enable to advanced settings and engage manual mode (Step 11.G4) under the advanced settings (Step 11.G).

16.A4. Insert step number that was determined with the data cursor tool in the manual mode box (Figure S9D).

16.A5. Run *AutoStepfinder* with manual mode engaged.

<u>*Important*</u>: By engaging manual mode *AutoStepfinder* fits the user-defined number of steps to the data, bypassing the quality assessment of the *AutoStepfinder* algorithm. Therefore, the use of manual mode should always be guided by specific features of the S-curve. It is strongly discouraged to use manual mode without a compelling rationale.

16B. *Overfitted data:* Data is considered overfitted when the number of detected steps by *AutoStepfinder* is significantly higher than the number of steps that are present in the data. Therefore, a hallmark for overfitted data is a fit in which plateaus are fitted with a significant number of small steps that follow the noise of the data (Figure S9B). By fitting the noise of the data, plateaus are divided into smaller ones, which can detrimental for the outcome of the step analysis (e.g. dwell-times can be significantly shorter when data is overfitted). In most experimental contexts, it is better to miss small events than to introduce spurious small steps by overfitting.

Overfitting of data is typically associated with wrong parameterization of the *AutoStepfinder* algorithm. Overfitting of data by *AutoStepfinder* typically occurs when the user-defined acceptance threshold is set too low. As a result of the low acceptance threshold, *AutoStepfinder* will consider noise as small steps and overfit the data (Step 11.D3). In some cases, overfitting may occur when the user provides an iteration range that is approximately more than an order of magnitude larger than the number of steps in the data, which can be prevented by lowering the iteration range (Step 11.D1).

16C. *Correctly fitted data:* A fit describes the data well when the majority of the plateaus are fitted, while noise and other artefacts in the data are not included in the fit (Figure S9C). If one is satisfied with the fitting results proceed to step 17 of this protocol.

Post-processing of *AutoStepfinder* output

The output of *AutoStepfinder* can be post-processed to generate informative plots using any kind of spreadsheet or graphing software (e.g. OriginPro, Prism, SigmaPlot, MATLAB, Python and Excel). Below we provide a description on how the data can be processed using OriginPro.

Step-size, level and dwell-time histograms

17. Open OriginPro and load the filename_properties.txt file by going to File > Import and select Single ASCII (Figure S10A).

18. Select the filename_properties.txt in the StepFit_Result folder and click “Open”.
19. Select a column of interest (e.g. column 5, StepSize) by clicking on the column header (E(Y)). The column should now be highlighted in black (Figure S10A).
20. To generate a histogram, go to Plot > Statistics and select “Histogram” (Figure S10A).
21. Double clicking on the bars of the histogram will open the Plot Details window (Figure S10B) that allows one to tune the bin size. Alternatively, right click on the bars of the histograms and select “Plot Details”.
22. Uncheck “Automatic Binning”, and define a bin size or a number of bins by selecting “Bin Size” or “Number of Bins”, respectively (Figure S10B). As a rule of thumb, one can estimate the appropriate number of bins for a dataset by taking the square root of the number of data points in the dataset (round off if necessary).
23. Once the appropriate number of bins has been determined press “Apply” and “OK” (Figure S10B). This will generate a histogram of the selected column, for example with Levels (Figure S11B), Step size (Figure S11C) or Dwell-times (Figure S11D). Notably, for step size histograms a peak at a negative step size indicates a step from a higher level to a lower level, whereas a peak at a positive step size indicates a step from a lower to a higher level (Figure S11C).
24. To fit the histograms, the histograms need to be converted to a bar plot. To convert the histogram to a bar plot, right click on the histogram and select “Go to bin worksheet”.
25. Select the “Bin Centers (X)” and “Counts (Y)” columns.
26. With the columns selected go to Plot > Column/ Bar/ Pie and select “Column” (Figure S10A).
27. This bar plot can be fitted with different functions, depending on the distribution of the data. For example, normally distributed data can be fitted with a Gauss function by going to Analysis > Peaks and Baseline > Multiple Peak Fit and selecting: “Open Dialog”, whereas data that follows an exponential decay can be fitted by going to Analysis > Fitting > Exponential Fit and selecting: “Open Dialog” (Figure S10A).

Transition density plots

28. Open OriginPro and load the filename_properties.txt file by going to File > Import and select Single ASCII (Figure S10A).
29. Select the level before (C(Y)) column.

30. Right click on the selected column and click on: Set As > X, the column header should change from C(Y) to C(X2) (Figure S10A).
31. Select the level before (C(X2)) and level after (D(Y2)) column by clicking on the column header. The column should now be highlighted in black.
32. Bin the data in 2D by going to Statistics > Descriptive Statistics > 2D Frequency Count/ Binning and select "Open Dialog" (Figure S10A).
33. Adjust "Specify Binning Range by" to "Bin Centers" (Figure S10C).
34. Uncheck "Automatic Binning", and define a bin size or a number of bins by selecting "Bin Size" or "Number of Bins" (Figure S10C). As a rule of thumb, one can estimate the appropriate number of bins for a dataset by taking the square root of the number of data points in the dataset, round off if necessary.
35. Repeat step 33-34 for the Y data, selecting the same parameters, such as bin size/ bin numbers (Figure S10C).
36. Press "OK" to generate a new workbook with 2D binned data (Figure S10C).
37. Select all columns of the newly generated workbook with 2D binned data.
38. With the columns selected go to Plot > Contour and select "Color Fill" (Figure S10A).
39. In the pop-up window select "Y across columns" for "Data Format" (Figure S10D).
40. Change "Y Values in" to "Column Label" (Figure S10D).
41. Make sure that in the "Bin Centers", "LevelAfter" is selected under "Column Label" (Figure S10D).
42. Select "1st column in selection" for "X values in" (Figure S10D).
43. Press "OK" (Figure S10D) and the transition density plot will be generated (Figure S11E).
44. The contour plot can be formatted by right clicking on the center of the graph window and going to "Plot Details".

Generating trajectories with StepMaker

StepMaker is a tool that allows a user to generate trajectories of various techniques, including single-molecule fluorescence, nanopores, and magnetic and optical tweezers. Below we provide a set of general guidelines that demonstrate how *StepMaker* can be tuned to obtain specific trajectories.

Startup and Graphical user interface of *StepMaker*

45. Start *StepMaker* by opening StepMaker.m and running the code in the editor tab or the command window of MATLAB.
46. After pressing "Run", a GUI will appear, in which the simulation procedure will be executed and parameters can be adjusted (Figure S12A).

    46A. The step distribution box allows one to select how steps are distributed in the simulated trajectory (Figure S12A). The user has the choice between:

    46A.1 *Flat distribution*: When the step distribution is flat all step sizes between the indicated minimum and maximum step size have an equal chance of occurring.

    46A.2 *Gaussian distribution*: When the step distribution is gaussian step sizes are randomly picked from a gaussian distribution with the indicated mean and sigma.

    46A.3 *Exponential distribution*: When the step distribution is exponential, steps are randomly picked from an exponential distribution with the indicated decay constant.

    46B. The dwell time distribution box allows one to select how steps are distributed in the simulated trajectory. The user has the choice between:

    46B.1 *Flat distribution*: When the dwell time distribution is flat all dwell times between the indicated minimum and maximum dwell times have an equal chance of occurring.

    46B.2 *Gaussian distribution*: When the dwell time distribution is gaussian dwell times are randomly picked from a gaussian distribution with the indicated mean and sigma.

    46B.3 *Exponential distribution*: When the dwell time distribution is exponential, dwell times are randomly picked from an exponential distribution with the indicated decay constant.

46C. The trace properties box (Figure S11A) allows the user to select the properties of the simulated trajectory:

46C.1 *# of steps*: Number of steps in the generated trajectory. Naturally, the number of dwells is one unit higher.

46C.2 *Noise*: Standard deviation of the Gaussian noise in the signal.

46D.2 *# of Traces*: Number of trajectories that are generated by *StepMaker*.

46D. Additional options (Figure S12A):

46D.1 *Add baseline*: Number of data points to be added before or after the trajectory. When the value of the baseline is negative the baseline is added to the beginning to the trajectory. When the value of the baseline is positive the baseline is added to the beginning to the trajectory.

46D.2 *# of cycles*: Number of repeats within the trajectories that are generated by *StepMaker*. For example, this option can be used to make two-state transition trajectories.

Postprocessing trajectories with StepMerger

StepMerger is a tool that allows a user to remove statistically significant features that may not of interest, such as blinking and spikes from the output of *AutoStepfinder*. Below we provide a set of general guidelines that demonstrate how *StepMerger* can be tuned to remove these features.

Startup and Graphical user interface of *StepMerger*.

47. Start *StepMerger* by opening StepMerger.m and running the code in the editor tab or the command window of MATLAB.
48. After pressing "Run", a GUI will appear, in which the merging procedure will be executed and parameters can be adjusted (Figure S12B).

    48A *Input directory:* The Input directory box (Figure S12B) allows one to specify the directory of the input data. *Important:* By default, the directory is set to the current directory of MATLAB.

    48B. *Action:* The action box allows the user to choose how the data is processed.

48B.1 *Despiking:* Despiking allows to the user to remove blinks and spikes that return to the same level from the *AutoStepfinder* output. Spikes and blinks that are within the indicated maximum width will be removed. Moreover, the margin option determines the maximum relative difference between the up and down steps that comprises the spike.

48B.2 *Merging:* Merging allows the user to merge small spurious steps that are within the indicated max width and that move after another in the same direction. These small spurious steps are typically associated with non-instantaneous steps.

48B.3 *Error estimation*: The error estimation box allows the user to perform bootstrap analysis that provides the 95% confidence intervals of both the step-sizes and the plateaus. When error estimation is enabled, the *StepMerger* output includes two additional columns with the respective 95% confidence intervals.

49. The output of the *StepMerger* has the same format as *AutoStepfinder* (the fit and properties), which are generated in the directory that is provided in the input directory box of the GUI.

Table S1| Output of the *AutoStepfinder* algorithm

File	Column	Name	Description
filename_fits	1	Time	Time axis of the dataset. *Important:* If the time resolution was not provided in the Run Settings box, the time axis is converted to indices.
	2	Data	Data that has been loaded into *AutoStepfinder*.
	3	Fit	The corresponding fit of the data that was generated by *AutoStepfinder*.
filename_properties	1	Index Step	Index based location of the step between two plateaus. The location of the step is defined by the last data point of the plateau that is located on the left.
	2	Time Step	Time based location of the step between two plateaus. *Important:* Notably, if the time resolution was not provided in the Run Settings box, the time is converted to indices.
	3	Level Before	Level of the plateau before the step occurred.
	4	Level After	Level of the plateau after the step occurred.
	5	Step Size	Signal difference between the two plateaus. Notably, a negative step size indicates a step from a higher level to a lower level, whereas a positive step size indicates a step from a lower to a higher level.
	6	Dwell Time Step Before	Dwell time of the plateau before the step occurred.
	7	Dwell Time Step After	Dwell time of the plateau after the step occurred.

	8	Error	Predicted error of the step size, which is based on the plateau length and step size.
	9	Bootstrap error of the step size	The 95% confidence interval of the step size determined by bootstrap analysis
	10	Bootstrap error of the time	The 95% confidence interval of the time determined by bootstrap analysis
filename_SCurve	1	Step Number	The number of steps that have been fitted to the data.
	2	SCurve Round 1	S-values of the first round of fitting.
	3	SCurve Round 2	S-values of the second round of fitting.
filename_config	-	-	A list of all the fitting parameters that were used by *AutoStepfinder*.
filename_fitfig (exclusive for batch analysis)	-	-	An .JPEG image of the fitting window (11.A), showing the raw data and fit.
filename_s_curve (exclusive for batch analysis)	-	-	An .JPEG image of the s-curve window (11.E3), showing S-curves of round 1 and 2.
filename_user_plot (exclusive for batch analysis)	-	-	An .JPEG image of the userplot window (11.G), showing the plots of step-size and step-levels.

Table S2: Troubleshooting *AutoStepfinder*

Step	Problem	Possible reason	Solution
6A	Pop-up with Error: The provided directory is not valid.	The provided directory does not exist.	Provide an existing data path.
		The provided directory is not a folder.	Provide a data path to the file directory.
6C	Popup with Error: The input for the number of columns is NaN.	The input for the number of columns is not a number (NaN).	Provide a number as input for the number of columns.
7	Popup with Error: The provided input folder is empty.	The provided input folder is empty.	Select a folder that contains .txt files with your data.
	Pop-up with Error: 'FileName' contains is not formatted properly.	The data may be the wrong filetype.	Check the file extension of the input data. The file extension should be .txt.
		The data may contain characters	Alternatively, check if the input data contains characters.
10	The *AutoStepfinder* GUI does is not displayed as in Figure 8.	Screen resolution is too low.	Increase screen resolution. Alternatively, resize GUI window.
		Monitor size is smaller than 17".	Connect a larger monitor to your computer. Alternatively, resize GUI window.
11.B	Pop-up with Error: The provided directory is not valid.	The provided directory does not exist.	Provide an existing data path.
		The provided directory is not a folder.	Provide a data path to the file directory.
11.C	Popup with Error: Empty folder.	The provided input folder is empty.	Select a folder that contains .txt files with your data.
11D.1	Popup with Error: The iteration range parameter is NaN.	The input for the iteration range parameter is not a number (NaN).	Provide a number as input for iteration range parameter.
11D.2	Popup with Error: The time resolution parameter is NaN.	The input for the time resolution parameter is not a number (NaN).	Provide a number as input for the time resolution parameter.
11D.3	Popup with Error: The acceptance threshold is NaN.	The input for the acceptance threshold is not a number (NaN).	Provide a number as input for the acceptance threshold parameter.
11.G4	Popup with Error: The input for manual mode is NaN.	The input for the manual mode parameter is not a number (NaN).	Provide a number as input for the manual mode parameter.
	Popup with Error: The input for manual mode is smaller than 1.	The input for manual mode parameter is smaller than 1.	Provide a parameter value for manual mode that is larger than 1.
11.G5	Popup with Error: The mean baseline parameter is NaN.	The input for the mean baseline parameter is not a number (NaN).	Provide a number as input for the baseline parameter.
11.G6	Popup with Error: The time range for noise estimation is NaN.	The input for the time range for noise estimation is not a number (NaN).	Provide a number as input for the time range parameter.
	Pop-up with Error: 'FileName' contains more than two columns.	The input data is containing more than 2 columns.	Provide a .txt file with a single or double column. Multi-column files can be split

			into single columns using DataDuster. see step 3 to 9.
14	Pop-up with Error: 'FileName' contains NaN values.	Data contains values that are not a number (NaN).	Remove or replace NaN data point(s) by using DataDuster see step 3 to 9.
	Pop-up with Error: 'FileName' contains infinite values.	Data contains values that are infinite (Inf).	Remove or replace Inf data point(s) by using the DataDuster see step 3 to 9.
	Pop-up with Error: 'FileName' contains is not formatted properly.	The data may be the wrong filetype.	Check the file extension of the input data, which should be .txt.
		The data may contain characters	Alternatively, check if the input data contains characters.
	Pop-up: No significant steps detected.	Detected S^{max} of round 1 is below the acceptance threshold.	Decrease the acceptance threshold parameter in advanced settings (Step 11.D3).
15		Data does not contain significant steps.	An inherent feature of the input data, e.g. linear data, that cannot be solved.
	AutoStepfinder does not detect a significant portion of my events.	Base line type events: The baseline between events is too long.	Decrease the baseline between events. A typical range for effective step detection are baselines that have an equal or smaller dwell time than the events.
		Data exhibits big features (large-step sizes or long plateaus) that are not of interest.	Remove or decrease size of these features from dataset.
		Events are too short or too small to fulfill the S-criteria.	Inspect S-curve and if necessary tune fitting with sensitivity or manual mode.
	AutoStepfinder splits big steps into smaller unwanted steps.	Steps in the data are not instantaneous.	An inherent feature of the input data, no obvious solution.
		Data was low pass filtered or smoothened.	*AutoStepfinder* works best on instant steps, remove smoothing of data. Alternatively, consider step preserving filtering, such as median or Chung-Kennedy filtering.
	AutoStepfinder fits high frequency features that are not of interest.	Data contains high frequency features.	Consider step preserving filtering, such as median or Chung-Kennedy filtering. Alternatively, consider removing the features corresponding to the high frequency features from the fit. For example, by using the StepMerger auxiliary tool.
